# Family Size and Longitudinal Outcomes of a Digital-Human Parenting Intervention in Chinese Preschool Families: Secondary Analysis

**DOI:** 10.2196/101388

**Published:** 2026-07-10

**Authors:** Zuyi Fang, Xing He, Xinyu Shi, Ruochen Ruan, Jamie M Lachman

**Affiliations:** 1 Institute of Population Research, Peking University, No. 5 Yiheyuan Road, Haidian District, Beijing, 100871, China, 86 13716165860; 2 Department of Social Policy and Intervention, University of Oxford, Oxford, United Kingdom; 3 Department of Sociology, Peking University, Beijing, China; 4 Faculty of Education, Beijing Normal University, Beijing, China; 5 Centre for Social Science Research, University of Cape Town, Rondebosch, Cape Town, South Africa; 6 Parenting for Lifelong Health, Barnett House, Oxford, United Kingdom

**Keywords:** family size, number of children, parenting interventions, digital intervention, longitudinal trajectories

## Abstract

**Background:**

Parenting interventions can improve parental and child outcomes across diverse settings. However, less is known about how family size, including the number of children, shapes baseline conditions, and how intervention effects unfold over time. Most studies also focus on average treatment effects, with limited attention to heterogeneity across family contexts and trajectories of change.

**Objective:**

This study examined whether the number of children was associated with baseline differences in parental and child outcomes, moderated immediate postintervention effects, and shaped postintervention trajectories over 6- and 12-month follow-up periods.

**Methods:**

We conducted secondary analysis of a pragmatic cluster randomized controlled trial evaluating a universal digital-human parenting intervention delivered through the preschool system in China (N=541). Families were categorized by the number of children (1, 2, 3, or more). We examined (1) baseline differences in parental and child outcomes, (2) moderation of intervention effectiveness at immediate postintervention, and (3) trajectories of change over 6- and 12-month follow-up periods using mixed-effects models.

**Results:**

Of the 541 enrolled families, 494 were included in the complete-case baseline analysis. Compared with 1-child families, 2-child families, and families with 3 or more children, reported lower levels of baseline early learning and stimulation and proactive parenting, as well as greater endorsement of corporal punishment and higher parenting stress. We found no statistically significant evidence that the number of children moderated immediate postintervention effects. In intervention-group trajectory analyses, 2-child families showed greater improvement in early learning and stimulation at the 6-month follow-up (b=3.966, 95% CI 1.468-6.463). Families with 3 or more children showed a similar pattern (b=5.749, 95% CI 0.536-10.962), although estimates for this subgroup were less precise because of the small sample size. This subgroup also showed larger but more variable reductions in selected child behavioral outcomes over follow-up.

**Conclusions:**

Family size might not always be associated with short-term intervention effectiveness but was associated with divergence in longer-term trajectories. These findings suggest that caregiving demands are relevant for the sustainability of intervention effects. By integrating baseline differences, short-term effects, and longitudinal trajectories within a single framework, this study highlights the importance of moving beyond average treatment effects to more dynamic, context-sensitive evaluations. Designing parenting interventions, particularly scalable digital-human programs, that incorporate sustained and context-responsive support may be critical for addressing variation in family structure and enhancing long-term effectiveness.

## Introduction

Parenting interventions are widely recognized as effective strategies for improving parenting practices and child developmental outcomes. Substantial evidence shows that these interventions can reduce child emotional and behavioral problems, enhance positive parenting, improve parent-child relationships, alleviate parental stress and anxiety, and reduce child maltreatment across diverse socioeconomic groups and disability statuses [1-8].

However, less is known about how intervention effects vary by family size, particularly the number of children within the household, as such interventions are often designed and evaluated in ways that emphasize average treatment effects [9-11]. Family size is typically included as a control variable rather than examined as a source of variation in intervention processes and outcomes, limiting insight into how interventions operate across families.

Although a small number of earlier studies have examined this issue, the evidence remains limited and mixed. Meta-analytic findings suggest that larger families may show smaller improvements in child externalizing behaviors, potentially due to challenges in maintaining consistent parenting practices across multiple children [12], and that logistical and time constraints may reduce the intensity of intervention implementation in multichild households [13]. However, other evaluations report no significant moderation by family size, indicating that program design and intensity may mitigate such constraints [14].

Several theoretical perspectives provide a basis for examining family size in parenting interventions. From a resource-based perspective, classic economic models of the family highlight a trade-off between the number of children and the level of investment (eg, time, attention, and emotional energy) per child [15]. This “resource dilution” mechanism has been used to explain associations between sibship size and a range of child outcomes, such as educational attainment, cognitive development, and physical growth [16-19]. For example, empirical studies on early childhood development show that children from larger families tend to exhibit lower levels of lexical diversity, consistent with the idea that language development—reliant on high-quality, one-to-one interactions—may be diluted when parental attention is divided across multiple children [20].

At the same time, family systems perspectives suggest that family size is also associated with changes in family dynamics. The presence of multiple children introduces additional relational subsystems, particularly sibling interactions, which may influence parenting practices and the broader family environment. These subsystems interact with parent-child relationships in dynamic ways, such that changes in one domain may spill over into others [21]. Managing sibling relationships, including conflict and cooperation, may therefore add an additional layer of parenting demands that is largely absent in single-child families [22]. Nonetheless, these dynamics may also create opportunities for generalization, as parenting strategies learned for one child can extend to siblings, producing broader family-level changes [23].

Family systems perspectives also point to potential adaptive processes. Parents with multiple children may accumulate experience, potentially enhancing parenting skills through repeated practice. These opportunities for repeated application may strengthen skill acquisition and confidence over time. Empirical evidence is mixed, with some studies linking larger family size to higher parenting stress and lower parenting quality, and others suggesting adaptive or compensatory processes [24,25].

Therefore, family size may be associated with both constraints and opportunities in caregiving and is relevant for understanding variation in parenting interventions. However, its role in shaping baseline conditions and intervention responses over time remains underexplored. This study aims to examine whether family size, particularly with respect to the number of children, is associated with (1) differences in baseline parental and child outcomes, (2) variation in intervention effectiveness at immediate postintervention, and (3) differences in impact trajectories over 6- and 12-month follow-up periods.

These questions are particularly relevant in the context of contemporary China, where family structures have undergone substantial changes in recent years. While policy shifts, including the introduction of the 2-child and 3-child policies, have altered the institutional context, broader factors such as fertility preferences, child-rearing costs, educational competition, and changing parenting norms also contribute to variation in the number of children in a household [26,27]. As a result, 1-child and multichild families coexist within the same social environment, creating considerable heterogeneity in caregiving contexts.

## Methods

### Study Design and Sample

This study is a secondary analysis of data from a pragmatic, 2-arm, single-blind cluster randomized controlled trial evaluating a universal digital-human parenting intervention delivered through the preschool system in China [28].

The trial was conducted in a large public preschool in a lower-middle-income city in Jiangxi Province, central China. All 21 classes within the preschool were included as clusters and randomly assigned in a 1:1 ratio to either the intervention group (10 classes) or a waitlist control group (11 classes) using a computer-generated sequence by an independent researcher.

Eligible participants were primary caregivers of children aged 3 to 6 years enrolled in the preschool. Inclusion criteria were minimal to reflect the universal nature of the intervention: caregivers had to be aged 18 years or older, have access to a smartphone, and provide informed consent. No additional exclusion criteria were applied. Caregivers were recruited through routine school communication channels, with one caregiver participating per family.

A total of 541 caregivers were enrolled, including 272 in the intervention group and 269 in the control group. Caregivers had a mean age of 36.6 (SD 5.4) years, and 74.8% (404/541) were female. Children had a mean age of 4.9 (SD 0.9) years, and households had an average of 1.9 (SD 0.6) children.

Data were collected at 4 time points: baseline (T0), immediate postintervention (T1), 6-month follow-up (T2), and 12-month follow-up (T3). Due to the waitlist design, the control group received the intervention after T1 and was not followed as a comparison group at later time points. Accordingly, between-group comparisons are limited to postintervention effects (T1), while longer-term changes (T2-T3) are examined within the intervention group. The participant flow is presented in Multimedia Appendix 1.

### Intervention Procedure

The intervention was a universal digital-human parenting program, named “Keyushiguang,” delivered through the preschool system. It was based on the Parenting for Lifelong Health (PLH) programs and adapted from the PLH ParentText intervention for delivery via WeChat (Tencent Holdings Limited).

The program comprised 37‐39 (depending on child age) brief interactive chatbot modules delivered over approximately 2.5 months, covering 8 core parenting topics (eg, parent-child relationships, child development, behavior management, and child safety). Content was delivered daily in a self-directed format and tailored to caregiver and child characteristics.

The digital content was complemented by human support, including weekly or twice-weekly message-based group discussions facilitated by preschool headteachers and social workers within class-based WeChat groups, to reinforce key messages, support reflection, and encourage peer interaction.

Additional strategies included facilitator reminders and a child-centered incentive mechanism linking caregiver participation to small rewards for the participating children. The incentive was implemented at the family or index-child level rather than separately for each child in the household. Together, these components were designed to promote sustained engagement and integration of parenting practices into daily routines. Further details about the intervention are provided in related publications [28,29].

The intervention group received the program following baseline assessment, while the waitlist control group received no formal support during this period and began the intervention after the postintervention assessment.

### Measures

#### Core Exposure

The primary exposure was the number of children in the household, assessed at baseline and modeled categorically with 3 levels: 1 child, 2 children, and 3 or more children.

#### Intervention Outcomes

All outcomes were based on caregiver self-report using validated instruments with established psychometric properties in Chinese populations. Primary outcomes included early learning and stimulation, assessed using items from the Multiple Indicator Cluster Surveys (MICS), and caregiver-perpetrated violence, measured using the International Society for the Prevention of Child Abuse and Neglect (ISPCAN) Child Abuse Screening Tool (ICAST). Secondary outcomes captured broader domains of parenting and child functioning, including child behavior problems (Strengths and Difficulties Questionnaire [SDQ]), parenting practices (Alabama Parenting Questionnaire [APQ]), parental mental health (Depression Anxiety Stress Scales-21 [DASS-21]), parenting stress (Parental Stress Scale [PSS]), and family functioning (Family Adaptability, Partnership, Growth, Affection, and Resolve). Attitudes toward corporal punishment were assessed using a single item from MICS. Detailed descriptions of all measures and their psychometric properties are provided in Multimedia Appendix 1.

#### Sociodemographic Context Variables

Baseline sociodemographic variables were included to characterize the sample and adjust for potential confounding, based on prior literature on parenting and intervention heterogeneity. Caregiver characteristics included age, sex, education level, employment status, marital status, ethnicity, and disability status. Child characteristics included age, sex, and disability status. Household-level variables included household registration (urban vs rural).

### Statistical Analysis

Baseline characteristics and outcomes were summarized by number of children using means (SD) for continuous variables and frequencies (%) for categorical variables. Group differences were assessed using 1-way ANOVA or chi-square tests. For the missing data, we assessed whether the missingness pattern was consistent with the assumption of missing completely at random (MCAR) using the Little MCAR test. We also summarized the extent and pattern of missing baseline data overall and by trial arm and number of children. Differences between families included in and excluded from complete-case baseline analyses were assessed using chi-square or Fisher exact tests for categorical variables and 2-tailed *t* tests or 1-way ANOVA for continuous variables, as appropriate. Multivariable regression models, adjusted for sociodemographic covariates, were used to examine baseline associations between the number of children and outcomes. To account for the potential influence of extended family support on parenting and resource allocation, we additionally adjusted for grandparent caregiving in baseline regression models.

Moderation of intervention effectiveness at postintervention was analyzed following the intention-to-treat principle. We used multivariable mixed-effects models (linear, Poisson, or negative binomial, depending on outcome distribution) to account for clustering at the class level and repeated assessments within families (random intercepts). Model selection for violence perpetration outcomes was informed by the distribution test (Table S1 in Multimedia Appendix 1). Moderation by family size was assessed using a group×time×family size interaction term. Subgroup analyses stratified by number of children were also conducted, and sensitivity analyses treated family size as a continuous variable.

The moderation analysis assessed whether family size modified the average short-term intervention effect, defined as the between-group difference in change from baseline to immediate postintervention. This differs from the trajectory analysis, which examined whether families in the intervention group followed different patterns of change over time after receiving the program. These analyses address related but distinct questions and may therefore yield different findings. Specifically, moderation analyses focus on average short-term treatment effects, whereas trajectory analyses examine patterns of change over time. Differences between the two may emerge when intervention responses are nonlinear, delayed, or differentially sustained.

For the intervention group, longitudinal trajectories from T0 to T3 were examined using mixed-effects models including time, family size, and their interaction, adjusting for baseline sociodemographic covariates. We additionally adjusted for grandparent caregiving in the longitudinal regression models to account for the potential influence of extended family support. Furthermore, 2 sensitivity analyses were conducted to assess the robustness of the findings: (1) estimating unadjusted longitudinal models including only time, number of children, and their interaction; and (2) applying multiple imputation by chained equations (MICE) with 30 imputed datasets to address missing data in the fully adjusted models. Estimated marginal means were used to visualize adjusted trajectories over time. These models above were interpreted as within-intervention-group postintervention trajectories.

To contextualize the limited precision of the moderation and trajectory analyses, particularly for families with 3 or more children, we calculated minimum detectable effect sizes (MDES) for key interaction terms. MDES were calculated from the model-based SEs of the interaction estimates, assuming a 2-sided α of .05 and 80% power. For continuous outcomes, MDES are presented as absolute differences in scale units. For count outcomes, MDES were reported as incidence rate ratios (IRRs) after backtransformation from the log scale. These estimates were used to quantify the smallest interaction effects the study was adequately powered to detect rather than to reinterpret statistical significance. All models used robust SEs. A 2-tailed α level of .05 was used to determine statistical significance. Analyses were conducted using Stata 17.0 (StataCorp, LLC) and R (R Foundation for Statistical Computing; refer to Multimedia Appendix 1 for detailed statistical methods).

### Ethical Considerations

Ethical approval for the original trial was obtained from Beijing Normal University (SSDPP-HSC-2024003) and the University of Oxford (SPI DREC 25 006). The trial was prospectively registered on the Chinese Clinical Trial Registry (ChiCTR2400081911). All participants provided informed consent prior to data collection. This study is a secondary analysis of deidentified trial data and was conducted in accordance with the original approvals; no additional ethical approval was required.

## Results

### Baseline Differences by Number of Children

#### Descriptive Statistics

Table 1 presents baseline outcome variables by number of children, and Table 2 presents baseline sociodemographic characteristics (N=494). Of the 541 families enrolled at baseline, 47 were excluded because the baseline sociodemographic information required for the analysis was incomplete. Little MCAR test did not reject the MCAR assumption for baseline sociodemographic covariates (*χ*²_108_=124.04, *P*=.14). Exclusion did not differ by trial arm: 22 of 269 (8.18%) families in the control group and 25 of 272 (9.19%) families in the intervention group were excluded (*χ*²_1_=0.17, *P*=.68). Among families with nonmissing information on the number of children, exclusion differed by family size: 10 of 130 (7.69%) with 1-child families, 24 of 364 (6.59%) with 2-child families, and 11 of 45 (24.44%) families with 3 or more children were excluded (Fisher exact *P*=.001). Two families had missing information on the number of children and were therefore not included in the family size–specific comparison. To address the potential risk of missing-at-random bias associated with differential exclusion by number of children, we conducted sensitivity analyses using MICE in the longitudinal analyses.

**Table 1.** Baseline outcomes characteristics by number of children in the full sample (N=494)^a^.

Variables	Number of children				*P* value
	Total sample (N=494)	1 child (n=120)	2 children (n=340)	3 or more children (n=34)	
Primary outcomes, mean (SD)					
Early learning and stimulation	21.94 (9.29)	24.46 (8.87)	21.28 (9.23)	19.59 (9.85)	.002
Caregiver-perpetrated violence: total	14.31 (5.54)	14.19 (5.54)	14.25 (5.45)	15.29 (6.46)	.56
Caregiver-perpetrated violence: physical	5.58 (2.58)	5.47 (2.54)	5.62 (2.60)	5.62 (2.57)	.86
Caregiver-perpetrated violence: emotional	8.73 (3.74)	8.73 (3.56)	8.63 (3.59)	9.68 (5.50)	.30
Secondary outcomes, mean (SD)					
Attitude toward corporal punishment	2.66 (1.33)	2.37 (1.22)	2.74 (1.33)	2.94 (1.52)	.01
Child behavior: total	12.15 (4.02)	12.30 (4.10)	12.02 (3.99)	12.88 (4.04)	.44
Child behavior: internalizing	5.55 (2.09)	5.72 (2.18)	5.48 (2.09)	5.65 (1.86)	.55
Child behavior: externalizing	6.60 (2.97)	6.58 (3.07)	6.54 (2.93)	7.24 (2.99)	.42
Child behavior: emotional problem	3.68 (1.38)	3.76 (1.32)	3.65 (1.41)	3.68 (1.32)	.77
Child behavior: conduct problem	2.26 (1.31)	2.24 (1.26)	2.24 (1.30)	2.53 (1.58)	.47
Child behavior: hyperactivity	4.33 (2.28)	4.34 (2.43)	4.29 (2.24)	4.71 (2.10)	.60
Child behavior: peer problem	1.87 (1.36)	1.96 (1.43)	1.83 (1.34)	1.97 (1.24)	.61
Child behavior: prosocial behavior	7.29 (1.83)	7.28 (1.80)	7.28 (1.86)	7.50 (1.66)	.79
Parenting practices: total	56.83 (7.15)	58.38 (7.05)	56.41 (7.21)	55.47 (6.32)	.02
Parenting practices: positive	24.02 (3.32)	24.47 (3.38)	23.91 (3.34)	23.59 (2.89)	.21
Parenting practices: involvement	32.81 (4.77)	33.91 (4.52)	32.51 (4.81)	31.88 (4.80)	.01
Parental mental health: total	3.61 (4.88)	3.25 (4.39)	3.71 (5.15)	3.94 (3.60)	.63
Parental mental health: depression	1.77 (2.87)	1.42 (2.24)	1.91 (3.11)	1.65 (2.17)	.27
Parental mental health: anxiety	1.84 (2.67)	1.83 (2.91)	1.80 (2.62)	2.29 (2.26)	.59
Parenting stress	38.44 (7.03)	36.42 (6.83)	38.90 (6.90)	41.03 (7.52)	<.001
Family functioning	2.64 (2.47)	2.58 (2.41)	2.67 (2.50)	2.53 (2.36)	.90

a Values are presented as mean (SD) for continuous variables and n (%) for categorical variables. *P* values were obtained using 1-way ANOVA for continuous variables and chi-square tests for categorical variables, as appropriate. Percentages may not sum to 100 because of rounding. Baseline characteristics are presented for the analytic sample with nonmissing sociodemographic information (N=494).

**Table 2.** Baseline sociodemographic characteristics by number of children in full sample (N=494)^a^.

Variables	Number of children				*P* value
	Total sample (N=494)	1 child (n=120)	2 children (n=340)	3 or more children (n=34)	
Marital status, n (%)					.53
Single	6 (1.21)	3 (2.50)	3 (0.88)	0 (0.00)	
Unmarried but not single	2 (0.40)	1 (0.83)	1 (0.29)	0 (0.00)	
Married	486 (98.38)	116 (96.67)	336 (98.82)	34 (100.00)	
Hukou, n (%)					.29
Rural	159 (32.19)	39 (32.50)	105 (30.88)	15 (44.12)	
Urban	335 (67.81)	81 (67.50)	235 (69.12)	19 (55.88)	
Adult’s sex, n (%)					.64
Male	124 (25.10)	34 (28.33)	82 (24.12)	8 (23.53)	
Female	370 (74.90)	86 (71.67)	258 (75.88)	26 (76.47)	
Children’s sex, n (%)					.12
Male	277 (56.07)	58 (48.33)	201 (59.12)	18 (52.94)	
Female	217 (43.93)	62 (51.67)	139 (40.88)	16 (47.06)	
Adult age, mean (SD)	36.64 (5.39)	33.58 (4.16)	37.34 (5.16)	40.41 (6.64)	<.001
Child age, mean (SD)	5.71 (0.99)	5.47 (1.08)	5.83 (0.95)	5.41 (0.86)	<.001
Education, mean (SD)	4.50 (0.92)	4.65 (0.80)	4.50 (0.90)	3.94 (1.25)	<.001
Employment status, n (%)					.003
Full time	401 (81.17)	98 (81.67)	284 (83.53)	19 (55.88)	
Part time	13 (2.63)	4 (3.33)	8 (2.35)	1 (2.94)	
Unemployed	33 (6.68)	5 (4.17)	20 (5.88)	8 (23.53)	
Self-employed	40 (8.10)	10 (8.33)	25 (7.35)	5 (14.71)	
Other	7 (1.42)	3 (2.50)	3 (0.88)	1 (2.94)	
Ethnicity, n (%)					.15
Han	491 (99.39)	120 (100.00)	338 (99.41)	33 (97.06)	
Other	3 (0.61)	0 (0.00)	2 (0.59)	1 (2.94)	
Children functional difficulty, n (%)					.46
Yes	119 (24.09)	30 (25.00)	78 (22.94)	11 (32.35)	
No	375 (75.91)	90 (75.00)	262 (77.06)	23 (67.65)	

a Note: Values are presented as mean (SD) for continuous variables and n (%) for categorical variables. *P* values were obtained using 1-way ANOVA for continuous variables and chi-square tests for categorical variables, as appropriate. Percentages may not sum to 100 because of rounding. Baseline characteristics are presented for the analytic sample with nonmissing sociodemographic information (N=494).

In the analytic sample, 120 out of 494 (24.29%) families had 1 child, 340 out of 494 (68.83%) had 2 children, and 34 out of 494 (6.88%) had 3 or more children. Most caregivers were married (486/494, 98.38%), female (370/494, 74.90%), of Han ethnicity (491/494, 99.39%), and had urban household registration (ie, hukou 335/494, 67.81%). Among children, 56.07% (277/494) were boys, and 24.09% (119/494) had functional difficulties.

Caregiver age differed significantly by family size, with caregivers in families with 3 or more children being older than those in 1- and 2-child families (40.41 vs 33.58 and 37.34 years, respectively; *P*<.001). Educational attainment was lower in larger families (3.94 vs 4.65 and 4.50, *P*<.001). Employment status also varied across groups (*P*=.003), with families with 3 or more children having the lowest proportion of full-time employment (19/34, 55.88%) and the highest proportion of unemployment (8/34, 23.53%). The age of the targeted child also differed significantly by the number of children (*P*<.001).

For the primary outcomes, differences by number of children were observed for early learning and stimulation but not for caregiver-perpetrated violence. Early learning and stimulation were highest among 1-child families and decreased with increasing family size (24.46, 21.28, and 19.59, respectively; *P*=.002).

For the secondary outcomes, differences by number of children were observed in attitudes toward corporal punishment, total parenting practices, parental involvement, and parenting stress. Attitudes endorsing corporal punishment increased with the number of children (2.37, 2.74, and 2.94 for 1-child, 2-child, and 3 or more children families, respectively; *P*=.01). In contrast, total parenting practices and parental involvement were highest among 1-child families and declined with increasing number of children (total parenting practices: 58.38, 56.41, and 55.47; *P*=.02; parental involvement: 33.91, 32.51, and 31.88; *P*=.01). Parenting stress also increased with the number of children (36.42, 38.90, and 41.03, respectively; *P*<.001). No significant baseline differences by number of children were observed for child behavior, parental mental health, positive parenting, or family functioning.

#### Baseline Regression: Differences in Parental and Child Outcomes

Table 3 presents the baseline associations between the number of children and parental and child outcomes in the analytic sample.

For the primary outcomes, adjusted regression models showed that, compared with 1-child families, 2-child families had lower levels of early learning and stimulation (b=−3.292, 95% CI −5.197 to −1.387; *P*=.003), and families with 3 or more children showed similarly lower levels (b=−5.878, 95% CI −10.053 to −1.703; *P*=.01). No differences were observed for caregiver-perpetrated violence.

For the secondary outcomes, compared with 1-child families, 2-child families had more depressive symptoms (b=0.576, 95% CI 0.135-1.017; *P*=.02), fewer proactive parenting practices (b=−1.824, 95% CI −3.329 to −0.319; *P*=.03), less parental involvement (b=−1.392, 95% CI −2.292 to −0.492; *P*=.007), greater endorsement of corporal punishment (b=0.459, 95% CI 0.161-0.757*;P*=.007), and more parenting stress (b=2.388, 95% CI 0.712-4.064; *P*=.01). Families with 3 or more children also showed greater endorsement of corporal punishment (b=0.748, 95% CI 0.131-1.365; *P*=.03) and more parenting stress (b=3.921, 95% CI 0.415-7.427; *P*=.04). No differences were observed for overall parental mental health, anxiety, child behavior outcomes, positive parenting, or family functioning.

Furthermore, the baseline associations between the number of children and all key outcomes remained highly robust even after additionally adjusting for grandparent caregiving (Table S3 in Multimedia Appendix 1).

**Table 3.** The baseline association between the number of children and outcomes in the full sample (N=494)^a^.

Outcomes and number of children (Ref. 1 child)	Estimate (95% CI)	*P* value
Primary outcomes		
Early learning and stimulation		
2 children	−3.292 (−5.197 to −1.387)	.003
≥3 children	−5.878 (−10.053 to −1.703)	.01
Caregiver-perpetrated violence: total		
2 children	0.033 (−0.059 to 0.116)	.48
≥3 children	0.095 (−0.065 to 0.234)	.25
Caregiver-perpetrated violence: physical		
2 children	1.061 (0.938 to 1.184)	.32
≥3 children	1.014 (0.814 to 1.214)	.89
Caregiver-perpetrated violence: emotional		
2 children	1.013 (0.913 to 1.113)	.80
≥3 children	1.167 (0.932 to 1.402)	.14
Secondary outcomes		
Parental mental health: total		
2 children	0.582 (−0.494 to 1.658)	.30
≥3 children	0.783 (−0.924 to 2.490)	.38
Parental mental health: depression		
2 children	0.576 (0.135 to 1.017)	.02
≥3 children	0.375 (−0.513 to 1.263)	.42
Parental mental health: anxiety		
2 children	0.006 (−0.731 to 0.743)	.99
≥3 children	0.408 (−0.695 to 1.511)	.48
Child behavior: total		
2 children	−0.236 (−1.151 to 0.679)	.62
≥3 children	0.080 (−1.831 to 1.991)	.94
Child behavior: emotional problem		
2 children	−0.019 (−0.329 to 0.291)	.91
≥3 children	−0.006 (−0.451 to 0.439)	.98
Child behavior: conduct problem		
2 children	0.071 (−0.243 to 0.385)	.66
≥3 children	0.367 (−0.252 to 0.986)	.26
Child behavior: hyperactivity		
2 children	−0.232 (−0.800 to 0.336)	.43
≥3 children	−0.271 (−1.239 to 0.697)	.59
Child behavior: peer problem		
2 children	−0.056 (−0.403 to 0.291)	.75
≥3 children	−0.010 (−0.586 to 0.566)	.97
Child behavior: prosocial behavior		
2 children	0.130 (−0.323 to 0.583)	.58
≥3 children	0.590 (−0.135 to 1.315)	.13
Child behavior: externalizing behavior		
2 children	−0.161 (−0.902 to 0.580)	.68
≥3 children	0.096 (−1.352 to 1.544)	.89
Child behavior: internalizing behavior		
2 children	−0.075 (−0.630 to 0.480)	.79
≥3 children	−0.015 (−0.823 to 0.793)	.97
Parenting practices: total		
2 children	−1.824 (−3.329 to −0.319)	.03
≥3 children	−1.981 (−4.980 to 1.018)	.21
Parenting practices: positive parenting		
2 children	−0.433 (−1.217 to 0.351)	.29
≥3 children	−0.574 (−1.779 to 0.631)	.36
Parenting practices: parental involvement		
2 children	−1.392 (−2.292 to −0.492)	.007
≥3 children	−1.407 (−3.865 to 1.051)	.28
Attitude toward corporal punishment		
2 children	0.459 (0.161 to 0.757)	.007
≥3 children	0.748 (0.131 to 1.365)	.03
Parenting stress		
2 children	2.388 (0.712 to 4.064)	.01
≥3 children	3.921 (0.415 to 7.427)	.04
Family functioning		
2 children	−0.135 (−0.560 to 0.290)	.54
≥3 children	−0.597 (−1.414 to 0.220)	.17

a All models were adjusted for caregiver age, child age, marital status, caregiver gender, child gender, educational attainment, ethnicity, hukou status, employment status, and child disability. Total maltreatment was estimated using negative binomial regression, whereas physical abuse and emotional abuse were estimated using Poisson regression and are reported as incidence rate ratios. Baseline characteristics are presented for the analytic sample with nonmissing sociodemographic information (N=494). Of the 541 families enrolled at baseline, 47 were not included in this table because of missing baseline sociodemographic data.

### Moderation of Intervention Effectiveness at Postintervention

#### Primary Outcomes: Moderator Analysis

We did not detect statistically significant group×time×number of children interactions for the primary outcomes (Table 4). For early learning and stimulation, the group×time×number of children interaction was not statistically significant (*P*=.92), and interaction terms for 2-child and 3-or-more-child families did not differ from the 1-child reference group.

Similarly, no significant moderation by number of children was observed for caregiver-perpetrated violence, including total, physical, and emotional violence (*P*=.92, .72, and .92, respectively). Interaction estimates were close to the null.

**Table 4.** Moderation of immediate postintervention effects by the number of children for outcomes (N=1039)^a^.

Outcomes and three-way interaction terms	Estimate (95% CI)	*P* value	*P* value for interaction^b^
Primary outcomes			
Early learning and stimulation			.92
Group×time×2 children	0.590 (−3.045 to 4.225)	.75	
Group×time×≥3 children	1.159 (−5.343 to 7.660)	.73	
Caregiver-perpetrated violence: total			.92
Group×time×2 children	1.007 (0.717 to 1.416)	.97	
Group×time×≥3 children	0.845 (0.382 to 1.866)	.68	
Caregiver-perpetrated violence: physical			.72
Group×time×2 children	0.889 (0.531 to 1.486)	.66	
Group×time×≥3 children	0.614 (0.170 to 2.221)	.46	
Caregiver-perpetrated violence: emotional			.92
Group×time×2 children	1.069 (0.776 to 1.474)	.68	
Group×time×≥3 children	1.002 (0.440 to 2.284)	.99	
Secondary outcomes			
Attitude toward corporal punishment			.76
Group×time×2 children	0.207 (−0.334 to 0.748)	.45	
Group×time×≥3 children	0.162 (−0.804 to 1.129)	.74	
Child behavior: total			.24
Group×time×2 children	−0.425 (−1.940 to 1.089)	.58	
Group×time×≥3 children	−2.341 (−5.053 to 0.370)	.09	
Child behavior: internalizing behavior			.41
Group×time×2 children	−0.358 (−1.269 to 0.553)	.44	
Group×time×≥3 children	−1.082 (−2.710 to 0.546)	.19	
Child behavior: externalizing behavior			.41
Group×time×2 children	−0.059 (−1.093 to 0.974)	.91	
Group×time×≥3 children	−1.195 (−3.047 to 0.657)	.21	
Child behavior: emotional problem			.89
Group×time×2 children	0.014 (−0.571 to 0.598)	.96	
Group×time×≥3 children	−0.229 (−1.275 to 0.816)	.67	
Child behavior: conduct problem			.45
Group×time×2 children	0.076 (−0.487 to 0.640)	.79	
Group×time×≥3 children	−0.519 (−1.526 to 0.488)	.31	
Child behavior: hyperactivity			.64
Group×time×2 children	−0.139 (−0.944 to 0.666)	.74	
Group×time×≥3 children	−0.689 (−2.131 to 0.752)	.35	
Child behavior: peer problem			.28
Group×time×2 children	−0.364 (−0.987 to 0.259)	.25	
Group×time×≥3 children	−0.844 (−1.955 to 0.268)	.14	
Child behavior: prosocial behavior			.56
Group×time×2 children	0.264 (−0.468 to 0.997)	.48	
Group×time×≥3 children	−0.307 (−1.617 to 1.004)	.65	
Parenting practices: total			.65
Group×time×2 children	0.012 (−3.192 to 3.216)	.99	
Group×time×≥3 children	−2.454 (−8.178 to 3.269)	.40	
Parenting practices: positive parenting			.65
Group×time×2 children	−0.706 (−2.269 to 0.857)	.38	
Group×time×≥3 children	−0.906 (−3.693 to 1.882)	.52	
Parenting practices: parental involvement			.38
Group×time×2 children	0.723 (−1.335 to 2.782)	.49	
Group×time×≥3 children	−1.529 (−5.208 to 2.151)	.42	
Parental mental health: total			.97
Group×time×2 children	−0.160 (−2.179 to 1.859)	.88	
Group×time×≥3 children	0.218 (−3.396 to 3.831)	.91	
Parental mental health: depression			.97
Group×time×2 children	0.079 (−1.177 to 1.334)	.90	
Group×time×≥3 children	0.284 (−1.960 to 2.529)	.80	
Parental mental health: anxiety			.89
Group×time×2 children	−0.255 (−1.346 to 0.836)	.648	
Group×time×≥3 children	−0.077 (−2.030 to 1.877)	.94	
Parenting stress			.31
Group×time×2 children	1.306 (−1.624 to 4.235)	.38	
Group×time×≥3 children	−2.081 (−7.318 to 3.155)	.44	
Family functioning			.17
Group×time×2 children	0.038 (−1.024 to 1.101)	.94	
Group×time×≥3 children	−1.640 (−3.539 to 0.259)	.09	

a Three-way interaction terms for group × time × number of children, with reference categories of control group, baseline (T0), and 1-child families.

b The *P* value for interaction was obtained from a 2-df joint test of the trial arm × family size × time interaction terms because family size was modeled as a 3-category variable.

#### Secondary Outcomes: Moderator Analysis

We did not detect statistically significant moderation by the number of children for the secondary outcomes. Interaction terms were not statistically significant for attitudes toward corporal punishment, child behavior, parenting practices, parental mental health, parenting stress, or family functioning. Interaction estimates were close to zero, with CIs including null.

MDES estimates indicated limited precision for the interaction tests, with larger detectable effects required for families with 3 or more children than for 2-child families. Therefore, the nonsignificant moderation findings should be interpreted cautiously and considered inconclusive, rather than being taken as evidence of equivalent short-term intervention effects across groups defined by the number of children (Table S4 in Multimedia Appendix 1).

As a sensitivity analysis, the number of children was modeled as a continuous moderator, and models including a group×time×number of children interaction were reestimated (Table S5 in Multimedia Appendix 1). Results were consistent with the main analysis, with no significant interaction observed for any outcome, supporting the robustness of the findings.

#### Subgroup Analysis

In subgroup analyses stratified by number of children, patterns of intervention effects varied across outcome domains (Table 5).

**Table 5.** Subgroup analyses of immediate postintervention effects by number of children (N= 1039).

Outcomes	Number of children					
	1 child (n=253)		2 children (n=702)		3 or more children (n=84)	
	Estimate (95% CI)	*P* value	Estimate (95% CI)	*P* value	Estimate (95% CI)	*P* value
Primary outcomes						
Early learning and stimulation: total	1.396 (–1.808 to 4.601)	.39	1.984 (0.118 to 3.850)	.04	2.477 (–2.889 to 7.842)	.37
Caregiver-perpetrated violence: total	–0.124 (–0.481 to 0.232)	.49	–0.117 (–0.342 to 0.108)	.31	–0.293 (–1.083 to 0.497)	.47
Caregiver-perpetrated violence: physical	0.805 (0.482 to 1.343)	.40	0.715 (0.516 to 0.990)	.04	0.494 (0.152 to 1.603)	.24
Caregiver-perpetrated violence: emotional	0.928 (0.649 to 1.328)	.68	0.993 (0.798 to 1.235)	.95	0.93 (0.382 to 2.262)	.87
Secondary outcomes						
Attitude toward corporal punishment	–0.458 (–0.882 to –0.033)	.04	–0.249 (–0.531 to 0.032)	.08	–0.306 (–1.287 to 0.674)	.54
Child behavior: total	–0.173 (–1.569 to 1.224)	.81	–0.587 (–1.362 to 0.188)	.14	–2.473 (–4.356 to –0.590)	.01
Child behavior: internalizing	–0.273 (–1.075 to 0.530)	.51	–0.624 (–1.097 to –0.152)	.01	–1.309 (–2.515 to –0.103)	.03
Child behavior: externalizing	0.095 (–0.817 to 1.008)	.84	0.038 (–0.498 to 0.574)	.89	–1.046 (–2.395 to 0.304)	.13
Child behavior: emotional problem	–0.404 (–0.897 to 0.089)	.11	–0.39 (–0.695 to –0.085)	.01	–0.624 (–1.494 to 0.246)	.16
Child behavior: conduct problem	–0.172 (–0.639 to 0.296)	.47	–0.088 (–0.382 to 0.206)	.56	–0.67 (–1.548 to 0.209)	.14
Child behavior: hyperactivity	0.272 (–0.414 to 0.958)	.44	0.136 (–0.286 to 0.558)	.53	–0.403 (–1.497 to 0.691)	.47
Child behavior: peer problem	0.131 (–0.424 to 0.685)	.64	–0.232 (–0.553 to 0.088)	.16	–0.696 (–1.555 to 0.164)	.11
Child behavior: prosocial behavior	–0.409 (–1.087 to 0.270)	.24	–0.156 (–0.532 to 0.220)	.42	–0.75 (–1.639 to 0.139)	.09
Parenting practices: total	2.205 (–0.674 to 5.085)	.13	2.237 (0.627 to 3.847)	.006	–0.265 (–5.591 to 5.062)	.92
Parenting practices: positive parenting	1.568 (0.188 to 2.948)	.03	0.872 (0.081 to 1.663)	.03	0.662 (–1.939 to 3.263)	.62
Parenting practices: parental involvement	0.635 (–1.114 to 2.384)	.48	1.364 (0.313 to 2.415)	.01	–0.892 (–4.451 to 2.667)	.62
Parental mental health: total	–0.995 (–2.352 to 0.362)	.15	–1.16 (–2.286 to –0.033)	.04	–0.677 (–3.726 to 2.372)	.66
Parental mental health: depression	–0.208 (–1.016 to 0.601)	.62	–0.125 (–0.829 to 0.579)	.73	0.099 (–1.853 to 2.050)	.92
Parental mental health: anxiety	–0.782 (–1.654 to 0.091)	.08	–1.027 (–1.606 to –0.448)	.001	–0.801 (–2.480 to 0.878)	.35
Parenting stress	–2.025 (–4.754 to 0.703)	.15	–0.707 (–2.160 to 0.746)	.34	–4.012 (–8.729 to 0.706)	.09
Family functioning	0.812 (0.005 to 1.619)	.05	0.853 (0.287 to 1.420)	.003	–0.833 (–2.549 to 0.883)	.34

For the primary outcomes, a significant improvement in early learning and stimulation was observed only among families with 2 children (b=1.984, 95% CI 0.118-3.850; *P*=.04). Caregiver-perpetrated violence did not change significantly in any subgroup for total or emotional violence, but physical violence decreased among families with 2 children (IRR=0.715, 95% CI 0.516-0.990; *P*=.04).

For the secondary outcomes, among 1-child families, the intervention was associated with lower endorsement of corporal punishment (b=–0.458, 95% CI –0.882 to –0.033; *P*=.04), higher positive parenting (b=1.568, 95% CI 0.188-2.948; *P*=.03), and improved family functioning (b=0.812, 95% CI 0.005-1.619; *P*=.049).

Among families with 2 children, the intervention was associated with fewer internalizing behaviors (b=–0.624, 95% CI –1.097 to -0.152; *P*=.01) and fewer emotional problems (b=–0.390, 95% CI –0.695 to –0.085; *P*=.01), as well as more proactive parenting practices (b=2.237, 95% CI 0.627-3.847; *P*=.01), greater parental involvement (b=1.364, 95% CI 0.313-2.415; *P*=.01), more positive parenting (b=0.872, 95% CI 0.081-1.663; *P*=.03), fewer total parental mental health symptoms (b=–1.160, 95% CI –2.286 to –0.033; *P*=.04), lower anxiety levels (b=–1.027, 95% CI –1.606 to –0.448; *P*=.001), and improved family functioning (b=0.853, 95% CI 0.287-1.420; *P*=.003).

Among families with 3 or more children, the intervention was associated with fewer total child behavior problems (b=–2.473, 95% CI –4.356 to –0.590; *P*=.01) and fewer internalizing behaviors (b=–1.309, 95% CI –2.515 to –0.103; *P*=.03).

### Differences in Postintervention Trajectories

#### Overview

Distinct from the immediate between-group moderation analyses, the trajectory models examined heterogeneity in within-intervention-group change over time.

Table S6 in Multimedia Appendix 1 summarizes baseline characteristics by number of children in the intervention group (N=247). Of these, 60 (24.29%) families had 1 child, 177 (71.66%) had 2 children, and 10 (4.05%) had 3 or more children. Most caregivers were married (242/247, 97.98%), female (179/247, 72.47%), and living in urban areas (174/247, 70.45%); 57.49% (142/247) of children were boys and 22.67% (56/247) had functional difficulties. Differences by number of children were observed for caregiver age (*P*<.001), child age (*P*=.02), and employment status (*P*<.001), as well as for early learning and stimulation (*P*=.049) and parental involvement (*P*=.04).

In adjusted baseline models restricted to the intervention group (Table S7 in Multimedia Appendix 1), no differences by number of children were observed for the primary outcomes. For the secondary outcomes, compared with 1-child families, 2-child families had more parental mental health concerns (b=1.507, 95% CI 0.299-2.716; *P*=.02), more depressive symptoms (b=0.741, 95% CI 0.121-1.360; *P*=.02), and higher anxiety levels (b=0.767, 95% CI 0.072-1.461; *P*=.03), as well as fewer proactive parenting practices (b=−1.990, 95% CI −3.856 to −0.124; *P*=.04) and less parental involvement (b=−1.935, 95% CI −3.094 to −0.776; *P*=.004). They also showed greater endorsement of corporal punishment (b=0.400, 95% CI 0.031-0.769; *P*=.04) and higher parenting stress (b=2.458, 95% CI 0.295-4.621; *P*=.03).

#### Significance of Interaction

For the primary outcomes, significant time×number of children interactions were observed for early learning and stimulation and caregiver-perpetrated violence (Table 6).

For early learning and stimulation, compared with 1-child families, 2-child families showed greater improvement at T2 (b=3.966, 95% CI 1.468-6.463; *P*=.002), with a similar pattern observed in families with 3 or more children (b=5.749, 95% CI 0.536-10.962; *P*=.03).

**Table 6.** Differences in postintervention trajectories for primary outcomes by number of children within the intervention group (N=806)^a^.

Primary outcomes and time×number of children (Ref. T0×1 child)	Estimate (95% CI)	*P* value
Early learning and stimulation		
T1×2 children	1.836 (−0.688 to 4.360)	.15
T1×≥3 children	4.528 (−0.366 to 9.423)	.07
T2×2 children	3.966 (1.468 to 6.463)	.002
T2×≥3 children	5.749 (0.536 to 10.962)	.03
T3×2 children	2.598 (−0.457 to 5.653)	.09
T3×≥3 children	4.110 (−0.662 to 8.881)	.09
Caregiver-perpetrated violence: total		
T1×2 children	−0.115 (−0.367 to 0.137)	.37
T1×≥3 children	−0.169 (−0.932 to 0.595)	.67
T2×2 children	−0.294 (−0.553 to −0.036)	.03
T2×≥3 children	0.057 (−0.528 to 0.642)	.85
T3×2 children	−0.074 (−0.410 to 0.261)	.66
T3×≥3 children	−0.719 (−1.447 to 0.010)	.05
Caregiver-perpetrated violence: physical		
T1×2 children	0.905 (0.589 to 1.392)	.65
T1×≥3 children	0.975 (0.315 to 3.023)	.97
T2×2 children	0.983 (0.642 to 1.506)	.94
T2×≥3 children	1.536 (0.845 to 2.794)	.16
T3×2 children	0.732 (0.408 to 1.313)	.30
T3×≥3 children	0.452 (0.137 to 1.495)	.19
Caregiver-perpetrated violence: emotional		
T1×2 children	0.900 (0.710 to 1.141)	.38
T1×≥3 children	0.957 (0.367 to 2.494)	.93
T2×2 children	0.672 (0.524 to 0.861)	.002
T2×≥3 children	0.952 (0.602 to 1.506)	.83
T3×2 children	0.966 (0.706 to 1.321)	.83
T3×≥3 children	0.517 (0.281 to 0.952)	.03

a All models were adjusted for caregiver age, child age, marital status, caregiver gender, child gender, educational attainment, ethnicity, hukou status, employment status, and child disability. Total maltreatment was estimated using mixed-effects negative binomial regression, whereas physical abuse and emotional abuse were estimated using mixed-effects Poisson regression and are reported as incidence rate ratios. Other outcomes were estimated using linear mixed-effects regression.

For caregiver-perpetrated violence, 2-child families showed greater reductions in total violence at T2 (b=–0.294, 95% CI –0.553 to –0.036; *P*=.03) and in emotional violence at T2 (IRR=0.672, 95% CI 0.524-0.861; *P*=.002). Families with 3 or more children showed greater reductions in emotional violence at T3 (IRR=0.517, 95% CI 0.281-0.952; *P*=.03) than 1-child families. No significant interaction was observed for physical violence.

For the secondary outcomes, significant interactions were concentrated in child behavior and selected parenting domains (Table S8 in Multimedia Appendix 1).

Compared with 1-child families, families with 3 or more children showed greater reductions in overall child behavior at T1 (b=–3.076, 95% CI –4.990 to –1.162; *P*=.002) and T3 (b=–3.549, 95% CI –6.224 to –0.874; *P*=.009). Similar patterns were observed for internalizing behavior at T1 (b=–1.368, 95% CI–2.108 to –0.628; *P*<.001) and for externalizing behavior at both T1 (b=–1.693, 95% CI –3.192 to –0.193; *P*=.03) and T3 (b=–2.535, 95% CI –4.428 to –0.642; *P*=.009). Additional reductions were observed for conduct problems at T1 (b=–0.807, 95% CI –1.592 to –0.023; *P*=.04), hyperactivity at T3 (b=–2.155, 95% CI –3.641 to –0.669; *P*=.004), and peer problems at T1 (b=–1.139, 95% CI –1.999 to –0.279; *P*=.009).

Among 2-child families, significant interaction effects were observed for overall child behavior at T3 (b=–1.329, 95% CI –2.561 to –0.097; *P*=.03) and hyperactivity at T3 (b=–0.722, 95% CI –1.378 to –0.066; *P*=.03). Improvements were also observed in parental outcomes at T2, including greater parental involvement (b=1.896, 95% CI 0.062-3.731; *P*=.04), fewer overall parental mental health concerns (b=–1.218, 95% CI –2.359 to –0.077; *P*=.04), fewer depressive symptoms (b=–0.838, 95% CI –1.540 to –0.136; *P*=.02), and lower parenting stress (b=–3.551, 95% CI –6.447 to –0.655; *P*=.02). No significant time × number of children interactions were observed for other outcomes.

Furthermore, the longitudinal intervention trajectories remained robust after additionally adjusting for grandparent caregiving (Tables S9 and S10 in Multimedia Appendix 1).

MDES estimates also suggested lower precision for trajectory interactions involving families with 3 or more children, indicating that subgroup-specific patterns in this group should be interpreted with caution (Tables S11 and S12 in Multimedia Appendix 1).

As additional sensitivity analyses, we reestimated unadjusted longitudinal models including time, number of children, and their interaction (Tables S13 and S14 in Multimedia Appendix 1). Results were consistent with the main analysis. Findings for parental and child outcomes were largely unchanged, although some subgroup-specific interaction terms varied in magnitude and statistical significance across model specifications. Additionally, to address potential bias arising from missing data, we performed MICE with 30 imputed datasets to reestimate the fully adjusted models, increasing the analytic sample size from 494 to 541. Results from both the unadjusted and the multiple imputation models were consistent with the main analysis. Overall, the sensitivity analyses supported the robustness of the main findings (Tables S15 and S16 in Multimedia Appendix 1).

#### Estimated Marginal Means

**Figure 1.**
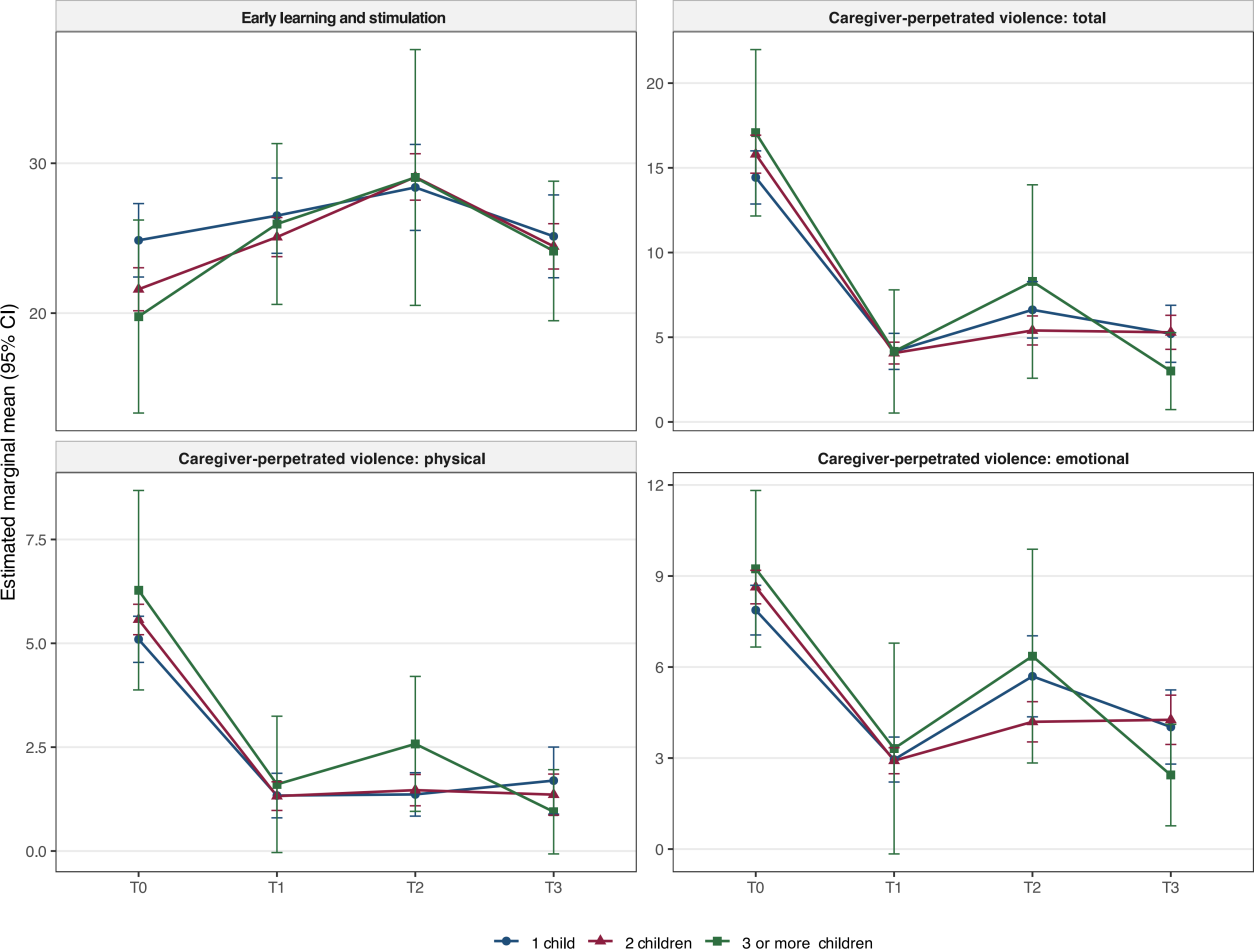
Estimated marginal means for postintervention trajectories by number of children within the intervention group for primary outcomes.

Estimated marginal means showed distinct adjusted trajectories across outcome domains and number of children (Table S17 in Multimedia Appendix 1). Figure 1 visualizes the trajectories.

For the primary outcomes, early learning and stimulation increased from baseline to postintervention, peaked at T2, and declined at T3 across all groups (1-child: 24.86, 26.51, 28.39, and 25.13; 2-child: 21.59, 25.07, 29.09, and 24.46; 3-or-more-child: 19.77, 25.95, 29.05, and 24.15).

Caregiver-perpetrated violence showed the opposite pattern, with substantial reductions immediately after the intervention followed by partial rebound at later follow-up. For total maltreatment, values declined from 14.43 to 4.16 in 1-child families, from 15.81 to 4.06 in 2-child families, and from 17.07 to 4.16 in families with 3 or more children at postintervention; by T3, these values were 5.20, 5.29, and 3.00, respectively.

**Figure 2.**
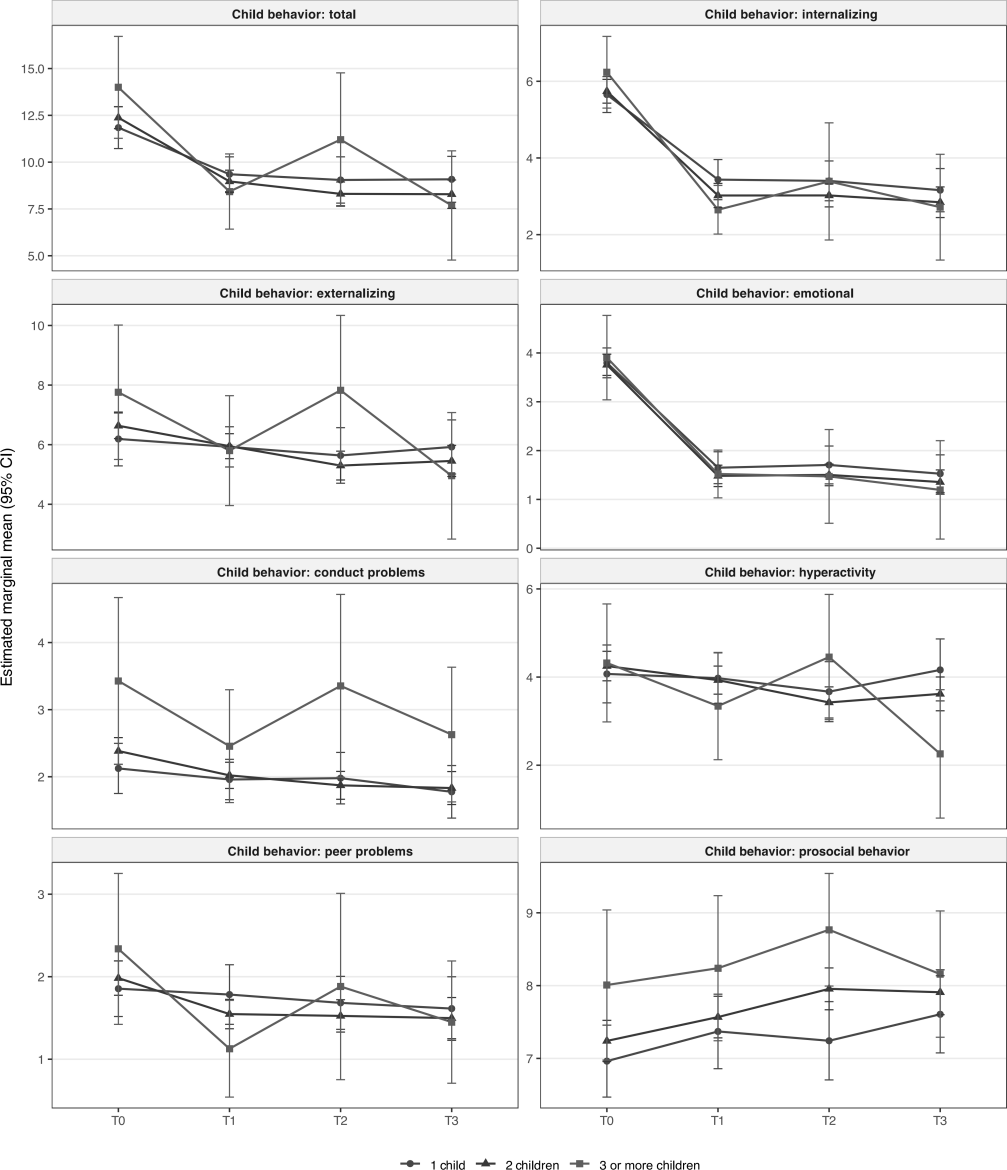
Estimated marginal means for postintervention trajectories by number of children within the intervention group for secondary outcomes: children’s behavior outcomes.

**Figure 3.**
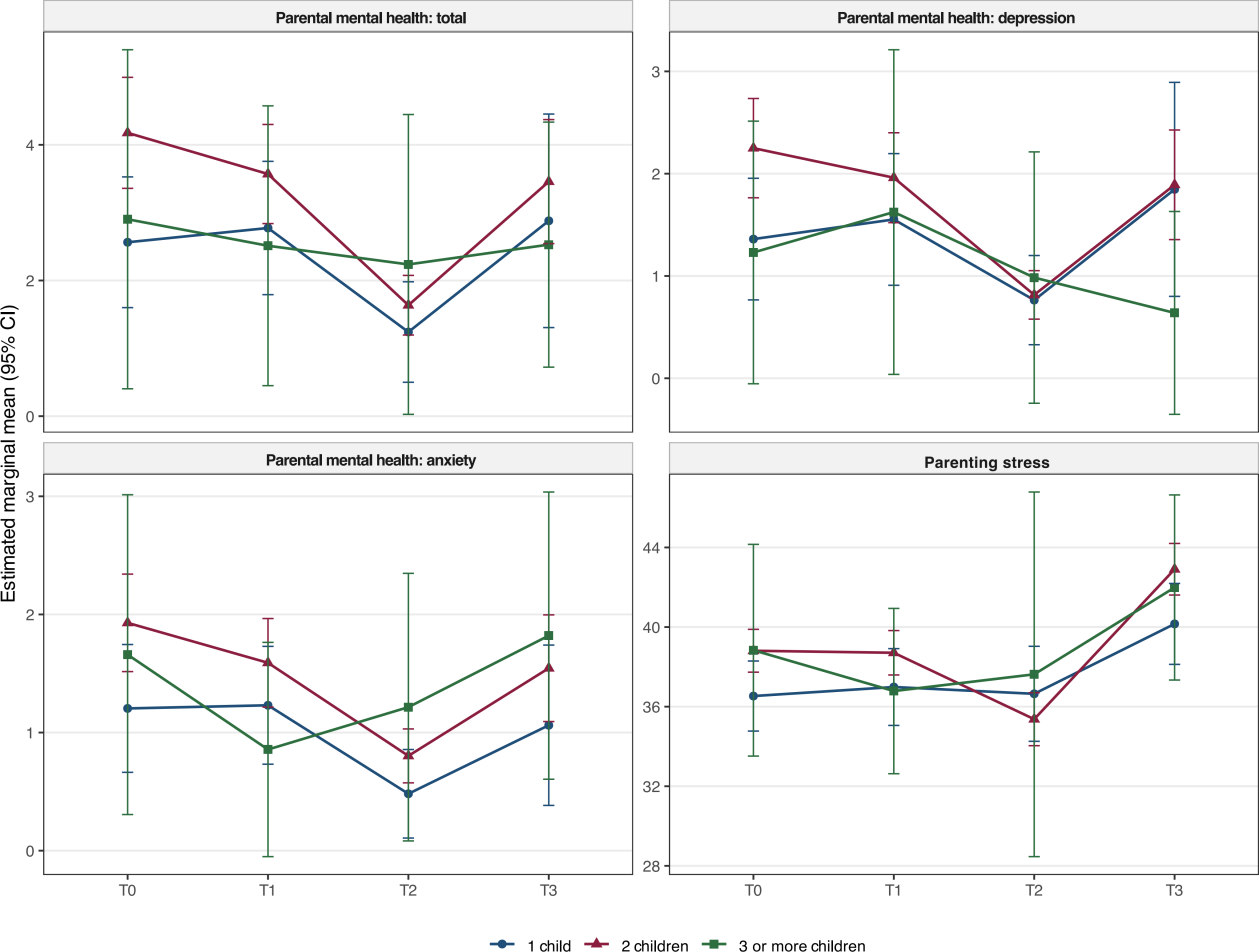
Estimated marginal means for postintervention trajectories by number of children within the intervention group for secondary outcomes: parental mental health and stress outcomes.

**Figure 4.**
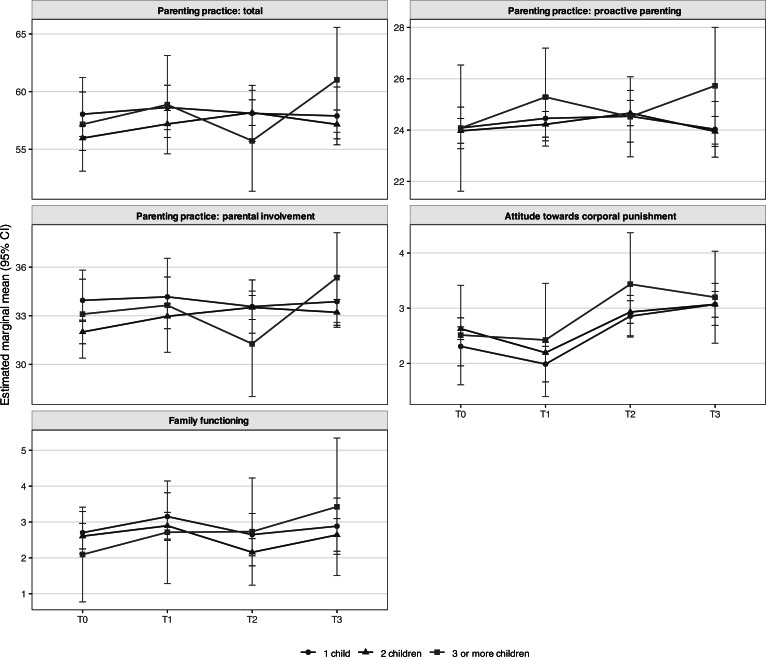
Estimated marginal means for postintervention trajectories by number of children within the intervention group for secondary outcomes: parenting and family process outcomes-proactive parenting practices, attitude toward corporal punishment, and family functioning.

For the secondary outcomes, adjusted trajectories varied across domains (Figures 2-4). Parental mental health concerns decreased to their lowest levels at T2 and increased again by T3, particularly among 1- and 2-child families. Parenting stress showed minimal change immediately after the intervention, decreased at T2 among 2-child families, and was highest at T3 across all groups (40.16, 42.90, and 41.99 for 1-child, 2-child, and 3-or-more-child families, respectively).

Child behavior declined over follow-up, with the most consistent reductions observed among 2-child families (12.38 at T0, 8.97 at T1, 8.31 at T2, and 8.29 at T3). In contrast, trajectories among families with 3 or more children were more variable (14.00, 8.43, 11.20, and 7.69, respectively).

By contrast, proactive parenting practices, family functioning, and attitude toward corporal punishment showed more modest fluctuations over time, with no consistent pattern across groups.

## Discussion

### Main Findings

This study examined whether the number of children is associated with baseline differences and heterogeneous responses to a universal digital-human parenting intervention delivered in a preschool setting.

First, family size was associated with systematic baseline differences: families with more children reported lower levels of early learning and stimulation and positive parenting, alongside higher parenting stress and greater endorsement of corporal punishment, while child behavioral outcomes and caregiver-perpetrated violence were broadly comparable.

Second, family size did not significantly moderate immediate postintervention effects. The program showed similar short-term effectiveness across 1-child, 2-child, and 3-or-more-child families.

Third, differences emerged in postintervention trajectories. Although all groups improved, patterns of change varied over time: 2-child families showed the most consistent improvements, 3-or-more-child families showed larger but more variable gains in some domains, and 1-child families showed more limited changes.

### Number of Children as Constraint and Amplification

The present findings can be interpreted by viewing the number of children as a multidimensional construct that simultaneously imposes constraints and creates opportunities for adaptation within family systems, rather than a purely demographic characteristic [30].

From a constraint perspective, the baseline differences observed are consistent with the resource dilution framework, which posits that parental resources are finite and must be distributed across children [15,16,18]. As family size increases, reduced per-child investment may contribute to lower levels of early learning and stimulation and positive parenting, as well as higher parenting stress. Early learning and stimulation may be particularly sensitive to family size because many of its core activities, including reading, playing, and guided interaction, rely on sustained one-to-one caregiver-child engagement. Such activities are likely to be more affected by competing demands on caregiver time and attention than broader parenting practices. This aligns with evidence that multichild families face greater role strain and competing demands [25,31] and that parenting stress may undermine parenting quality and parent-child interactions [21]. However, for caregiver-perpetrated violence, the interpretation may be more complex. Although caregiver age was adjusted for in the models, the older age profile of caregivers in larger families may be associated with generational differences in parenting norms, including attitudes toward physical discipline. Baseline differences in caregiver-perpetrated violence may therefore reflect not only resource-related pressures associated with raising multiple children, but also cohort-related variation in disciplinary beliefs and practices.

However, the trajectory findings suggest a more nuanced pattern than predicted by deficit-oriented accounts of family size. The stronger improvements observed in some outcomes among multichild families are consistent with an amplification pathway. Family systems theory emphasizes that changes in parenting behaviors may generalize across children within the household, with intervention-related skills extending beyond the initially targeted child [32].

Multichild families may provide more opportunities to apply and reinforce newly acquired skills across children and contexts. Sibling interactions may also create natural settings for social learning, potentially facilitating broader changes in family functioning [22]. These processes may help explain why families with more children, despite lower baseline levels, showed larger gains in some domains.

These dual pathways help reconcile the contrast between baseline disadvantage and postintervention gains. While greater caregiving demands may constrain initial conditions and implementation capacity, they may also support the diffusion of intervention effects within the family system. This is consistent with evidence suggesting that larger families may, under some conditions, function as “micro-systems” that facilitate relational adaptation and resilience [24].

### Immediate Moderation and Longer-Term Trajectory Heterogeneity

Despite differences in baseline conditions and postintervention trajectories, this study did not detect statistically significant moderation by the number of children at the immediate postintervention. This pattern is consistent with prior research showing that well-designed parenting interventions can produce broadly similar short-term changes across diverse family contexts, including those facing structural disadvantages [4,6]. However, given the small number of families with 3 or more children and the wide CIs surrounding several interaction estimates, these findings should be interpreted as inconclusive rather than as evidence of equivalent short-term effects across groups defined by number of children.

One explanation is that differences related to caregiving demands may not manifest immediately but emerge over time. Parenting interventions often target proximal behaviors that can be modified relatively quickly, leading to similar short-term gains across families. However, differences in implementation burden and resource constraints may become more consequential for sustaining these gains, resulting in divergence in longer-term trajectories. Methodologically, these findings address a different question from the moderation analysis. Whereas the T0-T1 analyses estimated between-group differences in short-term intervention effects, the T2-T3 analyses examined patterns of change within the intervention group after the waitlist control group had crossed over. These trajectories may reflect delayed or nonlinear intervention-related change, but they may also be influenced by child maturation and broader environmental factors. This interpretation is consistent with evidence that intervention effects may follow nonlinear patterns over time, with initial improvements followed by divergence, and that trajectories may vary across family contexts [33], and it aligns with the heterogeneous trajectories observed at 6- and 12-month follow-ups.

A second explanation relates to statistical power. The relatively small number of families with 3 or more children may have limited the ability to detect moderation effects, particularly in interaction models. As a result, the absence of significant moderation should be interpreted with caution.

Finally, these findings highlight a distinction between average effects and patterns of change. While intervention effects may appear homogeneous at the immediate postintervention time point, heterogeneity may become evident when trajectories are examined over time, underscoring the importance of longitudinal and context-sensitive approaches to evaluation.

### Implications of Digital-Human Intervention Delivery

The role of family size observed in this study may be particularly salient in the context of digital-human parenting interventions. Compared to traditional face-to-face programs, these interventions are designed to be scalable and accessible, relying more heavily on self-directed engagement with structured content and less intensive human support [11].

This shift may increase the extent to which family context shapes intervention processes. Caregivers managing multiple children may face greater challenges in consistently engaging with materials and integrating recommended practices into daily routines, as competing demands and resource constraints influence the uptake and maintenance of new parenting strategies [18,21].

At the same time, the flexibility of digital-human interventions may enable adaptation to diverse family contexts. Self-paced delivery and repeated access to materials may facilitate practice across caregiving situations, while group-based components may support peer learning and engagement.

### The Chinese Context and Broader Relevance

The findings should be interpreted within the broader demographic and sociocultural context of contemporary China, where rapid fertility policy changes have been accompanied by shifts in family structures and caregiving arrangements. Following decades of the 1-child policy, recent policy relaxations have led to the reemergence of multichild families within a relatively short period. However, this transition has occurred alongside a generational discontinuity in caregiving experience, as many current parents did not grow up in multichild households and therefore may have limited intergenerational guidance for managing sibling dynamics [25,26].

In this context, caregiving demands may be particularly salient. These demands are further shaped by broader social pressures, including high educational expectations and intensive parenting norms, which may increase the challenges of raising multiple children [34,35].

At the same time, the Chinese context helps illustrate how both constraints and adaptive processes may coexist. Limited experience with multichild parenting and uneven access to formal support may heighten resource constraints, particularly in the early stages of adjustment. Conversely, strong family interdependence and the involvement of extended kin, such as grandparents, may facilitate within-family learning and adaptation over time [36,37].

More broadly, these findings have implications beyond the Chinese context. As family structures evolve in many settings, including through declining fertility, delayed childbearing, and changing caregiving arrangements, variation in family size may interact with social and institutional contexts to shape how interventions are implemented and sustained. Understanding these contextual dynamics is important for designing parenting interventions that are both scalable and responsive to diverse family needs.

### Implications for Research, Policy, and Practice

First, this study highlights the importance of moving beyond treating family structure as a control variable toward recognizing it as a meaningful dimension shaping the intervention process. The observed differences in baseline conditions and postintervention trajectories underscore the need for more context-sensitive research approaches that integrate heterogeneity with longitudinal dynamics.

Second, while parenting interventions may appear broadly effective in the short-term across family types, sustaining gains may require additional support, particularly for families facing higher caregiving demands. Incorporating strategies that reinforce long-term practice of skills—such as booster sessions, ongoing digital reminders, or structured follow-up support—may help maintain intervention effects over time.

Third, multichild families should not be viewed solely as resource-constrained. They may also provide opportunities for amplifying intervention impact, as parenting strategies can be applied across children. Designing interventions that leverage these within-family dynamics (such as promoting positive sibling interactions) may enhance overall impacts.

Fourth, the findings have particular implications for scalable digital-human interventions, where implementation relies heavily on caregivers. Differentiated pacing, targeted support for families with multiple children, or guidance on managing competing caregiving demands may help reduce implementation burden while preserving scalability.

Finally, in rapidly changing contexts such as China, policies and services need to better align with evolving family structures. Policies aimed at creating family-friendly environments may benefit from focusing not only on fertility levels but also on strengthening support systems that enable families to sustain positive parenting practices over time. Targeted support for multichild families—through parenting resources, community-based support, and integration with early childhood services—may help mitigate caregiving challenges and improve the sustainability of intervention benefits.

### Strengths and Limitations

This study has several limitations. First, the relatively small number of families with 3 or more children, particularly within the intervention group, limited statistical power for the moderation and trajectory analyses. This increased the risk of type II error and reduced the precision of interaction estimates. Because only 10 families with 3 or more children contributed to the intervention-group trajectory analyses, the observed changes in this subgroup may be sensitive to individual cases and should not be interpreted as representative or confirmatory subgroup trends. Accordingly, these findings are best viewed as exploratory and hypothesis-generating. Second, all outcomes were based on caregiver self-report and may therefore be subject to reporting bias, particularly for sensitive behaviors such as violent parenting. For example, reductions in caregiver-perpetrated violence at T1 may partly reflect social desirability bias, as caregivers may have felt expected to report improvements in parenting behaviors after receiving the intervention. Third, due to the waitlist design, longer-term follow-up data were available only for the intervention group, limiting causal inference on sustained effects. Fourth, the number of children captures only one dimension of family size and caregiving complexity; other contextual factors, such as coparenting dynamics, may also shape intervention processes and should be examined in future research. This study also has several strengths. It integrates baseline differences, immediate postintervention effectiveness, and longitudinal trajectories within a single analytical framework, thereby providing a comprehensive assessment of intervention heterogeneity. The use of a cluster randomized controlled trial embedded in a real-world preschool system enhances both internal and external validity. In addition, the use of a standardized digital-human intervention supports consistency in delivery while maintaining contextual relevance.

### Conclusion

This study provides evidence that the number of children is associated with variation in parenting intervention processes across baseline conditions, short-term effects, and longer-term trajectories. While intervention effectiveness was similar across family types in the short term, differences emerged in the sustainability of outcomes over time.

These findings suggest that caregiving demands may be less relevant for whether intervention effects occur than for how they are sustained and evolve over time. They highlight the importance of moving beyond average treatment effects to consider family context and longitudinal dynamics when understanding intervention responses.

By examining family size as a source of variation in caregiving contexts rather than solely as a control variable, this study contributes to a more nuanced understanding of heterogeneity in parenting intervention outcomes. It further integrates baseline differences, short-term effectiveness, and longer-term trajectories within a single analytical framework. Situated in contemporary China, where family structures and caregiving demands are rapidly changing, the findings also have practical implications for the design of parenting interventions, particularly the need for sustained and context-responsive support to promote long-term gains.

## Supplementary material

10.2196/101388 Multimedia Appendix 1 Supplementary methods, baseline characteristics, and additional analyses by number of children.

10.2196/101388 Checklist 1 CONSORT checklist.

## Abbreviations

APQ: Alabama Parenting Questionnaire
DASS-21: Depression Anxiety Stress Scales-21
ICAST: International Society for the Prevention of Child Abuse and Neglect Child Abuse Screening Tool
IRR: incidence rate ratio
ISPCAN: International Society for the Prevention of Child Abuse and Neglect
MCAR: missing completely at random
MDES: minimum detectable effect size
MICE: multiple imputation by chained equations
MICS: Multiple Indicator Cluster Surveys
PLH: Parenting for Lifelong Health
PSS: Parental Stress Scale
SDQ: Strengths and Difficulties Questionnaire
