# Supplementary material for: Family Size and Longitudinal Outcomes of a Digital-Human Parenting Intervention in Chinese Preschool Families: Secondary Analysis
Source: J Med Internet Res. 2026 Jul 10;28:e101388. doi: 10.2196/101388 (PMC13354134; doi:10.2196/101388)
Supplement: Multimedia Appendix 1 [file jmir-v28-e101388-s001.docx]

**Supplement to**: Fang, Z., He, X., Shi, X., Ruan, R., Lachman, J.M. (2026). Family Size and Longitudinal Outcomes of a Digital–Human Parenting Intervention in Chinese Preschool Families: Secondary Analysis

**Content**

[1. Participant Flowchart 3](#_Toc1598022540)

[2. Measures and Psychometric Properties 3](#_Toc1534340398)

[Primary outcomes 4](#_Toc675436010)

[3. Statistical Analysis (detailed) 5](#_Toc454462028)

[4. Supplement Tables and Figures 8](#_Toc1691455864)

[Table S1 Distribution Check for Caregiver-Perpetrated Violence 8](#_Toc2057670909)

[*Table S2 Distribution of complete-case baseline analytic status by trial arm and number of children* 9](#_Toc198316275)

[*Table S3* The baseline association between number of children and outcomes in full sample, adjusted for grandparent caregiving (N=494) 10](#_Toc207013781)

[Table S4 Approximate MDEs for key interaction tests: immediate post-intervention moderation for primary outcomes 12](#_Toc357109127)

[Table S5 Sensitivity analysis: moderation of immediate post-intervention effects by number of children for outcomes (number of children as a continuous variable) (N= 1039) 13](#_Toc1863787771)

[Table S6 Baseline outcome measures by number of children in intervention group (N=247) 15](#_Toc1484592055)

[Table S7 The baseline association between secondary outcomes in intervention group (N=247) 17](#_Toc2073657539)

[Table S8 Differences in post-intervention trajectories for secondary outcomes by number of children within the intervention group (N = 806) 19](#_Toc450150810)

[Table S9 Differences in post-intervention trajectories for primary outcomes by number of children within the intervention group,adjusted for grandparent caregiving (N = 806) 21](#_Toc99775289)

[Table S10 Differences in post-intervention trajectories for secondary outcomes by number of children within the intervention group, adjusted for grandparent caregiving (N = 806) 23](#_Toc1886037563)

[*Table S11 Approximate MDEs for key interaction tests: intervention-group trajectories for primary outcomes* 26](#_Toc1774691621)

[*Table S12 Approximate MDEs for key interaction tests: intervention-group trajectories for secondary interactions explicitly interpreted in the Results* 28](#_Toc841700964)

[Table S13 Sensitivity analysis: differences in post-intervention trajectories for primary outcomes by number of children within the intervention group with unadjusting sociodemographic covariates (N = 885) 29](#_Toc993319159)

[Table S14 Sensitivity analysis: differences in post-intervention trajectories for secondary outcomes by number of children within the intervention group with unadjusting sociodemographic covariates (N = 885) 30](#_Toc177233535)

[Table S15 Sensitivity analysis: differences in post-intervention trajectories for primary outcomes by number of children within the intervention group after MICE (N = 885) 33](#_Toc204204356)

[Table S16 Sensitivity analysis: differences in post-intervention trajectories for secondary outcomes by number of children within the intervention group after MICE (N = 885) 34](#_Toc383743386)

[Table S17 Estimated Marginal Means for Post-Intervention Trajectories by Family Size Within the Intervention Group (N = 806) 37](#_Toc681696561)

[5. References 39](#_Toc448843982)

# Participant Flowchart


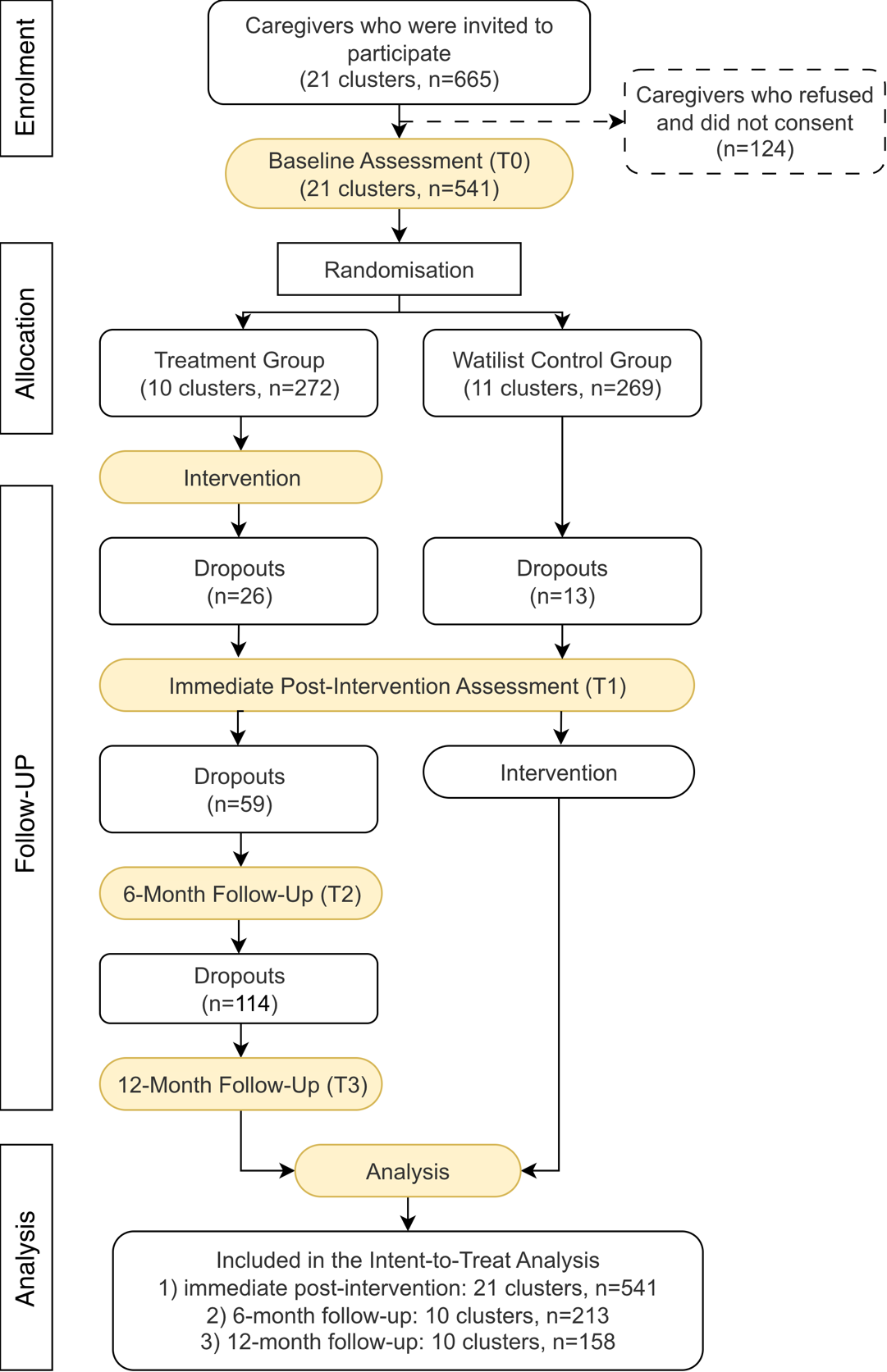


**Note**. At each follow-up, all participants originally allocated to the intervention group were invited to complete the assessment, regardless of prior participation. Dropout numbers were specific to each phase and not cumulative from earlier time points.

# Measures and Psychometric Properties

**Primary outcomes**

Early learning and stimulation

Early learning and stimulation was assessed using six items from the Multiple Indicator Cluster Surveys (MICS) [1], capturing the frequency of caregiver engagement in developmentally stimulating activities (e.g., reading, playing, and interactive learning). Scores ranged from 0 to 48, with higher scores indicating more frequent stimulation. In the present study, early learning and stimulation was analyzed as a total score. These items are embedded within the MICS Early Childhood Development framework (ECDI2030), which has been validated in Chinese populations and demonstrates acceptable internal consistency (α ≈ 0.73) [2].

Caregiver-perpetrated violence

Caregiver-perpetrated violence was measured using nine items from the ISPCAN Child Abuse Screening Tool (ICAST) [3], assessing the frequency of physical and emotional disciplinary practices. Higher scores indicate more frequent use of violent discipline. For analysis, we examined caregiver-perpetrated violence as a total score, physical violence subscale, and emotional violence subscale. The instrument has been validated in mainland Chinese samples, with confirmatory factor analysis supporting its multidimensional structure and internal consistency coefficients ranging from 0.60 to 0.87 across subscales [4].

**Secondary outcomes**

Child behavior problems

Child behavior problems were measured using the Strengths and Difficulties Questionnaire (SDQ) [5], including total difficulties and subscales, with higher total score indicating more behavior challenges and higher prosocial score indicating better prosocial skills. The analyses included the total difficulties score, internalizing behavior, externalizing behavior, emotional problems, conduct problems, hyperactivity, peer problems, and prosocial behaviors. SDQ has been validated in large Chinese samples, demonstrating acceptable reliability (α ≈ 0.69–0.73) and established construct validity [6,7].

Parenting practices

Parenting practices were assessed using subscales of the Alabama Parenting Questionnaire (APQ) [8], including positive parenting and parental involvement, with higher score indicating better outcome. Specifically, we analyzed an overall parenting practices score, the positive parenting subscale and the parental involvement subscale. Short-form versions of the APQ have been validated in Chinese samples, showing stable factor structures and acceptable reliability [9].

Parental mental health

Parental mental health was measured using the Depression and Anxiety subscales of the DASS-21 with higher score indicating more mental health symptoms [10,11]. In this study, we used the total DASS score, the depression and anxiety subscale scores. This scale has been extensively validated in Chinese adult populations, demonstrating high internal consistency (α ≈ 0.95) and measurement invariance across gender [12–14].

Parenting stress

Parenting stress was assessed using the Parental Stress Scale (PSS) with higher score indicating more stress [15], which has shown strong reliability in Chinese samples (Cronbach’s α ≈ 0.89) across both clinical and non-clinical populations [16]. Parenting stress was analyzed as a total score.

Family functioning

Family functioning was measured using the Family APGAR scale with higher score indicating better outcomes [17], which has been widely validated in Chinese populations, demonstrating excellent internal consistency (α ≈ 0.88–0.91) and strong convergent validity [17]. In the present study, family functioning was analyzed as a total score.

Attitudes toward corporal punishment

Attitudes toward corporal punishment were assessed using a single item from MICS with higher score indicating more endorsement of corporal punishment [1]. Although measured using a single item, it is part of a widely used international survey framework with established content validity in cross-cultural settings.

# Statistical Analysis (detailed)

Baseline sociodemographic characteristics and baseline outcome measures were summarized for the full sample by number of children. Continuous variables were described using means and standard deviations, and categorical variables were described using frequencies and percentages. Baseline differences in sociodemographic characteristics across family-size groups were examined using one-way analyses of variance or chi-square tests. Associations between number of children and baseline parenting and child outcomes in the full sample were examined using multivariable regression models selected according to the distribution of each outcome. Model type including linear mixed-effects models, Poisson regression models and negative binomial regression models. All regression models adjusted for the same sociodemographic context covariates.

To test whether intervention effects varied by number of children, parenting and child outcomes outcome in the full sample were analyzed using multivariable regression models selected according to the distribution of each outcome. The post-intervention effectiveness analysis followed the intention-to-treat design, with participants analyzed according to their original group assignment. Random intercepts for class (cluster) and family were included to account for clustering at the class level and repeated assessments within families. Models included time, study group, number of children, all constituent two-way interactions, and the group×time×number of children interaction. Subgroup analyses stratified by number of children were conducted to estimate intervention effects within each family-size category, and the group×time×number of children interaction was used as the formal test of whether intervention effects differed by number of children. As a sensitivity analysis, the moderation models were re-estimated with child number treated as a continuous variable.

To examine post-intervention trajectories within the intervention arm, baseline sociodemographic characteristics and baseline outcomes were first summarized for the intervention group by number of children. We then used multivariable regression models to examine associations between number of children and baseline parenting and child outcomes within the intervention group, adjusting for sociodemographic covariates. Separate multivariable mixed-effects regression models were estimated using intervention-group data from T0 to T3, with model type selected according to the distribution of each outcome. Random intercepts for class (cluster) and family were included to account for clustering at the class level and repeated assessments within families. All models adjusted for prespecified baseline sociodemographic covariates and included time, number of children, and the time×number of children interaction. Robust standard errors were used in all models. As a sensitivity analysis, these trajectory models were re-estimated without adjustment for baseline sociodemographic covariates. Estimated marginal means were obtained for all outcomes to present adjusted trajectories over time by number of children.

Participants with missing data on variables required for a given model were excluded from that specific analysis. This complete-case approach was used because missingness was limited and varied across model-specific covariates, and it allowed each model to be estimated using observed data without imputing values for baseline characteristics or outcome measures. Model-specific sample sizes are reported in the relevant tables. All tests were two-tailed, with statistical significance set at p < 0.05. All analyses were conducted in Stata 17.0 and R.

.

# Supplement Tables and Figures

## Table S1 Distribution Check for Caregiver-Perpetrated Violence

|  | **AD test** | | **Dispersion test** | | | | **Distribution** |
| --- | --- | --- | --- | --- | --- | --- | --- |
| **Caregiver-Perpetrated Violence** | p-value raw | p-value log | ChiSq | Ratio | DF | p-value |  |
| Total | <.001 | <.001 | 1189.16 | 1.15 | 1038.00 | <.001 | Negative Binomial |
| Physical | <.001 | <.001 | 1042.99 | 1.00 | 1038.00 | .451 | Poisson |
| Emotional | <.001 | <.001 | 877.34 | 0.85 | 1038.00 | 1.000 | Poisson |

**Note:**Distribution check was restricted to caregiver-perpetrated violence outcomes because these outcomes were analyzed as count variables. All other outcomes were continuous scale scores and were analyzed using linear models. The dispersion test informed the choice between Poisson and negative binomial models. AD = Anderson–Darling; DF = degrees of freedom

## Table S2 Distribution of complete-case baseline analytic status by trial arm and number of children

***Panel A. By trial arm***

| Trial arm | Included, n (%) | Excluded, n (%) | Total | P value |
| --- | --- | --- | --- | --- |
| Control | 247 (91.82) | 22 (8.18) | 269 | .676 |
| Intervention | 247 (90.81) | 25 (9.19) | 272 |  |
| Total | 494 (91.31) | 47 (8.69) | 541 |  |

***Panel B. By number of children***

| Number of children | Included, n (%) | Excluded, n (%) | Total | P value |
| --- | --- | --- | --- | --- |
| 1 child | 120 (92.31) | 10 (7.69) | 130 | .001 |
| 2 children | 340 (93.41) | 24 (6.59) | 364 |  |
| 3+ children | 34 (75.56) | 11 (24.44) | 45 |  |
| Total | 494 (91.65) | 45 (8.35) | 539 |  |

Note: Percentages are row percentages. Complete-case status refers to inclusion in the baseline analytic sample with non-missing baseline sociodemographic covariates. P values were obtained using Pearson’s chi-square test for trial arm and Fisher’s exact test for number of children. Family-size-specific analyses were based on 539 families with non-missing information on number of children; two families had missing family-size information

## Table S3 The baseline association between number of children and outcomes in full sample, adjusted for grandparent caregiving (N=494)

| **Outcomes** | **Number of children**  **(Ref. 1 child)** | **Estimate** | **P** | **95% CI** |
| --- | --- | --- | --- | --- |
| **Primary outcomes** |  |  |  |  |
| Early Learning and Stimulation | 2 children | -3.591 | .002 | (-5.729, -1.452) |
|  | 3+ children | -6.214 | .009 | (-10.693, -1.734) |
| Caregiver-Perpetrated Violence: Total | 2 children | 0.033 | .465 | (-0.056, 0.123) |
|  | 3+ children | 0.096 | .238 | (-0.064, 0.257) |
| Caregiver-Perpetrated Violence: Physical | 2 children | 1.064 | .287 | (0.949, 1.193) |
|  | 3+ children | 1.018 | .860 | (0.839, 1.234) |
| Caregiver-Perpetrated Violence: Emotional | 2 children | 1.013 | .793 | (0.917, 1.119) |
|  | 3+ children | 1.167 | .129 | (0.956, 1.426) |
| **Secondary outcomes** |  |  |  |  |
| Parental Mental Health: Total | 2 children | 0.568 | .343 | (-0.652, 1.789) |
|  | 3+ children | 0.768 | .395 | (-1.073, 2.609) |
| Parental Mental Health: Depression | 2 children | 0.568 | .028 | (0.067, 1.069) |
|  | 3+ children | 0.366 | .432 | (-0.586, 1.317) |
| Parental Mental Health: Anxiety | 2 children | 0.001 | .999 | (-0.824, 0.826) |
|  | 3+ children | 0.402 | .484 | (-0.772, 1.576) |
| Child Behavior: Total | 2 children | -0.197 | .681 | (-1.184, 0.790) |
|  | 3+ children | 0.125 | .899 | (-1.906, 2.155) |
| Child Behavior: Emotional problem | 2 children | -0.012 | .942 | (-0.349, 0.325) |
|  | 3+ children | 0.003 | .991 | (-0.479, 0.484) |
| Child Behavior: Conduct problem | 2 children | 0.073 | .657 | (-0.266, 0.413) |
|  | 3+ children | 0.370 | .256 | (-0.290, 1.030) |
| Child Behavior: Hyperactivity | 2 children | -0.226 | .445 | (-0.832, 0.379) |
|  | 3+ children | -0.265 | .602 | (-1.308, 0.778) |
| Child Behavior: Peer problem | 2 children | -0.033 | .857 | (-0.407, 0.342) |
|  | 3+ children | 0.017 | .955 | (-0.587, 0.621) |
| Child Behavior: Prosocial behavior | 2 children | 0.093 | .682 | (-0.373, 0.559) |
|  | 3+ children | 0.549 | .130 | (-0.177, 1.275) |
| Child Behavior: Externalizing behavior | 2 children | -0.153 | .692 | (-0.945, 0.640) |
|  | 3+ children | 0.105 | .889 | (-1.452, 1.663) |
| Child Behavior: Internalizing behavior | 2 children | -0.044 | .878 | (-0.642, 0.554) |
|  | 3+ children | 0.019 | .964 | (-0.849, 0.888) |
| Parenting Practices: Total | 2 children | -1.917 | .029 | (-3.618, -0.216) |
|  | 3+ children | -2.085 | .191 | (-5.302, 1.131) |
| Parenting Practices: Positive parenting | 2 children | -0.448 | .283 | (-1.297, 0.400) |
|  | 3+ children | -0.592 | .361 | (-1.913, 0.730) |
| Parenting Practices: Parental involvement | 2 children | -1.469 | .007 | (-2.491, -0.446) |
|  | 3+ children | -1.494 | .242 | (-4.075, 1.088) |
| Attitude towards Corporal Punishment | 2 children | 0.456 | .007 | (0.138, 0.774) |
|  | 3+ children | 0.745 | .028 | (0.088, 1.402) |
| Parenting Stress | 2 children | 2.395 | .012 | (0.586, 4.204) |
|  | 3+ children | 3.928 | .042 | (0.155, 7.701) |
| Family Functioning | 2 children | -0.151 | .504 | (-0.614, 0.312) |
|  | 3+ children | -0.615 | .162 | (-1.500, 0.269) |

Note: One-child families were the reference group. All models were adjusted for caregiver age, child age, marital status, caregiver gender, child gender, educational attainment, ethnicity, hukou status, employment status, child disability, and grandparent caregiving. Estimates for early learning and stimulation and secondary outcomes are regression coefficients. Total caregiver-perpetrated violence was estimated using negative binomial regression and is reported on the log-count scale, whereas physical and emotional caregiver-perpetrated violence were estimated using Poisson regression and are reported as incidence rate ratios (IRRs). CI = confidence interval; IRR = incidence rate ratio.

| Table S4 Approximate MDEs for key interaction tests: immediate post-intervention moderation for primary outcomes | | | | |
| --- | --- | --- | --- | --- |
| Outcome | Interaction term | Estimate | 95% CI | Approximate MDE (80% power) |
| Early Learning and Stimulation | group × time × 2 children | .59 | -3.045 to 4.225 | 5.19 scale points |
| Early Learning and Stimulation | group × time × 3+ children | 1.159 | -5.343 to 7.660 | 9.29 scale points |
| Caregiver-Perpetrated Violence: Total | group × time × 2 children | IRR 1.007 | 0.717 to 1.416 | IRR ≤0.62 or ≥1.63 |
| Caregiver-Perpetrated Violence: Total | group × time × 3+ children | IRR 0.845 | 0.382 to 1.866 | IRR ≤0.32 or ≥3.10 |
| Caregiver-Perpetrated Violence: Physical | group × time × 2 children | IRR 0.889 | 0.531 to 1.486 | IRR ≤0.48 or ≥2.09 |
| Caregiver-Perpetrated Violence: Physical | group × time × 3+ children | IRR 0.614 | 0.170 to 2.221 | IRR ≤0.16 or ≥6.27 |
| Caregiver-Perpetrated Violence: Emotional | group × time × 2 children | IRR 1.069 | 0.776 to 1.474 | IRR ≤0.63 or ≥1.58 |
| Caregiver-Perpetrated Violence: Emotional | group × time × 3+ children | IRR 1.002 | 0.440 to 2.284 | IRR ≤0.31 or ≥3.24 |

Note: MDE = minimum detectable effect size. Approximate MDEs were derived from the standard errors implied by the 95% confidence intervals, assuming a two-sided α of 0.05 and 80% power. For early learning and stimulation, MDEs are expressed as absolute differences in scale points. For caregiver-perpetrated violence outcomes, interaction estimates and detectable thresholds are expressed as incidence rate ratios (IRRs) after transformation from the log-rate scale. Values below and above 1 indicate detectable decreases and increases, respectively. These calculations were used to contextualize statistical precision and should not be interpreted as additional hypothesis tests.

##

## Table S5 Sensitivity analysis: moderation of immediate post-intervention effects by number of children for outcomes (number of children as a continuous variable) (N= 1039)

| Outcomes | Three-way interaction term | Estimate | 95% CI | P for interaction |
| --- | --- | --- | --- | --- |
| **Primary outcomes** |  |  |  |  |
| Early Learning and Stimulation | group×time×number of children | 0.621 | (-2.225, 3.467) | .669 |
| Caregiver-Perpetrated Violence: Total | group×time×number of children | -0.074 | (-0.353, 0.206) | .606 |
| Caregiver-Perpetrated Violence: Physical | group×time×number of children | 0.792 | (0.488, 1.286) | .346 |
| Caregiver-Perpetrated Violence: Emotional | group×time×number of children | 1.017 | (0.765, 1.352) | .907 |
| **Secondary outcomes** |  |  |  |  |
| Attitude towards Corporal Punishment | group×time×number of children | 0.080 | (-0.346, 0.505) | .714 |
| Child Behavior: Total | group×time×number of children | -0.853 | (-2.040, 0.335) | .159 |
| Child Behavior: Internalizing | group×time×number of children | -0.430 | (-1.144, 0.283) | .237 |
| Child Behavior: Externalizing | group×time×number of children | -0.402 | (-1.213, 0.410) | .332 |
| Child Behavior: Emotional problem | group×time×number of children | -0.043 | (-0.501, 0.415) | .853 |
| Child Behavior: Conduct problem | group×time×number of children | -0.138 | (-0.580, 0.304) | .541 |
| Child Behavior: Hyperactivity | group×time×number of children | -0.265 | (-0.896, 0.366) | .411 |
| Child Behavior: Peer problem | group×time×number of children | -0.380 | (-0.868, 0.107) | .126 |
| Child Behavior: Prosocial behavior | group×time×number of children | 0.018 | (-0.556, 0.592) | .951 |
| Parenting Practices: Total | group×time×number of children | -0.686 | (-3.195, 1.823) | .592 |
| Parenting Practices: Positive parenting | group×time×number of children | -0.581 | (-1.803, 0.641) | .352 |
| Parenting Practices: Parental involvement | group×time×number of children | -0.096 | (-1.712, 1.520) | .907 |
| Parental Mental Health: Total | group×time×number of children | 0.009 | (-1.572, 1.589) | .992 |
| Parental Mental Health: Depression | group×time×number of children | 0.091 | (-0.892, 1.074) | .856 |
| Parental Mental Health: Anxiety | group×time×number of children | -0.092 | (-0.947, 0.762) | .833 |
| Parenting Stress | group×time×number of children | -0.146 | (-2.445, 2.154) | .901 |
| Family Functioning | group×time×number of children | -0.610 | (-1.450, 0.229) | .154 |

Note: Number of children was modeled as a continuous variable. The three-way interaction estimate represents the change in the group × time interaction associated with a one-child increase in family size. The control group and baseline (T0) served as the reference categories for the categorical factors. Estimates for continuous outcomes are regression coefficients. The estimate for total caregiver-perpetrated violence is reported on the log-count scale, whereas estimates for physical and emotional caregiver-perpetrated violence are reported as incidence rate ratios (IRRs). CI = confidence interval; IRR = incidence rate ratio.

## Table S6 Baseline sociodemographic characteristics and outcome measures by number of children in intervention group (N=247)

|  |  | Number of children | | |  |
| --- | --- | --- | --- | --- | --- |
| Variables | Total sample (N=247) | 1 child (n=60) | 2 children (n=177) | 3+ children (n=10) | P-value |
| **Sociodemographic context variables** | | | | | |
| Marital status, n (%) |  |  |  |  | .750 |
| Single | 4 (1.62) | 2 (3.33) | 2 (1.13) | 0 (0.00) |  |
| Unmarried but not single | 1 (0.40) | 0 (0.00) | 1 (0.56) | 0 (0.00) |  |
| Married | 242 (97.98) | 58 (96.67) | 174 (98.31) | 10 (100.00) |  |
| Hukou, n (%) |  |  |  |  | .399 |
| Rural | 73 (29.55) | 14 (23.33) | 55 (31.07) | 4 (40.00) |  |
| Urban | 174 (70.45) | 46 (76.67) | 122 (68.93) | 6 (60.00) |  |
| Adult gender, n (%) |  |  |  |  | .786 |
| Male | 68 (27.53) | 18 (30.00) | 48 (27.12) | 2 (20.00) |  |
| Female | 179 (72.47) | 42 (70.00) | 129 (72.88) | 8 (80.00) |  |
| Children gender, n (%) |  |  |  |  | .193 |
| Male | 142 (57.49) | 29 (48.33) | 107 (60.45) | 6 (60.00) |  |
| Female | 105 (42.51) | 31 (51.67) | 70 (39.55) | 4 (40.00) |  |
| Adult age, mean (SD) | 36.51 (4.99) | 33.55 (3.46) | 37.23 (4.97) | 41.50 (4.93) | < .001 |
| Child age, mean (SD) | 5.62 (1.00) | 5.35 (1.09) | 5.75 (0.96) | 5.00 (0.47) | .023 |
| Education (edu), mean (SD) | 4.55 (0.89) | 4.65 (0.82) | 4.54 (0.88) | 4.00 (1.33) | .374 |
| Employ status, n (%) |  |  |  | < .001 | |
| Full time | 207 (83.81) | 52 (86.67) | 150 (84.75) | 5 (50.00) |  |
| Part time | 7 (2.83) | 4 (6.67) | 3 (1.69) | 0 (0.00) |  |
| Unemployed | 13 (5.26) | 0 (0.00) | 8 (4.52) | 5 (50.00) |  |
| Self-employed | 18 (7.29) | 4 (6.67) | 14 (7.91) | 0 (0.00) |  |
| Other | 2 (0.81) | 0 (0.00) | 2 (1.13) | 0 (0.00) |  |
| Ethnicity, n (%) |  |  |  |  | .225 |
| Han | 246 (99.60) | 60 (100.00) | 176 (99.44) | 10 (100.00) |  |
| Other | 1 (0.40) | 0 (0.00) | 1 (0.56) | 0 (0.00) |  |
| Children Function difficulty, n (%) |  |  |  |  | .220 |
| Yes | 56 (22.67) | 18 (30.00) | 36 (20.34) | 2 (20.00) |  |
| No | 191 (77.33) | 42 (70.00) | 141 (79.66) | 8 (80.00) |  |
| **Primary outcomes, mean (SD)** | | | | | |
| Early Learning and Stimulation, | 22.10 (9.62) | 24.88 (9.40) | 21.21 (9.58) | 21.20 (9.25) | .049 |
| Caregiver-Perpetrated Violence: Total | 14.06 (5.36) | 13.62 (5.65) | 14.09 (5.14) | 16.30 (7.15) | .469 |
| Caregiver-Perpetrated Violence: Physical | 5.54 (2.43) | 5.37 (2.54) | 5.53 (2.29) | 6.80 (3.82) | .329 |
| Caregiver-Perpetrated Violence: Emotional | 8.52 (3.51) | 8.25 (3.51) | 8.56 (3.48) | 9.50 (4.14) | .688 |
| **Secondary outcomes, mean (SD)** |  |  |  |  |  |
| Attitude towards Corporal Punishment | 2.56 (1.32) | 2.33 (1.30) | 2.64 (1.32) | 2.40 (1.43) | .290 |
| Child Behavior: Total | 12.33 (4.19) | 12.02 (4.26) | 12.34 (4.12) | 14.10 (5.15) | .370 |
| Child Behavior: Internalizing | 5.74 (2.02) | 5.73 (1.71) | 5.72 (2.14) | 6.30 (1.57) | .651 |
| Child Behavior: Externalizing | 6.59 (3.16) | 6.28 (3.53) | 6.62 (2.96) | 7.80 (4.24) | .424 |
| Child Behavior: Emotional problem | 3.78 (1.37) | 3.80 (1.13) | 3.76 (1.44) | 3.90 (1.37) | .944 |
| Child Behavior: Conduct problem | 2.36 (1.40) | 2.25 (1.41) | 2.35 (1.34) | 3.10 (2.13) | .229 |
| Child Behavior: Hyperactivity | 4.23 (2.40) | 4.03 (2.64) | 4.27 (2.31) | 4.70 (2.63) | .723 |
| Child Behavior: Peer problem | 1.97 (1.40) | 1.93 (1.26) | 1.95 (1.44) | 2.40 (1.43) | .650 |
| Child Behavior: Prosocial behavior | 7.19 (1.92) | 7.07 (1.93) | 7.20 (1.93) | 7.70 (1.57) | .652 |
| Parenting Practices: Total | 56.40 (7.33) | 58.25 (7.43) | 55.78 (7.31) | 56.30 (5.74) | .117 |
| Parenting Practices: Positive | 23.94 (3.36) | 24.30 (3.21) | 23.83 (3.39) | 23.60 (3.75) | .726 |
| Parenting Practices: Involvement | 32.47 (4.93) | 33.95 (5.00) | 31.95 (4.88) | 32.70 (3.89) | .040 |
| Parental Mental Health: Total | 3.74 (5.34) | 2.73 (3.57) | 4.12 (5.85) | 3.00 (4.03) | .321 |
| Parental Mental Health: Depression, | 1.99 (3.18) | 1.40 (2.16) | 2.23 (3.49) | 1.40 (2.12) | .261 |
| Parental Mental Health: Anxiety | 1.75 (2.71) | 1.33 (2.10) | 1.90 (2.92) | 1.60 (2.07) | .475 |
| Parenting Stress | 38.29 (6.97) | 36.42 (6.29) | 38.88 (7.03) | 39.10 (8.56) | .073 |
| Family Functioning | 2.64 (2.42) | 2.48 (2.33) | 2.69 (2.44) | 2.60 (2.59) | .867 |

## Table S7 The baseline association between number of children and outcomes in intervention group (N=247)

| Outcomes | Number of children (Ref. 1 child) | Estimate | P | 95% CI |
| --- | --- | --- | --- | --- |
| **Primary outcomes** |  |  |  |  |
| Early Learning and Stimulation | 2 children | -2.906 | .085 | (-6.310, 0.497) |
|  | 3+ children | -2.491 | .552 | (-11.609, 6.628) |
| Caregiver-Perpetrated Violence: Total | 2 children | 0.068 | .312 | (-0.064, 0.200) |
|  | 3+ children | 0.210 | .314 | (-0.198, 0.618) |
| Caregiver-Perpetrated Violence: Physical | 2 children | 1.096 | .183 | (0.957, 1.255) |
|  | 3+ children | 1.287 | .290 | (0.806, 2.054) |
| Caregiver-Perpetrated Violence: Emotional | 2 children | 1.052 | .539 | (0.895, 1.236) |
|  | 3+ children | 1.218 | .397 | (0.772, 1.922) |
| **Secondary outcomes** |  |  |  |  |
| Parental Mental Health: Total | 2 children | 1.507 | .020 | (0.299, 2.716) |
|  | 3+ children | 0.168 | .938 | (-4.568, 4.904) |
| Parental Mental Health: Depression | 2 children | 0.741 | .024 | (0.121, 1.360) |
|  | 3+ children | -0.426 | .707 | (-2.910, 2.057) |
| Parental Mental Health: Anxiety | 2 children | 0.767 | .034 | (0.072, 1.461) |
|  | 3+ children | 0.594 | .607 | (-1.928, 3.117) |
| Child Behavior: Total | 2 children | 0.323 | .732 | (-1.747, 2.393) |
|  | 3+ children | 1.250 | .513 | (-2.898, 5.398) |
| Child Behavior: Emotional problem | 2 children | -0.032 | .871 | (-0.458, 0.395) |
|  | 3+ children | 0.282 | .398 | (-0.437, 1.001) |
| Child Behavior: Conduct problem | 2 children | 0.248 | .478 | (-0.510, 1.007) |
|  | 3+ children | 1.177 | .110 | (-0.323, 2.678) |
| Child Behavior: Hyperactivity | 2 children | -0.045 | .919 | (-1.019, 0.929) |
|  | 3+ children | -0.463 | .679 | (-2.913, 1.988) |
| Child Behavior: Peer problem | 2 children | 0.151 | .596 | (-0.472, 0.775) |
|  | 3+ children | 0.253 | .629 | (-0.893, 1.399) |
| Child Behavior: Prosocial behavior | 2 children | 0.293 | .509 | (-0.671, 1.258) |
|  | 3+ children | 1.244 | .098 | (-0.280, 2.769) |
| Child Behavior: Externalizing behavior | 2 children | 0.203 | .777 | (-1.374, 1.781) |
|  | 3+ children | 0.715 | .664 | (-2.886, 4.315) |
| Child Behavior: Internalizing behavior | 2 children | 0.120 | .766 | (-0.763, 1.002) |
|  | 3+ children | 0.535 | .242 | (-0.430, 1.500) |
| Parenting Practices: Total | 2 children | -1.990 | .039 | (-3.856, -0.124) |
|  | 3+ children | 0.278 | .929 | (-6.568, 7.125) |
| Parenting Practices: Positive parenting | 2 children | -0.055 | .875 | (-0.828, 0.718) |
|  | 3+ children | 0.635 | .668 | (-2.608, 3.877) |
| Parenting Practices: Parental involvement | 2 children | -1.935 | .004 | (-3.094, -0.776) |
|  | 3+ children | -0.356 | .872 | (-5.217, 4.504) |
| Attitude towards Corporal Punishment | 2 children | 0.400 | .037 | (0.031, 0.769) |
|  | 3+ children | 0.437 | .386 | (-0.648, 1.522) |
| Parenting Stress | 2 children | 2.458 | .030 | (0.295, 4.621) |
|  | 3+ children | 2.762 | .406 | (-4.408, 9.932) |
| Family Functioning | 2 children | -0.105 | .692 | (-0.688, 0.477) |
|  | 3+ children | -0.753 | .260 | (-2.169, 0.663) |

**Note:** All models were adjusted for caregiver age, child age, marital status, caregiver gender, child gender, educational attainment, ethnicity, hukou status, employment status, and child disability. Total caregiver-perpetrated violence was estimated using negative binomial regression, whereas physical caregiver-perpetrated violence and emotional caregiver-perpetrated violence were estimated using Poisson regression and are reported as incidence rate ratios (IRRs). Other outcomes were estimated using linear mixed-effects regression.

## Table S8 Differences in post-intervention trajectories for secondary outcomes by number of children within the intervention group (N = 806)

| Secondary outcomes | Time × number of children  （Ref. T0 × 1 child) | Estimate | P | 95%CI |
| --- | --- | --- | --- | --- |
| Attitude towards Corporal Punishment | T1 × 2 children | -0.113 | .539 | (-0.474, 0.248) |
|  | T1 × 3+ children | 0.232 | .680 | (-0.870, 1.334) |
|  | T2 × 2 children | -0.244 | .217 | (-0.630, 0.143) |
|  | T2× 3+ children | 0.374 | .213 | (-0.215, 0.963) |
|  | T3 × 2 children | -0.319 | .174 | (-0.779, 0.141) |
|  | T3 × 3+ children | -0.076 | .849 | (-0.854, 0.703) |
| Child Behavior: Total | T1 × 2 children | -0.916 | .124 | (-2.082, 0.251) |
|  | T1 × 3+ children | -3.076 | .002 | (-4.990, -1.162) |
|  | T2 × 2 children | -1.275 | .052 | (-2.562, 0.012) |
|  | T2× 3+ children | 0.003 | .998 | (-2.980, 2.986) |
|  | T3 × 2 children | -1.329 | .034 | (-2.561, -0.097) |
|  | T3 × 3+ children | -3.549 | .009 | (-6.224, -0.874) |
| Child Behavior: Internalizing | T1 × 2 children | -0.500 | .125 | (-1.139, 0.138) |
|  | T1 × 3+ children | -1.368 | <.001 | (-2.108, -0.628) |
|  | T2 × 2 children | -0.466 | .162 | (-1.119, 0.187) |
|  | T2× 3+ children | -0.597 | .481 | (-2.257, 1.064) |
|  | T3 × 2 children | -0.401 | .224 | (-1.047, 0.245) |
|  | T3 × 3+ children | -1.026 | .157 | (-2.446, 0.394) |
| Child Behavior: Externalizing behavior | T1 × 2 children | -0.420 | .330 | (-1.264, 0.425) |
|  | T1 × 3+ children | -1.693 | .027 | (-3.192, -0.193) |
|  | T2 × 2 children | -0.782 | .108 | (-1.736, 0.171) |
|  | T2× 3+ children | 0.620 | .555 | (-1.440, 2.679) |
|  | T3 × 2 children | -0.912 | .056 | (-1.847, 0.023) |
|  | T3 × 3+ children | -2.535 | .009 | (-4.428, -0.642) |
| Child Behavior: Emotional problem | T1 × 2 children | -0.127 | .502 | (-0.498, 0.244) |
|  | T1 × 3+ children | -0.235 | .531 | (-0.970, 0.500) |
|  | T2 × 2 children | -0.164 | .456 | (-0.594, 0.266) |
|  | T2× 3+ children | -0.342 | .618 | (-1.686, 1.001) |
|  | T3 × 2 children | -0.131 | .569 | (-0.582, 0.320) |
|  | T3 × 3+ children | -0.438 | .544 | (-1.854, 0.977) |
| Child Behavior: Conduct problem | T1 × 2 children | -0.198 | .365 | (-0.627, 0.231) |
|  | T1 × 3+ children | -0.807 | .044 | (-1.592, -0.023) |
|  | T2 × 2 children | -0.367 | .117 | (-0.825, 0.091) |
|  | T2× 3+ children | 0.071 | .901 | (-1.050, 1.192) |
|  | T3 × 2 children | -0.203 | .443 | (-0.724, 0.317) |
|  | T3 × 3+ children | -0.451 | .305 | (-1.312, 0.410) |
| Child Behavior: Hyperactivity | T1 × 2 children | -0.223 | .478 | (-0.839, 0.393) |
|  | T1 × 3+ children | -0.883 | .107 | (-1.956, 0.191) |
|  | T2 × 2 children | -0.423 | .188 | (-1.052, 0.206) |
|  | T2× 3+ children | 0.536 | .393 | (-0.694, 1.766) |
|  | T3 × 2 children | -0.722 | .031 | (-1.378, -0.066) |
|  | T3 × 3+ children | -2.155 | .004 | (-3.641, -0.669) |
| Child Behavior: Peer problem | T1 × 2 children | -0.365 | .143 | (-0.853, 0.123) |
|  | T1 × 3+ children | -1.139 | .009 | (-1.999, -0.279) |
|  | T2 × 2 children | -0.286 | .247 | (-0.771, 0.199) |
|  | T2× 3+ children | -0.284 | .636 | (-1.461, 0.892) |
|  | T3 × 2 children | -0.244 | .303 | (-0.708, 0.220) |
|  | T3 × 3+ children | -0.646 | .160 | (-1.548, 0.255) |
| Child Behavior: Prosocial behavior | T1 × 2 children | -0.080 | .798 | (-0.693, 0.533) |
|  | T1 × 3+ children | -0.177 | .743 | (-1.236, 0.882) |
|  | T2 × 2 children | 0.436 | .183 | (-0.206, 1.077) |
|  | T2× 3+ children | 0.479 | .380 | (-0.590, 1.549) |
|  | T3 × 2 children | 0.025 | .939 | (-0.623, 0.674) |
|  | T3 × 3+ children | -0.494 | .282 | (-1.392, 0.405) |
| Parenting Practices: Total | T1 × 2 children | 0.638 | .614 | (-1.843, 3.118) |
|  | T1 × 3+ children | 1.120 | .639 | (-3.559, 5.799) |
|  | T2 × 2 children | 2.141 | .140 | (-0.704, 4.986) |
|  | T2× 3+ children | -1.497 | .607 | (-7.196, 4.203) |
|  | T3 × 2 children | 1.338 | .356 | (-1.504, 4.181) |
|  | T3 × 3+ children | 4.007 | .160 | (-1.579, 9.593) |
| Parenting Practices: Positive parenting | T1 × 2 children | -0.117 | .852 | (-1.342, 1.108) |
|  | T1 × 3+ children | 0.837 | .644 | (-2.717, 4.391) |
|  | T2 × 2 children | 0.239 | .718 | (-1.057, 1.535) |
|  | T2× 3+ children | -0.013 | .993 | (-2.823, 2.798) |
|  | T3 × 2 children | 0.033 | .960 | (-1.228, 1.294) |
|  | T3 × 3+ children | 1.705 | .257 | (-1.242, 4.652) |
| Parenting Practices: Parental involvement | T1 × 2 children | 0.750 | .325 | (-0.743, 2.243) |
|  | T1 × 3+ children | 0.326 | .766 | (-1.826, 2.479) |
|  | T2 × 2 children | 1.896 | .043 | (0.062, 3.731) |
|  | T2× 3+ children | -1.449 | .461 | (-5.304, 2.407) |
|  | T3 × 2 children | 1.290 | .173 | (-0.565, 3.146) |
|  | T3 × 3+ children | 2.335 | .187 | (-1.131, 5.802) |
| Parental Mental Health: Total | T1 × 2 children | -0.817 | .186 | (-2.028, 0.393) |
|  | T1 × 3+ children | -0.601 | .479 | (-2.265, 1.064) |
|  | T2 × 2 children | -1.218 | .036 | (-2.359, -0.077) |
|  | T2× 3+ children | 0.657 | .706 | (-2.755, 4.069) |
|  | T3 × 2 children | -1.035 | .241 | (-2.766, 0.696) |
|  | T3 × 3+ children | -0.690 | .570 | (-3.068, 1.688) |
| Parental Mental Health: Depression | T1 × 2 children | -0.481 | .222 | (-1.254, 0.291) |
|  | T1 × 3+ children | 0.203 | .695 | (-0.812, 1.217) |
|  | T2 × 2 children | -0.838 | .019 | (-1.540, -0.136) |
|  | T2× 3+ children | 0.351 | .712 | (-1.515, 2.217) |
|  | T3 × 2 children | -0.844 | .142 | (-1.971, 0.282) |
|  | T3 × 3+ children | -1.077 | .122 | (-2.442, 0.288) |
| Parental Mental Health: Anxiety | T1 × 2 children | -0.365 | .313 | (-1.074, 0.344) |
|  | T1 × 3+ children | -0.830 | .222 | (-2.162, 0.502) |
|  | T2 × 2 children | -0.404 | .220 | (-1.049, 0.242) |
|  | T2× 3+ children | 0.278 | .753 | (-1.457, 2.013) |
|  | T3 × 2 children | -0.242 | .575 | (-1.087, 0.604) |
|  | T3 × 3+ children | 0.303 | .691 | (-1.191, 1.798) |
| Parenting Stress | T1 × 2 children | -0.554 | .650 | (-2.952, 1.843) |
|  | T1 × 3+ children | -2.505 | .342 | (-7.677, 2.666) |
|  | T2 × 2 children | -3.551 | .016 | (-6.447, -0.655) |
|  | T2× 3+ children | -1.323 | .803 | (-11.696, 9.050) |
|  | T3 × 2 children | 0.471 | .730 | (-2.202, 3.145) |
|  | T3 × 3+ children | -0.472 | .817 | (-4.458, 3.515) |
| Family Functioning | T1 × 2 children | -0.158 | .652 | (-0.844, 0.529) |
|  | T1 × 3+ children | 0.173 | .742 | (-0.853, 1.199) |
|  | T2 × 2 children | -0.394 | .314 | (-1.160, 0.373) |
|  | T2× 3+ children | 0.695 | .313 | (-0.656, 2.046) |
|  | T3 × 2 children | -0.146 | .747 | (-1.033, 0.741) |
|  | T3 × 3+ children | 1.151 | .162 | (-0.464, 2.767) |

## Note: The reference combination was baseline (T0) in one-child families. All models were adjusted for caregiver age, child age, marital status, caregiver gender, child gender, educational attainment, ethnicity, hukou status, employment status, and child disability. Secondary outcomes were estimated using linear mixed-effects regression and are reported as regression coefficients. Robust standard errors were used. CI = confidence interval.

## Table S9 Differences in post-intervention trajectories for primary outcomes by number of children within the intervention group,adjusted for grandparent caregiving (N = 806)

| **Primary outcomes** | **Time × Number of children**  **（Ref. T0 × 1 child)** | **Estimate** | **P** | **95%CI** |
| --- | --- | --- | --- | --- |
| Early Learning and Stimulation | T1 × 2 children | 1.849 | .151 | (-0.673, 4.372) |
|  | T1 × 3+ children | 4.479 | .071 | (-0.384, 9.341) |
|  | T2 × 2 children | 3.963 | .002 | (1.463, 6.463) |
|  | T2× 3+ children | 5.671 | .032 | (0.482, 10.860) |
|  | T3 × 2 children | 2.650 | .087 | (-0.385, 5.686) |
|  | T3 × 3+ children | 4.110 | .090 | (-0.642, 8.861) |
| Caregiver-Perpetrated Violence: Total | T1 × 2 children | -0.115 | .373 | (-0.366, 0.137) |
|  | T1 × 3+ children | -0.172 | .659 | (-0.936, 0.592) |
|  | T2 × 2 children | -0.294 | .026 | (-0.553, -0.035) |
|  | T2× 3+ children | 0.053 | .859 | (-0.532, 0.639) |
|  | T3 × 2 children | -0.072 | .672 | (-0.406, 0.262) |
|  | T3 × 3+ children | -0.718 | .054 | (-1.448, 0.012) |
| Caregiver-Perpetrated Violence: Physical | T1 × 2 children | 0.905 | .651 | (0.589, 1.392) |
|  | T1 × 3+ children | 0.975 | .965 | (0.314, 3.022) |
|  | T2 × 2 children | 0.983 | .938 | (0.642, 1.506) |
|  | T2× 3+ children | 1.535 | .160 | (0.844, 2.792) |
|  | T3 × 2 children | 0.733 | .296 | (0.409, 1.313) |
|  | T3 × 3+ children | 0.452 | .194 | (0.137, 1.496) |
| Caregiver-Perpetrated Violence: Emotional | T1 × 2 children | 0.900 | .384 | (0.710, 1.141) |
|  | T1 × 3+ children | 0.956 | .927 | (0.367, 2.490) |
|  | T2 × 2 children | 0.672 | .002 | (0.524, 0.861) |
|  | T2× 3+ children | 0.950 | .828 | (0.601, 1.504) |
|  | T3 × 2 children | 0.966 | .831 | (0.707, 1.321) |
|  | T3 × 3+ children | 0.516 | .034 | (0.280, 0.952) |

he reference combination was baseline (T0) in one-child families. All models were adjusted for caregiver age, child age, marital status, caregiver gender, child gender, educational attainment, ethnicity, hukou status, employment status, child disability, and grandparent caregiving. Early learning and stimulation is reported as a regression coefficient. Total caregiver-perpetrated violence was estimated using mixed-effects negative binomial regression and is reported on the log-count scale, whereas physical and emotional caregiver-perpetrated violence were estimated using mixed-effects Poisson regression and are reported as incidence rate ratios (IRRs). Robust standard errors were used. CI = confidence interval; IRR = incidence rate ratio.

## Table S10 Differences in post-intervention trajectories for secondary outcomes by number of children within the intervention group, adjusted for grandparent caregiving (N = 806)

| **Secondary outcomes** | **Time × number of children**  **（Ref. T0 × 1 child)** | **Estimate** | **P** | **95%CI** |
| --- | --- | --- | --- | --- |
| Attitude towards Corporal Punishment | T1 × 2 children | -0.113 | .540 | (-0.474, 0.248) |
|  | T1 × 3+ children | 0.229 | .684 | (-0.873, 1.331) |
|  | T2 × 2 children | -0.244 | .216 | (-0.630, 0.143) |
|  | T2× 3+ children | 0.370 | .221 | (-0.222, 0.962) |
|  | T3 × 2 children | -0.318 | .176 | (-0.777, 0.142) |
|  | T3 × 3+ children | -0.077 | .847 | (-0.857, 0.704) |
| Child Behavior: Total | T1 × 2 children | -0.917 | .123 | (-2.084, 0.249) |
|  | T1 × 3+ children | -3.063 | .002 | (-4.977, -1.150) |
|  | T2 × 2 children | -1.275 | .052 | (-2.561, 0.011) |
|  | T2× 3+ children | 0.021 | .989 | (-2.965, 3.007) |
|  | T3 × 2 children | -1.337 | .033 | (-2.567, -0.108) |
|  | T3 × 3+ children | -3.547 | .009 | (-6.222, -0.871) |
| Child Behavior: Internalizing | T1 × 2 children | -0.501 | .124 | (-1.139, 0.138) |
|  | T1 × 3+ children | -1.362 | <.001 | (-2.105, -0.618) |
|  | T2 × 2 children | -0.466 | .163 | (-1.119, 0.188) |
|  | T2× 3+ children | -0.588 | .488 | (-2.251, 1.075) |
|  | T3 × 2 children | -0.405 | .219 | (-1.050, 0.241) |
|  | T3 × 3+ children | -1.025 | .157 | (-2.444, 0.395) |
| Child Behavior: Externalizing behavior | T1 × 2 children | -0.421 | .329 | (-1.265, 0.424) |
|  | T1 × 3+ children | -1.685 | .027 | (-3.183, -0.187) |
|  | T2 × 2 children | -0.782 | .108 | (-1.735, 0.171) |
|  | T2× 3+ children | 0.631 | .548 | (-1.429, 2.691) |
|  | T3 × 2 children | -0.917 | .054 | (-1.850, 0.015) |
|  | T3 × 3+ children | -2.534 | .009 | (-4.428, -0.640) |
| Child Behavior: Emotional problem | T1 × 2 children | -0.127 | .501 | (-0.499, 0.244) |
|  | T1 × 3+ children | -0.232 | .536 | (-0.968, 0.503) |
|  | T2 × 2 children | -0.164 | .456 | (-0.593, 0.266) |
|  | T2× 3+ children | -0.338 | .622 | (-1.683, 1.006) |
|  | T3 × 2 children | -0.133 | .564 | (-0.584, 0.318) |
|  | T3 × 3+ children | -0.438 | .544 | (-1.853, 0.978) |
| Child Behavior: Conduct problem | T1 × 2 children | -0.198 | .364 | (-0.627, 0.230) |
|  | T1 × 3+ children | -0.804 | .045 | (-1.590, -0.019) |
|  | T2 × 2 children | -0.367 | .116 | (-0.825, 0.091) |
|  | T2× 3+ children | 0.075 | .895 | (-1.046, 1.197) |
|  | T3 × 2 children | -0.206 | .438 | (-0.725, 0.314) |
|  | T3 × 3+ children | -0.451 | .305 | (-1.312, 0.411) |
| Child Behavior: Hyperactivity | T1 × 2 children | -0.224 | .477 | (-0.840, 0.392) |
|  | T1 × 3+ children | -0.877 | .108 | (-1.947, 0.193) |
|  | T2 × 2 children | -0.423 | .188 | (-1.052, 0.206) |
|  | T2× 3+ children | 0.545 | .385 | (-0.685, 1.774) |
|  | T3 × 2 children | -0.726 | .030 | (-1.380, -0.072) |
|  | T3 × 3+ children | -2.154 | .005 | (-3.641, -0.666) |
| Child Behavior: Peer problem | T1 × 2 children | -0.365 | .143 | (-0.854, 0.123) |
|  | T1 × 3+ children | -1.136 | .010 | (-1.996, -0.275) |
|  | T2 × 2 children | -0.286 | .248 | (-0.771, 0.199) |
|  | T2× 3+ children | -0.279 | .642 | (-1.455, 0.898) |
|  | T3 × 2 children | -0.247 | .297 | (-0.711, 0.217) |
|  | T3 × 3+ children | -0.647 | .160 | (-1.549, 0.255) |
| Child Behavior: Prosocial behavior | T1 × 2 children | -0.080 | .799 | (-0.693, 0.534) |
|  | T1 × 3+ children | -0.186 | .730 | (-1.241, 0.869) |
|  | T2 × 2 children | 0.436 | .182 | (-0.205, 1.076) |
|  | T2× 3+ children | 0.467 | .389 | (-0.596, 1.531) |
|  | T3 × 2 children | 0.032 | .924 | (-0.614, 0.677) |
|  | T3 × 3+ children | -0.496 | .274 | (-1.384, 0.393) |
| Parenting Practices: Total | T1 × 2 children | 0.645 | .610 | (-1.836, 3.126) |
|  | T1 × 3+ children | 1.081 | .648 | (-3.564, 5.725) |
|  | T2 × 2 children | 2.140 | .140 | (-0.702, 4.981) |
|  | T2× 3+ children | -1.563 | .589 | (-7.238, 4.113) |
|  | T3 × 2 children | 1.379 | .339 | (-1.450, 4.207) |
|  | T3 × 3+ children | 4.008 | .160 | (-1.584, 9.599) |
| Parenting Practices: Positive parenting | T1 × 2 children | -0.115 | .854 | (-1.340, 1.110) |
|  | T1 × 3+ children | 0.824 | .649 | (-2.721, 4.370) |
|  | T2 × 2 children | 0.238 | .718 | (-1.056, 1.533) |
|  | T2× 3+ children | -0.033 | .982 | (-2.840, 2.774) |
|  | T3 × 2 children | 0.043 | .946 | (-1.212, 1.299) |
|  | T3 × 3+ children | 1.705 | .257 | (-1.244, 4.654) |
| Parenting Practices: Parental involvement | T1 × 2 children | 0.757 | .320 | (-0.736, 2.250) |
|  | T1 × 3+ children | 0.295 | .786 | (-1.832, 2.422) |
|  | T2 × 2 children | 1.897 | .043 | (0.063, 3.730) |
|  | T2× 3+ children | -1.498 | .444 | (-5.329, 2.334) |
|  | T3 × 2 children | 1.323 | .161 | (-0.527, 3.173) |
|  | T3 × 3+ children | 2.333 | .186 | (-1.126, 5.792) |
| Parental Mental Health: Total | T1 × 2 children | -0.819 | .185 | (-2.030, 0.391) |
|  | T1 × 3+ children | -0.588 | .488 | (-2.248, 1.072) |
|  | T2 × 2 children | -1.218 | .036 | (-2.358, -0.078) |
|  | T2× 3+ children | 0.676 | .697 | (-2.728, 4.080) |
|  | T3 × 2 children | -1.045 | .237 | (-2.778, 0.689) |
|  | T3 × 3+ children | -0.687 | .571 | (-3.064, 1.689) |
| Parental Mental Health: Depression | T1 × 2 children | -0.482 | .221 | (-1.255, 0.290) |
|  | T1 × 3+ children | 0.210 | .684 | (-0.803, 1.224) |
|  | T2 × 2 children | -0.838 | .019 | (-1.540, -0.136) |
|  | T2× 3+ children | 0.362 | .703 | (-1.500, 2.224) |
|  | T3 × 2 children | -0.850 | .140 | (-1.978, 0.278) |
|  | T3 × 3+ children | -1.076 | .122 | (-2.440, 0.289) |
| Parental Mental Health: Anxiety | T1 × 2 children | -0.366 | .312 | (-1.075, 0.343) |
|  | T1 × 3+ children | -0.824 | .224 | (-2.152, 0.505) |
|  | T2 × 2 children | -0.404 | .220 | (-1.049, 0.242) |
|  | T2× 3+ children | 0.287 | .745 | (-1.443, 2.017) |
|  | T3 × 2 children | -0.246 | .569 | (-1.093, 0.601) |
|  | T3 × 3+ children | 0.305 | .689 | (-1.188, 1.798) |
| Parenting Stress | T1 × 2 children | -0.555 | .650 | (-2.953, 1.843) |
|  | T1 × 3+ children | -2.500 | .343 | (-7.671, 2.671) |
|  | T2 × 2 children | -3.551 | .016 | (-6.446, -0.655) |
|  | T2× 3+ children | -1.316 | .804 | (-11.691, 9.060) |
|  | T3 × 2 children | 0.467 | .732 | (-2.206, 3.141) |
|  | T3 × 3+ children | -0.471 | .817 | (-4.457, 3.515) |
| Family Functioning | T1 × 2 children | -0.158 | .652 | (-0.844, 0.528) |
|  | T1 × 3+ children | 0.174 | .739 | (-0.851, 1.200) |
|  | T2 × 2 children | -0.394 | .314 | (-1.160, 0.373) |
|  | T2× 3+ children | 0.698 | .312 | (-0.654, 2.049) |
|  | T3 × 2 children | -0.147 | .745 | (-1.035, 0.740) |
|  | T3 × 3+ children | 1.152 | .162 | (-0.464, 2.767) |

Note: The reference combination was baseline (T0) in one-child families. All models were adjusted for caregiver age, child age, marital status, caregiver gender, child gender, educational attainment, ethnicity, hukou status, employment status, child disability, and grandparent caregiving. All outcomes were estimated using linear mixed-effects regression and are reported as regression coefficients. Robust standard errors were used. CI = confidence interval.

| ***Table S11 Approximate MDEs for key interaction tests: intervention-group trajectories for primary outcomes*** | | | | |
| --- | --- | --- | --- | --- |
| **Outcome** | **Interaction term** | **Estimate** | **95% CI** | **Approximate MDE (80% power)** |
| Early Learning and Stimulation | T1 × 2 children | 1.836 | -0.688 to 4.360 | 3.61 scale points |
| Early Learning and Stimulation | T1 × 3+ children | 4.528 | -0.366 to 9.423 | 6.99 scale points |
| Early Learning and Stimulation | T2 × 2 children | 3.966 | 1.468 to 6.463 | 3.57 scale points |
| Early Learning and Stimulation | T2 × 3+ children | 5.749 | 0.536 to 10.962 | 7.45 scale points |
| Early Learning and Stimulation | T3 × 2 children | 2.598 | -0.457 to 5.653 | 4.36 scale points |
| Early Learning and Stimulation | T3 × 3+ children | 4.11 | -0.662 to 8.881 | 6.82 scale points |
| Caregiver-Perpetrated Violence: Total | T1 × 2 children | -0.115 | -0.367 to 0.137 | IRR ≤0.70 or ≥1.43 |
| Caregiver-Perpetrated Violence: Total | T1 × 3+ children | -0.169 | -0.932 to 0.595 | IRR ≤0.34 or ≥2.98 |
| Caregiver-Perpetrated Violence: Total | T2 × 2 children | -0.294 | -0.553 to -0.036 | IRR ≤0.69 or ≥1.45 |
| Caregiver-Perpetrated Violence: Total | T2 × 3+ children | 0.057 | -0.528 to 0.642 | IRR ≤0.43 or ≥2.31 |
| Caregiver-Perpetrated Violence: Total | T3 × 2 children | -0.074 | -0.410 to 0.261 | IRR ≤0.62 or ≥1.61 |
| Caregiver-Perpetrated Violence: Total | T3 × 3+ children | -0.719 | -1.447 to 0.010 | IRR ≤0.35 or ≥2.83 |
| Caregiver-Perpetrated Violence: Physical | T1 × 2 children | IRR 0.905 | 0.589 to 1.392 | IRR ≤0.54 or ≥1.85 |
| Caregiver-Perpetrated Violence: Physical | T1 × 3+ children | IRR 0.975 | 0.315 to 3.023 | IRR ≤0.20 or ≥5.03 |
| Caregiver-Perpetrated Violence: Physical | T2 × 2 children | IRR 0.983 | 0.642 to 1.506 | IRR ≤0.54 or ≥1.84 |
| Caregiver-Perpetrated Violence: Physical | T2 × 3+ children | IRR 1.536 | 0.845 to 2.794 | IRR ≤0.43 or ≥2.35 |
| Caregiver-Perpetrated Violence: Physical | T3 × 2 children | IRR 0.732 | 0.408 to 1.313 | IRR ≤0.43 or ≥2.30 |
| Caregiver-Perpetrated Violence: Physical | T3 × 3+ children | IRR 0.452 | 0.137 to 1.495 | IRR ≤0.18 or ≥5.51 |
| Caregiver-Perpetrated Violence: Emotional | T1 × 2 children | IRR 0.900 | 0.710 to 1.141 | IRR ≤0.71 or ≥1.40 |
| Caregiver-Perpetrated Violence: Emotional | T1 × 3+ children | IRR 0.957 | 0.367 to 2.494 | IRR ≤0.25 or ≥3.93 |
| Caregiver-Perpetrated Violence: Emotional | T2 × 2 children | IRR 0.672 | 0.524 to 0.861 | IRR ≤0.70 or ≥1.43 |
| Caregiver-Perpetrated Violence: Emotional | T2 × 3+ children | IRR 0.952 | 0.602 to 1.506 | IRR ≤0.52 or ≥1.93 |
| Caregiver-Perpetrated Violence: Emotional | T3 × 2 children | IRR 0.966 | 0.706 to 1.321 | IRR ≤0.64 or ≥1.56 |
| Caregiver-Perpetrated Violence: Emotional | T3 × 3+ children | IRR 0.517 | 0.281 to 0.952 | IRR ≤0.42 or ≥2.39 |

Note: MDE = minimum detectable effect size. Approximate MDEs were derived from the standard errors implied by the 95% confidence intervals, assuming a two-sided α of 0.05 and 80% power. Because all outcomes shown in this table are continuous scale scores, MDEs are expressed as absolute differences in the original scale units. These calculations were used to contextualize statistical precision and should not be interpreted as additional hypothesis tests.

***Table S12 Approximate MDEs for key interaction tests: intervention-group trajectories for secondary interactions explicitly interpreted in the Results***

| **Outcome** | **Interaction term** | **Estimate** | **95% CI** | **Approximate MDE (80% power)** |
| --- | --- | --- | --- | --- |
| Child Behavior: Total | T1 × 3+ children | -3.076 | -4.990 to -1.162 | 2.73 scale points |
| Child Behavior: Total | T3 × 3+ children | -3.549 | -6.224 to -0.874 | 3.82 scale points |
| Child Behavior: Internalizing | T1 × 3+ children | -1.368 | -2.108 to -0.628 | 1.06 scale points |
| Child Behavior: Externalizing | T1 × 3+ children | -1.693 | -3.192 to -0.193 | 2.14 scale points |
| Child Behavior: Externalizing | T3 × 3+ children | -2.535 | -4.428 to -0.642 | 2.70 scale points |
| Child Behavior: Conduct Problem | T1 × 3+ children | -0.807 | -1.592 to -0.023 | 1.12 scale points |
| Child Behavior: Hyperactivity | T3 × 3+ children | -2.155 | -3.641 to -0.669 | 2.12 scale points |
| Child Behavior: Peer Problem | T1 × 3+ children | -1.139 | -1.999 to -0.279 | 1.23 scale points |
| Child Behavior: Total | T3 × 2 children | -1.329 | -2.561 to -0.097 | 1.76 scale points |
| Child Behavior: Hyperactivity | T3 × 2 children | -0.722 | -1.378 to -0.066 | 0.94 scale points |
| Parenting Practices: Parental Involvement | T2 × 2 children | 1.896 | 0.062 to 3.731 | 2.62 scale points |
| Parental Mental Health: Total | T2 × 2 children | -1.218 | -2.359 to -0.077 | 1.63 scale points |
| Parental Mental Health: Depression | T2 × 2 children | -0.838 | -1.540 to -0.136 | 1.00 scale points |
| Parenting Stress | T2 × 2 children | -3.551 | -6.447 to -0.655 | 4.14 scale points |

Note. MDE = minimum detectable effect size. MDEs were approximated from the 95% confidence intervals of the interaction estimates, assuming a two-sided α of 0.05 and 80% power. For continuous outcomes, MDEs represent absolute differences in scale points. For count outcomes reported as IRRs, MDEs were calculated on the log scale and transformed back to IRRs. For total caregiver-perpetrated violence in the trajectory models, estimates were presented on the log-count scale; therefore, MDEs are shown as log coefficients with equivalent detectable IRR thresholds. These calculations were used to contextualize statistical precision and should not be interpreted as additional hypothesis tests

## Table S13 Sensitivity analysis: differences in post-intervention trajectories for primary outcomes by number of children within the intervention group with unadjusting sociodemographic covariates (N = 885)

| Primary outcomes | Time × number of children  （Ref. T0 × 1 child) | Estimate | P | 95%CI |
| --- | --- | --- | --- | --- |
| Early Learning and Stimulation | T1 × 2 children | 1.574 | .195 | (-0.806, 3.954) |
|  | T1 × 3+ children | 2.322 | .305 | (-2.116, 6.759) |
|  | T2 × 2 children | 3.816 | .002 | (1.391, 6.241) |
|  | T2× 3+ children | 3.838 | .173 | (-1.688, 9.364) |
|  | T3 × 2 children | 2.321 | .107 | (-0.498, 5.140) |
|  | T3 × 3+ children | 1.651 | .506 | (-3.208, 6.509) |
| Caregiver-Perpetrated Violence: Total | T1 × 2 children | -0.147 | .222 | (-0.384, 0.089) |
|  | T1 × 3+ children | -0.163 | .620 | (-0.806, 0.480) |
|  | T2 × 2 children | -0.280 | .028 | (-0.529, -0.030) |
|  | T2× 3+ children | 0.043 | .868 | (-0.464, 0.551) |
|  | T3 × 2 children | -0.140 | .392 | (-0.460, 0.180) |
|  | T3 × 3+ children | -0.098 | .771 | (-0.762, 0.566) |
| Caregiver-Perpetrated Violence: Physical | T1 × 2 children | 0.874 | .530 | (0.573, 1.332) |
|  | T1 × 3+ children | 0.889 | .818 | (0.327, 2.417) |
|  | T2 × 2 children | 0.976 | .908 | (0.644, 1.479) |
|  | T2× 3+ children | 1.291 | .422 | (0.692, 2.409) |
|  | T3 × 2 children | 0.700 | .211 | (0.400, 1.225) |
|  | T3 × 3+ children | 0.792 | .568 | (0.356, 1.762) |
| Caregiver-Perpetrated Violence: Emotional | T1 × 2 children | 0.872 | .228 | (0.699, 1.089) |
|  | T1 × 3+ children | 0.941 | .874 | (0.445, 1.993) |
|  | T2 × 2 children | 0.687 | .002 | (0.541, 0.872) |
|  | T2× 3+ children | 0.996 | .985 | (0.669, 1.482) |
|  | T3 × 2 children | 0.914 | .554 | (0.679, 1.231) |
|  | T3 × 3+ children | 1.012 | .969 | (0.561, 1.824) |

**Note:** The reference combination was baseline (T0) in one-child families. No baseline sociodemographic covariates were included in these sensitivity models. Early learning and stimulation is reported as a regression coefficient. Total caregiver-perpetrated violence was estimated using mixed-effects negative binomial regression and is reported on the log-count scale, whereas physical and emotional caregiver-perpetrated violence were estimated using mixed-effects Poisson regression and are reported as incidence rate ratios (IRRs). Robust standard errors were used. CI = confidence interval; IRR = incidence rate ratio.

## Table S14 Sensitivity analysis: differences in post-intervention trajectories for secondary outcomes by number of children within the intervention group with unadjusting sociodemographic covariates (N = 885)

| Secondary outcomes | Time × number of children  （Ref. T0 × 1 child) | Estimate | P | 95%CI |
| --- | --- | --- | --- | --- |
| Child Behavior: Total | T1 × 2 children | -0.766 | .181 | (-1.890, 0.357) |
|  | T1 × 3+ children | -2.391 | .003 | (-3.964, -0.819) |
|  | T2 × 2 children | -1.178 | .064 | (-2.426, 0.070) |
|  | T2× 3+ children | 0.032 | .982 | (-2.688, 2.752) |
|  | T3 × 2 children | -1.453 | .026 | (-2.728, -0.177) |
|  | T3 × 3+ children | -2.918 | .032 | (-5.582, -0.254) |
| Child Behavior: Internalizing behavior | T1 × 2 children | -0.422 | .187 | (-1.048, 0.205) |
|  | T1 × 3+ children | -1.311 | .002 | (-2.127, -0.495) |
|  | T2 × 2 children | -0.394 | .267 | (-1.090, 0.302) |
|  | T2× 3+ children | -0.242 | .742 | (-1.684, 1.199) |
|  | T3 × 2 children | -0.429 | .207 | (-1.095, 0.238) |
|  | T3 × 3+ children | -1.007 | .132 | (-2.318, 0.304) |
| Child Behavior: Externalizing behavior | T1 × 2 children | -0.333 | .411 | (-1.126, 0.461) |
|  | T1 × 3+ children | -1.050 | .136 | (-2.431, 0.331) |
|  | T2 × 2 children | -0.748 | .107 | (-1.658, 0.161) |
|  | T2× 3+ children | 0.294 | .768 | (-1.660, 2.248) |
|  | T3 × 2 children | -1.002 | .032 | (-1.920, -0.084) |
|  | T3 × 3+ children | -1.914 | .070 | (-3.981, 0.153) |
| Child Behavior: Emotional problem | T1 × 2 children | -0.118 | .536 | (-0.493, 0.256) |
|  | T1 × 3+ children | -0.568 | .167 | (-1.372, 0.237) |
|  | T2 × 2 children | -0.188 | .411 | (-0.635, 0.260) |
|  | T2× 3+ children | 0.071 | .910 | (-1.151, 1.292) |
|  | T3 × 2 children | -0.205 | .387 | (-0.669, 0.259) |
|  | T3 × 3+ children | -0.227 | .705 | (-1.401, 0.948) |
| Child Behavior: Conduct problem | T1 × 2 children | -0.215 | .295 | (-0.618, 0.188) |
|  | T1 × 3+ children | -0.662 | .060 | (-1.353, 0.028) |
|  | T2 × 2 children | -0.469 | .045 | (-0.928, -0.011) |
|  | T2× 3+ children | 0.225 | .688 | (-0.873, 1.324) |
|  | T3 × 2 children | -0.386 | .145 | (-0.905, 0.133) |
|  | T3 × 3+ children | -0.630 | .149 | (-1.484, 0.225) |
| Child Behavior: Hyperactivity | T1 × 2 children | -0.116 | .700 | (-0.705, 0.473) |
|  | T1 × 3+ children | -0.397 | .453 | (-1.435, 0.641) |
|  | T2 × 2 children | -0.285 | .365 | (-0.902, 0.332) |
|  | T2× 3+ children | 0.033 | .958 | (-1.179, 1.244) |
|  | T3 × 2 children | -0.633 | .055 | (-1.280, 0.014) |
|  | T3 × 3+ children | -1.364 | .087 | (-2.924, 0.196) |
| Child Behavior: Peer problem | T1 × 2 children | -0.294 | .220 | (-0.763, 0.176) |
|  | T1 × 3+ children | -0.739 | .039 | (-1.439, -0.039) |
|  | T2 × 2 children | -0.189 | .450 | (-0.678, 0.301) |
|  | T2× 3+ children | -0.306 | .521 | (-1.237, 0.626) |
|  | T3 × 2 children | -0.205 | .388 | (-0.670, 0.260) |
|  | T3 × 3+ children | -0.799 | .032 | (-1.528, -0.070) |
| Child Behavior: Prosocial behavior | T1 × 2 children | 0.021 | .945 | (-0.583, 0.626) |
|  | T1 × 3+ children | -0.474 | .344 | (-1.456, 0.508) |
|  | T2 × 2 children | 0.368 | .240 | (-0.246, 0.983) |
|  | T2× 3+ children | -0.349 | .540 | (-1.465, 0.767) |
|  | T3 × 2 children | 0.002 | .995 | (-0.645, 0.649) |
|  | T3 × 3+ children | -0.416 | .271 | (-1.155, 0.324) |
| Parenting Practices: Total | T1 × 2 children | 0.332 | .780 | (-2.004, 2.669) |
|  | T1 × 3+ children | -1.543 | .507 | (-6.103, 3.017) |
|  | T2 × 2 children | 1.444 | .290 | (-1.232, 4.120) |
|  | T2× 3+ children | -2.529 | .301 | (-7.317, 2.260) |
|  | T3 × 2 children | 0.315 | .827 | (-2.508, 3.139) |
|  | T3 × 3+ children | -0.526 | .833 | (-5.416, 4.365) |
| Parenting Practices: Positive parenting | T1 × 2 children | -0.262 | .662 | (-1.437, 0.913) |
|  | T1 × 3+ children | -0.143 | .918 | (-2.860, 2.573) |
|  | T2 × 2 children | -0.336 | .607 | (-1.617, 0.945) |
|  | T2× 3+ children | -0.672 | .600 | (-3.186, 1.842) |
|  | T3 × 2 children | -0.525 | .429 | (-1.825, 0.775) |
|  | T3 × 3+ children | -0.512 | .704 | (-3.154, 2.130) |
| Parenting Practices: Parental involvement | T1 × 2 children | 0.590 | .409 | (-0.811, 1.990) |
|  | T1 × 3+ children | -1.382 | .329 | (-4.156, 1.392) |
|  | T2 × 2 children | 1.772 | .044 | (0.048, 3.496) |
|  | T2× 3+ children | -1.860 | .250 | (-5.033, 1.312) |
|  | T3 × 2 children | 0.816 | .368 | (-0.960, 2.592) |
|  | T3 × 3+ children | -0.014 | .992 | (-2.738, 2.711) |
| Parental Mental Health: Total | T1 × 2 children | -0.846 | .159 | (-2.024, 0.332) |
|  | T1 × 3+ children | -0.878 | .345 | (-2.698, 0.942) |
|  | T2 × 2 children | -1.453 | .012 | (-2.581, -0.324) |
|  | T2× 3+ children | 0.580 | .674 | (-2.118, 3.279) |
|  | T3 × 2 children | -0.899 | .289 | (-2.558, 0.761) |
|  | T3 × 3+ children | -0.189 | .881 | (-2.662, 2.284) |
| Parental Mental Health: Depression | T1 × 2 children | -0.495 | .201 | (-1.253, 0.264) |
|  | T1 × 3+ children | -0.406 | .458 | (-1.477, 0.665) |
|  | T2 × 2 children | -0.897 | .010 | (-1.579, -0.215) |
|  | T2× 3+ children | 0.129 | .866 | (-1.375, 1.633) |
|  | T3 × 2 children | -0.757 | .171 | (-1.841, 0.326) |
|  | T3 × 3+ children | -1.143 | .082 | (-2.430, 0.144) |
| Parental Mental Health: Anxiety | T1 × 2 children | -0.370 | .278 | (-1.037, 0.298) |
|  | T1 × 3+ children | -0.476 | .433 | (-1.666, 0.714) |
|  | T2 × 2 children | -0.571 | .083 | (-1.216, 0.075) |
|  | T2× 3+ children | 0.413 | .563 | (-0.987, 1.813) |
|  | T3 × 2 children | -0.172 | .670 | (-0.963, 0.619) |
|  | T3 × 3+ children | 0.903 | .238 | (-0.598, 2.403) |
| Parenting Stress | T1 × 2 children | -0.460 | .690 | (-2.726, 1.805) |
|  | T1 × 3+ children | -2.544 | .275 | (-7.112, 2.024) |
|  | T2 × 2 children | -2.992 | .035 | (-5.775, -0.209) |
|  | T2× 3+ children | -2.041 | .598 | (-9.626, 5.545) |
|  | T3 × 2 children | 0.751 | .570 | (-1.838, 3.341) |
|  | T3 × 3+ children | 1.595 | .488 | (-2.917, 6.107) |
| Family Functioning | T1 × 2 children | -0.086 | .801 | (-0.755, 0.583) |
|  | T1 × 3+ children | 0.329 | .582 | (-0.842, 1.499) |
|  | T2 × 2 children | -0.413 | .263 | (-1.137, 0.310) |
|  | T2× 3+ children | 0.856 | .126 | (-0.240, 1.952) |
|  | T3 × 2 children | -0.106 | .802 | (-0.938, 0.725) |
|  | T3 × 3+ children | 0.727 | .366 | (-0.848, 2.301) |
| Attitude towards Corporal Punishment | T1 × 2 children | -0.204 | .248 | (-0.550, 0.142) |
|  | T1 × 3+ children | -0.022 | .958 | (-0.837, 0.794) |
|  | T2 × 2 children | -0.292 | .120 | (-0.659, 0.076) |
|  | T2× 3+ children | 0.192 | .524 | (-0.398, 0.781) |
|  | T3 × 2 children | -0.353 | .108 | (-0.783, 0.078) |
|  | T3 × 3+ children | 0.044 | .904 | (-0.674, 0.762) |

**Note:** The reference combination was baseline (T0) in one-child families. No baseline sociodemographic covariates were included in these sensitivity models. All secondary outcomes were estimated using linear mixed-effects regression and are reported as regression coefficients. Robust standard errors were used. CI = confidence interval.

## Table S15 Sensitivity analysis: differences in post-intervention trajectories for primary outcomes by number of children within the intervention group after MICE (N = 885)

| Primary outcomes | Time × Number of children  （Ref. T0 × 1 child) | Estimate | P | 95%CI |
| --- | --- | --- | --- | --- |
| Early Learning and Stimulation | T1 × 2 children | 1.542 | .206 | (-0.846, 3.930) |
|  | T1 × 3+ children | 2.374 | .298 | (-2.094, 6.842) |
|  | T2 × 2 children | 3.839 | .002 | (1.415, 6.263) |
|  | T2× 3+ children | 3.979 | .162 | (-1.596, 9.554) |
|  | T3 × 2 children | 2.361 | .101 | (-0.462, 5.183) |
|  | T3 × 3+ children | 1.899 | .450 | (-3.027, 6.826) |
| Caregiver-Perpetrated Violence: Total | T1 × 2 children | -0.147 | .230 | (-0.386, 0.093) |
|  | T1 × 3+ children | -0.182 | .578 | (-0.825, 0.460) |
|  | T2 × 2 children | -0.268 | .036 | (-0.519, -0.018) |
|  | T2× 3+ children | 0.044 | .866 | (-0.471, 0.559) |
|  | T3 × 2 children | -0.129 | .432 | (-0.451, 0.193) |
|  | T3 × 3+ children | -0.095 | .778 | (-0.757, 0.567) |
| Caregiver-Perpetrated Violence: Physical | T1 × 2 children | 0.876 | .540 | (0.574, 1.337) |
|  | T1 × 3+ children | 0.879 | .800 | (0.323, 2.389) |
|  | T2 × 2 children | 0.988 | .955 | (0.653, 1.496) |
|  | T2× 3+ children | 1.288 | .426 | (0.690, 2.404) |
|  | T3 × 2 children | 0.707 | .225 | (0.404, 1.238) |
|  | T3 × 3+ children | 0.786 | .552 | (0.355, 1.738) |
| Caregiver-Perpetrated Violence: Emotional | T1 × 2 children | 0.872 | .230 | (0.698, 1.090) |
|  | T1 × 3+ children | 0.934 | .858 | (0.442, 1.973) |
|  | T2 × 2 children | 0.691 | .002 | (0.544, 0.877) |
|  | T2× 3+ children | 0.997 | .988 | (0.671, 1.480) |
|  | T3 × 2 children | 0.921 | .588 | (0.684, 1.240) |
|  | T3 × 3+ children | 1.007 | .982 | (0.565, 1.794) |

**Note:** All models were adjusted for caregiver age, child age, marital status, caregiver gender, child gender, educational attainment, ethnicity, hukou status, employment status, and child disability. Total caregiver-perpetrated violence was estimated using mixed-effects negative binomial regression, whereas physical caregiver-perpetrated violence and emotional caregiver-perpetrated violence were estimated using mixed-effects Poisson regression and are reported as incidence rate ratios (IRRs). Other outcomes were estimated using linear mixed-effects regression

## Table S16 Sensitivity analysis: differences in post-intervention trajectories for secondary outcomes by number of children within the intervention group after MICE (N = 885)

| Secondary outcomes | Time × number of children  （Ref. T0 × 1 child) | Estimate | P | 95%CI |
| --- | --- | --- | --- | --- |
| Attitude towards Corporal Punishment | T1 × 2 children | -0.204 | .249 | (-0.550, 0.143) |
|  | T1 × 3+ children | -0.015 | .972 | (-0.831, 0.802) |
|  | T2 × 2 children | -0.282 | .134 | (-0.649, 0.086) |
|  | T2× 3+ children | 0.196 | .517 | (-0.398, 0.790) |
|  | T3 × 2 children | -0.342 | .120 | (-0.773, 0.089) |
|  | T3 × 3+ children | 0.042 | .909 | (-0.678, 0.763) |
| Child Behavior: Total | T1 × 2 children | -0.755 | .188 | (-1.880, 0.370) |
|  | T1 × 3+ children | -2.422 | .003 | (-4.017, -0.826) |
|  | T2 × 2 children | -1.130 | .076 | (-2.377, 0.117) |
|  | T2× 3+ children | 0.037 | .979 | (-2.707, 2.782) |
|  | T3 × 2 children | -1.396 | .031 | (-2.665, -0.126) |
|  | T3 × 3+ children | -2.844 | .037 | (-5.521, -0.166) |
| Child Behavior: Internalizing | T1 × 2 children | -0.416 | .195 | (-1.044, 0.212) |
|  | T1 × 3+ children | -1.317 | .002 | (-2.134, -0.500) |
|  | T2 × 2 children | -0.375 | .291 | (-1.072, 0.321) |
|  | T2× 3+ children | -0.226 | .758 | (-1.665, 1.212) |
|  | T3 × 2 children | -0.408 | .230 | (-1.075, 0.259) |
|  | T3 × 3+ children | -0.961 | .147 | (-2.260, 0.339) |
| Child Behavior: Externalizing behavior | T1 × 2 children | -0.327 | .422 | (-1.124, 0.470) |
|  | T1 × 3+ children | -1.077 | .133 | (-2.483, 0.329) |
|  | T2 × 2 children | -0.718 | .123 | (-1.631, 0.195) |
|  | T2× 3+ children | 0.292 | .773 | (-1.689, 2.273) |
|  | T3 × 2 children | -0.963 | .040 | (-1.880, -0.046) |
|  | T3 × 3+ children | -1.873 | .079 | (-3.966, 0.220) |
| Child Behavior: Emotional problem | T1 × 2 children | -0.119 | .533 | (-0.493, 0.255) |
|  | T1 × 3+ children | -0.566 | .169 | (-1.374, 0.241) |
|  | T2 × 2 children | -0.186 | .415 | (-0.632, 0.261) |
|  | T2× 3+ children | 0.078 | .901 | (-1.138, 1.293) |
|  | T3 × 2 children | -0.202 | .394 | (-0.666, 0.262) |
|  | T3 × 3+ children | -0.195 | .747 | (-1.382, 0.991) |
| Child Behavior: Conduct problem | T1 × 2 children | -0.209 | .311 | (-0.614, 0.196) |
|  | T1 × 3+ children | -0.658 | .064 | (-1.354, 0.039) |
|  | T2 × 2 children | -0.454 | .054 | (-0.915, 0.007) |
|  | T2× 3+ children | 0.247 | .660 | (-0.853, 1.348) |
|  | T3 × 2 children | -0.361 | .173 | (-0.881, 0.158) |
|  | T3 × 3+ children | -0.573 | .189 | (-1.428, 0.282) |
| Child Behavior: Hyperactivity | T1 × 2 children | -0.116 | .699 | (-0.706, 0.473) |
|  | T1 × 3+ children | -0.428 | .425 | (-1.479, 0.623) |
|  | T2 × 2 children | -0.266 | .398 | (-0.883, 0.351) |
|  | T2× 3+ children | 0.016 | .980 | (-1.209, 1.241) |
|  | T3 × 2 children | -0.610 | .064 | (-1.256, 0.036) |
|  | T3 × 3+ children | -1.362 | .091 | (-2.943, 0.219) |
| Child Behavior: Peer problem | T1 × 2 children | -0.284 | .238 | (-0.755, 0.188) |
|  | T1 × 3+ children | -0.749 | .037 | (-1.452, -0.046) |
|  | T2 × 2 children | -0.167 | .505 | (-0.659, 0.325) |
|  | T2× 3+ children | -0.297 | .532 | (-1.229, 0.635) |
|  | T3 × 2 children | -0.180 | .450 | (-0.646, 0.286) |
|  | T3 × 3+ children | -0.786 | .033 | (-1.506, -0.065) |
| Child Behavior: Prosocial behavior | T1 × 2 children | -0.003 | .992 | (-0.607, 0.601) |
|  | T1 × 3+ children | -0.483 | .345 | (-1.486, 0.520) |
|  | T2 × 2 children | 0.343 | .275 | (-0.273, 0.958) |
|  | T2× 3+ children | -0.323 | .571 | (-1.439, 0.794) |
|  | T3 × 2 children | -0.027 | .934 | (-0.675, 0.620) |
|  | T3 × 3+ children | -0.434 | .276 | (-1.215, 0.347) |
| Parenting Practices: Total | T1 × 2 children | 0.268 | .823 | (-2.075, 2.611) |
|  | T1 × 3+ children | -1.455 | .536 | (-6.069, 3.158) |
|  | T2 × 2 children | 1.402 | .307 | (-1.287, 4.092) |
|  | T2× 3+ children | -2.319 | .344 | (-7.125, 2.486) |
|  | T3 × 2 children | 0.301 | .834 | (-2.522, 3.125) |
|  | T3 × 3+ children | -0.332 | .895 | (-5.275, 4.612) |
| Parenting Practices: Positive parenting | T1 × 2 children | -0.292 | .626 | (-1.469, 0.884) |
|  | T1 × 3+ children | -0.113 | .936 | (-2.862, 2.637) |
|  | T2 × 2 children | -0.341 | .603 | (-1.625, 0.943) |
|  | T2× 3+ children | -0.547 | .673 | (-3.086, 1.992) |
|  | T3 × 2 children | -0.513 | .436 | (-1.804, 0.778) |
|  | T3 × 3+ children | -0.360 | .791 | (-3.026, 2.306) |
| Parenting Practices: Parental involvement | T1 × 2 children | 0.551 | .443 | (-0.856, 1.958) |
|  | T1 × 3+ children | -1.326 | .349 | (-4.101, 1.450) |
|  | T2 × 2 children | 1.734 | .050 | (-0.002, 3.470) |
|  | T2× 3+ children | -1.761 | .274 | (-4.919, 1.397) |
|  | T3 × 2 children | 0.792 | .385 | (-0.994, 2.578) |
|  | T3 × 3+ children | 0.046 | .974 | (-2.713, 2.804) |
| Parental Mental Health: Total | T1 × 2 children | -0.849 | .159 | (-2.030, 0.332) |
|  | T1 × 3+ children | -0.921 | .318 | (-2.728, 0.886) |
|  | T2 × 2 children | -1.428 | .013 | (-2.558, -0.299) |
|  | T2× 3+ children | 0.576 | .676 | (-2.127, 3.278) |
|  | T3 × 2 children | -0.874 | .303 | (-2.539, 0.790) |
|  | T3 × 3+ children | -0.142 | .909 | (-2.561, 2.278) |
| Parental Mental Health: Depression | T1 × 2 children | -0.490 | .206 | (-1.248, 0.269) |
|  | T1 × 3+ children | -0.418 | .442 | (-1.485, 0.648) |
|  | T2 × 2 children | -0.873 | .012 | (-1.554, -0.192) |
|  | T2× 3+ children | 0.138 | .858 | (-1.375, 1.651) |
|  | T3 × 2 children | -0.733 | .185 | (-1.818, 0.351) |
|  | T3 × 3+ children | -1.081 | .097 | (-2.356, 0.195) |
| Parental Mental Health: Anxiety | T1 × 2 children | -0.375 | .273 | (-1.045, 0.296) |
|  | T1 × 3+ children | -0.509 | .398 | (-1.688, 0.671) |
|  | T2 × 2 children | -0.564 | .088 | (-1.211, 0.083) |
|  | T2× 3+ children | 0.403 | .570 | (-0.988, 1.794) |
|  | T3 × 2 children | -0.164 | .687 | (-0.961, 0.633) |
|  | T3 × 3+ children | 0.908 | .224 | (-0.556, 2.372) |
| Parenting Stress | T1 × 2 children | -0.463 | .689 | (-2.728, 1.801) |
|  | T1 × 3+ children | -2.623 | .256 | (-7.146, 1.900) |
|  | T2 × 2 children | -2.904 | .040 | (-5.681, -0.128) |
|  | T2× 3+ children | -1.997 | .604 | (-9.549, 5.556) |
|  | T3 × 2 children | 0.842 | .524 | (-1.748, 3.432) |
|  | T3 × 3+ children | 1.760 | .438 | (-2.692, 6.211) |
| Family Functioning | T1 × 2 children | -0.079 | .817 | (-0.749, 0.591) |
|  | T1 × 3+ children | 0.333 | .581 | (-0.849, 1.516) |
|  | T2 × 2 children | -0.392 | .288 | (-1.114, 0.331) |
|  | T2× 3+ children | 0.856 | .136 | (-0.270, 1.981) |
|  | T3 × 2 children | -0.097 | .818 | (-0.924, 0.730) |
|  | T3 × 3+ children | 0.736 | .360 | (-0.839, 2.311) |

**Note:** All models were adjusted for caregiver age, child age, marital status, caregiver gender, child gender, educational attainment, ethnicity, hukou status, employment status, and child disability. Total caregiver-perpetrated violence was estimated using mixed-effects negative binomial regression, whereas physical caregiver-perpetrated violence and emotional caregiver-perpetrated violence were estimated using mixed-effects Poisson regression and are reported as incidence rate ratios (IRRs). Other outcomes were estimated using linear mixed-effects regression.

## Table S17 Estimated Marginal Means for Post-Intervention Trajectories by Number of Children Within the Intervention Group (N = 806)

| Outcomes | Number of children | T0 (95% CI) | T1 (95% CI) | T2 (95% CI) | T3 (95% CI) |
| --- | --- | --- | --- | --- | --- |
| Early Learning and Stimulation | 1 child | 24.86 (22.41, 27.31) | 26.51 (23.99, 29.02) | 28.39 (25.52, 31.26) | 25.13 (22.36, 27.89) |
|  | 2 children | 21.59 (20.16, 23.03) | 25.07 (23.77, 26.38) | 29.09 (27.54, 30.64) | 24.46 (22.94, 25.97) |
|  | 3+ children | 19.77 (13.34, 26.21) | 25.95 (20.58, 31.31) | 29.05 (20.52, 37.59) | 24.15 (19.49, 28.80) |
| Caregiver-Perpetrated Violence: Emotional | 1 child | 7.88 (7.06, 8.69) | 2.95 (2.21, 3.69) | 5.69 (4.36, 7.03) | 4.02 (2.80, 5.25) |
|  | 2 children | 8.64 (8.08, 9.19) | 2.91 (2.48, 3.34) | 4.19 (3.53, 4.86) | 4.26 (3.45, 5.07) |
|  | 3+ children | 9.24 (6.66, 11.82) | 3.31 (-0.16, 6.79) | 6.36 (2.84, 9.88) | 2.44 (0.77, 4.11) |
| Caregiver-Perpetrated Violence: Physical | 1 child | 5.10 (4.54, 5.65) | 1.34 (0.80, 1.87) | 1.36 (0.84, 1.89) | 1.70 (0.89, 2.50) |
|  | 2 children | 5.57 (5.21, 5.94) | 1.32 (0.98, 1.67) | 1.47 (1.09, 1.84) | 1.36 (0.86, 1.85) |
|  | 3+ children | 6.28 (3.88, 8.68) | 1.60 (-0.04, 3.25) | 2.58 (0.95, 4.20) | 0.94 (-0.07, 1.96) |
| Caregiver-Perpetrated Violence: Total | 1 child | 14.43 (12.86, 16.00) | 4.16 (3.10, 5.23) | 6.62 (4.95, 8.28) | 5.20 (3.52, 6.89) |
|  | 2 children | 15.81 (14.69, 16.93) | 4.06 (3.42, 4.71) | 5.40 (4.54, 6.26) | 5.29 (4.29, 6.29) |
|  | 3+ children | 17.07 (12.16, 21.98) | 4.16 (0.53, 7.80) | 8.29 (2.58, 14.00) | 3.00 (0.73, 5.27) |
| Parental Mental Health: Anxiety | 1 child | 1.20 (0.66, 1.75) | 1.23 (0.73, 1.73) | 0.48 (0.11, 0.86) | 1.06 (0.38, 1.74) |
|  | 2 children | 1.93 (1.52, 2.34) | 1.59 (1.22, 1.97) | 0.80 (0.57, 1.03) | 1.54 (1.09, 2.00) |
|  | 3+ children | 1.66 (0.31, 3.01) | 0.86 (-0.05, 1.76) | 1.22 (0.08, 2.35) | 1.82 (0.60, 3.04) |
| Parental Mental Health: Depression | 1 child | 1.36 (0.77, 1.96) | 1.55 (0.91, 2.20) | 0.76 (0.33, 1.20) | 1.85 (0.80, 2.89) |
|  | 2 children | 2.25 (1.76, 2.73) | 1.96 (1.52, 2.40) | 0.82 (0.58, 1.05) | 1.89 (1.36, 2.43) |
|  | 3+ children | 1.23 (-0.05, 2.51) | 1.62 (0.04, 3.21) | 0.98 (-0.24, 2.21) | 0.64 (-0.35, 1.63) |
| Parental Mental Health: Total | 1 child | 2.56 (1.60, 3.53) | 2.77 (1.79, 3.76) | 1.24 (0.50, 1.98) | 2.88 (1.31, 4.45) |
|  | 2 children | 4.18 (3.36, 4.99) | 3.57 (2.84, 4.30) | 1.64 (1.20, 2.07) | 3.46 (2.54, 4.37) |
|  | 3+ children | 2.90 (0.40, 5.40) | 2.51 (0.45, 4.57) | 2.24 (0.03, 4.45) | 2.53 (0.72, 4.33) |
| Parenting Stress | 1 child | 36.53 (34.77, 38.29) | 36.99 (35.06, 38.92) | 36.65 (34.26, 39.03) | 40.16 (38.12, 42.19) |
|  | 2 children | 38.81 (37.73, 39.89) | 38.71 (37.59, 39.82) | 35.37 (34.04, 36.70) | 42.90 (41.61, 44.20) |
|  | 3+ children | 38.84 (33.52, 44.16) | 36.78 (32.63, 40.94) | 37.62 (28.46, 46.79) | 41.99 (37.34, 46.64) |
| Child Behavior: Total | 1 child | 11.84 (10.73, 12.96) | 9.35 (8.42, 10.28) | 9.05 (7.81, 10.28) | 9.08 (7.86, 10.31) |
|  | 2 children | 12.38 (11.79, 12.96) | 8.97 (8.37, 9.57) | 8.31 (7.67, 8.94) | 8.29 (7.53, 9.05) |
|  | 3+ children | 14.00 (11.27, 16.72) | 8.43 (6.42, 10.44) | 11.20 (7.64, 14.77) | 7.69 (4.77, 10.60) |
| Child Behavior: Internalizing | 1 child | 5.65 (5.19, 6.12) | 3.44 (2.91, 3.96) | 3.40 (2.88, 3.92) | 3.16 (2.60, 3.72) |
|  | 2 children | 5.74 (5.43, 6.05) | 3.02 (2.71, 3.34) | 3.02 (2.72, 3.32) | 2.85 (2.45, 3.24) |
|  | 3+ children | 6.24 (5.30, 7.17) | 2.65 (2.02, 3.28) | 3.39 (1.86, 4.92) | 2.72 (1.34, 4.10) |
| Child Behavior: Externalizing | 1 child | 6.19 (5.29, 7.10) | 5.93 (5.25, 6.60) | 5.64 (4.71, 6.57) | 5.92 (5.02, 6.83) |
|  | 2 children | 6.63 (6.20, 7.06) | 5.95 (5.53, 6.37) | 5.30 (4.82, 5.78) | 5.45 (4.93, 5.98) |
|  | 3+ children | 7.76 (5.50, 10.01) | 5.80 (3.96, 7.64) | 7.82 (5.31, 10.34) | 4.96 (2.83, 7.08) |
| Child Behavior: Emotional problem | 1 child | 3.80 (3.49, 4.10) | 1.65 (1.32, 1.98) | 1.71 (1.32, 2.09) | 1.53 (1.14, 1.91) |
|  | 2 children | 3.76 (3.54, 3.97) | 1.48 (1.26, 1.70) | 1.50 (1.28, 1.72) | 1.36 (1.11, 1.60) |
|  | 3+ children | 3.90 (3.04, 4.77) | 1.52 (1.03, 2.01) | 1.47 (0.51, 2.43) | 1.20 (0.19, 2.20) |
| Child Behavior: Conduct problem | 1 child | 2.12 (1.75, 2.50) | 1.96 (1.66, 2.26) | 1.98 (1.60, 2.36) | 1.78 (1.38, 2.17) |
|  | 2 children | 2.38 (2.18, 2.58) | 2.02 (1.83, 2.22) | 1.87 (1.66, 2.08) | 1.83 (1.59, 2.08) |
|  | 3+ children | 3.43 (2.19, 4.67) | 2.46 (1.62, 3.30) | 3.35 (1.99, 4.72) | 2.63 (1.63, 3.63) |
| Child Behavior: Hyperactivity | 1 child | 4.07 (3.41, 4.73) | 3.98 (3.40, 4.55) | 3.67 (2.99, 4.35) | 4.16 (3.46, 4.87) |
|  | 2 children | 4.25 (3.92, 4.58) | 3.93 (3.61, 4.25) | 3.42 (3.07, 3.78) | 3.62 (3.24, 4.00) |
|  | 3+ children | 4.32 (2.98, 5.66) | 3.34 (2.13, 4.56) | 4.45 (3.03, 5.88) | 2.26 (0.80, 3.71) |
| Child Behavior: Peer problem | 1 child | 1.85 (1.52, 2.19) | 1.78 (1.42, 2.15) | 1.68 (1.36, 2.00) | 1.61 (1.23, 2.00) |
|  | 2 children | 1.98 (1.77, 2.19) | 1.55 (1.37, 1.73) | 1.53 (1.33, 1.72) | 1.50 (1.25, 1.75) |
|  | 3+ children | 2.34 (1.42, 3.25) | 1.13 (0.54, 1.71) | 1.88 (0.75, 3.01) | 1.45 (0.71, 2.19) |
| Child Behavior: Prosocial behavior | 1 child | 6.96 (6.47, 7.46) | 7.37 (6.86, 7.88) | 7.24 (6.70, 7.78) | 7.61 (7.08, 8.14) |
|  | 2 children | 7.24 (6.96, 7.52) | 7.57 (7.28, 7.85) | 7.96 (7.67, 8.24) | 7.91 (7.60, 8.22) |
|  | 3+ children | 8.01 (6.98, 9.04) | 8.24 (7.24, 9.24) | 8.77 (7.99, 9.54) | 8.16 (7.29, 9.03) |
| Parenting Practices: Total | 1 child | 58.04 (56.12, 59.97) | 58.63 (56.70, 60.55) | 58.11 (55.69, 60.54) | 57.89 (55.39, 60.40) |
|  | 2 children | 55.97 (54.90, 57.04) | 57.19 (56.02, 58.36) | 58.18 (57.07, 59.29) | 57.16 (55.91, 58.41) |
|  | 3+ children | 57.16 (53.10, 61.22) | 58.87 (54.61, 63.13) | 55.74 (51.36, 60.11) | 61.02 (56.47, 65.57) |
| Parenting Practices: Positive | 1 child | 24.09 (23.28, 24.90) | 24.46 (23.58, 25.33) | 24.54 (23.53, 25.55) | 24.03 (22.95, 25.12) |
|  | 2 children | 23.97 (23.49, 24.45) | 24.22 (23.73, 24.72) | 24.66 (24.17, 25.16) | 23.95 (23.36, 24.53) |
|  | 3+ children | 24.08 (21.62, 26.54) | 25.29 (23.38, 27.19) | 24.52 (22.96, 26.08) | 25.73 (23.45, 28.00) |
| Parenting Practices: Involvement | 1 child | 33.95 (32.64, 35.26) | 34.17 (32.94, 35.39) | 33.57 (31.92, 35.21) | 33.87 (32.29, 35.46) |
|  | 2 children | 32.00 (31.27, 32.73) | 32.97 (32.20, 33.73) | 33.51 (32.77, 34.25) | 33.21 (32.42, 34.01) |
|  | 3+ children | 33.10 (30.38, 35.82) | 33.64 (30.74, 36.54) | 31.27 (28.01, 34.52) | 35.36 (32.59, 38.13) |
| Attitude towards Corporal Punishment | 1 child | 2.31 (1.96, 2.66) | 1.99 (1.67, 2.31) | 2.85 (2.48, 3.23) | 3.07 (2.69, 3.45) |
|  | 2 children | 2.63 (2.43, 2.82) | 2.19 (2.00, 2.38) | 2.93 (2.73, 3.14) | 3.07 (2.84, 3.30) |
|  | 3+ children | 2.51 (1.61, 3.41) | 2.42 (1.40, 3.45) | 3.43 (2.50, 4.37) | 3.20 (2.37, 4.03) |
| Family Functioning | 1 child | 2.70 (2.11, 3.29) | 3.15 (2.49, 3.82) | 2.65 (2.06, 3.24) | 2.88 (2.10, 3.67) |
|  | 2 children | 2.61 (2.25, 2.96) | 2.90 (2.52, 3.27) | 2.16 (1.78, 2.54) | 2.64 (2.18, 3.09) |
|  | 3+ children | 2.09 (0.77, 3.41) | 2.71 (1.28, 4.14) | 2.73 (1.24, 4.23) | 3.42 (1.51, 5.34) |

# References

1. Khan S, Hancioglu A. Multiple Indicator Cluster Surveys: Delivering Robust Data on Children and Women across the Globe. *Studies in Family Planning*. 2019;50(3):279-286. doi:10.1111/sifp.12103

2. Zou S, Zou X, Zhang R, et al. Maternal depression and early childhood development among children aged 24–59 months: the mediating effect of responsive caregiving. *Ann Gen Psychiatry*. 2024;23:30. doi:10.1186/s12991-024-00515-z

3. Meinck F, Boyes ME, Cluver L, et al. Adaptation and psychometric properties of the ISPCAN Child Abuse Screening Tool for use in trials (ICAST-Trial) among South African adolescents and their primary caregivers. *Child Abuse & Neglect*. 2018;82:45-58. doi:10.1016/j.chiabu.2018.05.022

4. Chen C, Wang X, Qin J, Huang Z. Psychometric testing of the Chinese version of ISPCAN Child Abuse Screening Tools Parent’s version (ICAST-P). *Children and Youth Services Review*. 2020;109(C). Accessed March 11, 2026. https://ideas.repec.org//a/eee/cysrev/v109y2020ics0190740919306383.html

5. Goodman R. The Strengths and Difficulties Questionnaire: A Research Note. *Journal of Child Psychology and Psychiatry*. 1997;38(5):581-586. doi:10.1111/j.1469-7610.1997.tb01545.x

6. Du Y, Kou J, Coghill D. The validity, reliability and normative scores of the parent, teacher and self report versions of the Strengths and Difficulties Questionnaire in China. *Child and Adolescent Psychiatry and Mental Health*. 2008;2(1):8. doi:10.1186/1753-2000-2-8

7. Gao X, Shi W, Zhai Y, He L, Shi X. Results of the parent-rated Strengths and Difficulties Questionnaire in 22,108 primary school students from 8 provinces of China. *Shanghai Arch Psychiatry*. 2013;25(6):364-374. doi:10.3969/j.issn.1002-0829.2013.06.005

8. Frick PJ. The Alabama parenting questionnaire. *University of Alabama*. Published online 1991.

9. Hsieh M. Reliability and validity of the Chinese version of the Short-form Alabama Parenting Questionnaire. *The Archive of Guidance & Counseling*. Published online 2020. Accessed March 11, 2026. https://psycnet.apa.org/record/2020-60643-003

10. Norton PJ. Depression Anxiety and Stress Scales (DASS-21): Psychometric analysis across four racial groups. *Anxiety, Stress & Coping*. 2007;20(3):253-265. doi:10.1080/10615800701309279

11. Oei TPS, Sawang S, Goh YW, Mukhtar F. Using the Depression Anxiety Stress Scale 21 (DASS-21) across cultures. *International Journal of Psychology*. 2013;48(6):1018-1029. doi:10.1080/00207594.2012.755535

12. Chan RCK, Xu T, Huang J, et al. Extending the utility of the Depression Anxiety Stress scale by examining its psychometric properties in Chinese settings. *Psychiatry Research*. 2012;200(2-3):879-883. doi:10.1016/j.psychres.2012.06.041

13. Gong X, Xie X, Xu R, Luo Y. Psychometric properties of the Chinese versions of DASS-21 in Chinese college students【抑郁-焦虑-压力量表简体中文版(DASS-21)在中国大学生中的测试报告】. *Chinese Journal of Clinical Psychology*. 2010;18(4):443-446.

14. Jiang LC, Yan YJ, Jin ZS, et al. The Depression Anxiety Stress Scale-21 in Chinese hospital workers: Reliability, latent structure, and measurement invariance across genders. *Frontiers in Psychology*. 2020;11. doi:10.3389/fpsyg.2020.00247

15. Berry JO, Jones WH. The Parental Stress Scale: Initial psychometric evidence. *Journal of Social and Personal Relationships*. 1995;(12):463-472. doi:10.1177/0265407595123009

16. Leung C, Tsang SKM. The Chinese Parental Stress Scale: Psychometric Evidence Using Rasch Modeling on Clinical and Nonclinical Samples. *Journal of Personality Assessment*. 2010;92(1):26-34. doi:10.1080/00223890903379209

17. Smilkstein G, Ashworth C, Montano D. Validity and reliability of the family APGAR as a test of family function. *Journal of Family Practice*. 1982;15(2):303-311.
