# Supplementary material for: Family Size and Longitudinal Outcomes of a Digital-Human Parenting Intervention in Chinese Preschool Families: Secondary Analysis
Source: J Med Internet Res. 2026 Jul 10;28:e101388. doi: 10.2196/101388 (PMC13354134; doi:10.2196/101388)
Supplement: Checklist 1 [file jmir-v28-e101388-s002.pdf]

CONSORT-EHEALTH (V 1.6.1) - Submission/Publication Form

The CONSORT-EHEALTH checklist is intended for authors of randomized trials evaluating web-based and Internet-based applications/interventions, including mobile interventions, electronic games (incl multiplayer games), social media, certain telehealth applications, and other interactive and/or networked electronic applications. Some of the items (e.g. all subitems under item 5 - description of the intervention) may also be applicable for other study designs.

The goal of the CONSORT EHEALTH checklist and guideline is to be
a) a guide for reporting for authors of RCTs,
b) to form a basis for appraisal of an ehealth trial (in terms of validity)

CONSORT-EHEALTH items/subitems are MANDATORY reporting items for studies published in the Journal of Medical Internet Research and other journals / scientific societies endorsing the checklist.

Items numbered 1., 2., 3., 4a., 4b etc are original CONSORT or CONSORT-NPT (non-pharmacologic treatment) items.
Items with Roman numerals (i., ii, iii, iv etc.) are CONSORT-EHEALTH extensions/clarifications.

As the CONSORT-EHEALTH checklist is still considered in a formative stage, we would ask that you also RATE ON A SCALE OF 1-5 how important/useful you feel each item is FOR THE PURPOSE OF THE CHECKLIST and reporting guideline (optional).

Mandatory reporting items are marked with a red *.
In the textboxes, either copy & paste the relevant sections from your manuscript into this form - please include any quotes from your manuscript in QUOTATION MARKS,
or answer directly by providing additional information not in the manuscript, or elaborating on why the item was not relevant for this study.

YOUR ANSWERS WILL BE PUBLISHED AS A SUPPLEMENTARY FILE TO YOUR PUBLICATION IN JMIR AND ARE CONSIDERED PART OF YOUR PUBLICATION (IF ACCEPTED).
Please fill in these questions diligently. Information will not be copyedited, so please use proper spelling and grammar, use correct capitalization, and avoid abbreviations.

DO NOT FORGET TO SAVE AS PDF _AND_ CLICK THE SUBMIT BUTTON SO YOUR ANSWERS ARE IN OUR DATABASE !!!

Citation Suggestion (if you append the pdf as Appendix we suggest to cite this paper in the caption):
Eysenbach G, CONSORT-EHEALTH Group
CONSORT-EHEALTH: Improving and Standardizing Evaluation Reports of Web-based and Mobile Health Interventions
J Med Internet Res 2011;13(4):e126
URL: http://www.jmir.org/2011/4/e126/
doi: 10.2196/jmir.1923
PMID: 22209829

sxy18739288787@gmail.com Switch account

Not shared

Draft saved

* Indicates required question

Your name *

First Last

Xinyu

Primary Affiliation (short), City, Country *

University of Toronto, Toronto, Canada

Peking University, Beijing, China

Your e-mail address *

abc@gmail.com

sxy18739288787@163.com

Title of your manuscript *

Provide the (draft) title of your manuscript.

Family Size and Longitudinal Outcomes of a Digital–Human Parenting Intervention in Chinese Preschool Families: Secondary Analysis of a Cluster Randomized Controlled Trial

Name of your App/Software/Intervention *

If there is a short and a long/alternate name, write the short name first and add the long name in brackets.

Keyushiguang

Evaluated Version (if any)

e.g. "V1", "Release 2017-03-01", "Version 2.0.27913"

CONSORT - EHEALTH checklist (V.1.6.1)

Language(s) *

What language is the intervention/app in? If multiple languages are available, separate by comma (e.g. "English, French")

Chinese

URL of your Intervention Website or App

e.g. a direct link to the mobile app on app in appstore (itunes, Google Play), or URL of the website. If the intervention is a DVD or hardware, you can also link to an Amazon page.

Your answer

URL of an image/screenshot (optional)

Your answer

Accessibility *

Can an enduser access the intervention presently?

- (selected) access is free and open
- access only for special usergroups, not open
- access is open to everyone, but requires payment/subscription/in-app purchases
- app/intervention no longer accessible
- Other:

Primary Medical Indication/Disease/Condition *

e.g. "Stress", "Diabetes", or define the target group in brackets after the condition, e.g. "Autism (Parents of children with)", "Alzheimers (Informal Caregivers of)"

Caregivers of children aged 3 to 6 years

Primary Outcomes measured in trial *

comma-separated list of primary outcomes reported in the trial

Early learning and stimulation, caregiver-perpe

Secondary/other outcomes

Are there any other outcomes the intervention is expected to affect?

Child behavior problems, parenting practices, parental mental health, parenting stress, family functioning, attitudes toward corporal punishment

Recommended "Dose" *

What do the instructions for users say on how often the app should be used?

- (•) Approximately Daily
- ( ) Approximately Weekly
- ( ) Approximately Monthly
- ( ) Approximately Yearly
- ( ) "as needed"
- ( ) Other:

Approx. Percentage of Users (starters) still using the app as recommended after 3 months *

- unknown / not evaluated
- 0-10%
- 11-20%
- 21-30%
- 31-40%
- 41-50%
- 51-60%
- 61-70%
- 71%-80%
- 81-90%
- 91-100%
- (selected) Other: The intervention itself lasted about 2.5 months, and parent can learn a

Overall, was the app/intervention effective? *

- ( ) yes: all primary outcomes were significantly better in intervention group vs control
- (•) partly: SOME primary outcomes were significantly better in intervention group vs control
- ( ) no statistically significant difference between control and intervention
- ( ) potentially harmful: control was significantly better than intervention in one or more outcomes
- ( ) inconclusive: more research is needed
- ( ) Other:

Article Preparation Status/Stage *

At which stage in your article preparation are you currently (at the time you fill in this form)

- ○ not submitted yet - in early draft status
- ○ not submitted yet - in late draft status, just before submission
- ◉ submitted to a journal but not reviewed yet
- ○ submitted to a journal and after receiving initial reviewer comments
- ○ submitted to a journal and accepted, but not published yet
- ○ published
- ○ Other:

Journal *

If you already know where you will submit this paper (or if it is already submitted), please provide the journal name (if it is not JMIR, provide the journal name under "other")

- ○ not submitted yet / unclear where I will submit this
- ○ Journal of Medical Internet Research (JMIR)
- ◉ JMIR mHealth and UHealth
- ○ JMIR Serious Games
- ○ JMIR Mental Health
- ○ JMIR Public Health
- ○ JMIR Formative Research
- ○ Other JMIR sister journal
- ○ Other:

Is this a full powered effectiveness trial or a pilot/feasibility trial? *

- ○ Pilot/feasibility
- ◉ Fully powered

Manuscript tracking number *

If this is a JMIR submission, please provide the manuscript tracking number under "other" (The ms tracking number can be found in the submission acknowledgement email, or when you login as author in JMIR. If the paper is already published in JMIR, then the ms tracking number is the four-digit number at the end of the DOI, to be found at the bottom of each published article in JMIR)

- ○ no ms number (yet) / not (yet) submitted to / published in JMIR
- ◉ Other: JMIR ms#101388

TITLE AND ABSTRACT

1a) TITLE: Identification as a randomized trial in the title

1a) Does your paper address CONSORT item 1a? *

I.e does the title contain the phrase "Randomized Controlled Trial"? (if not, explain the reason under "other")

- ◉ yes
- ○ Other:

1a-i) Identify the mode of delivery in the title

Identify the mode of delivery. Preferably use “web-based” and/or “mobile” and/or “electronic game” in the title. Avoid ambiguous terms like “online”, “virtual”, “interactive”. Use “Internet-based” only if Intervention includes non-web-based Internet components (e.g. email), use “computer-based” or “electronic” only if offline products are used. Use “virtual” only in the context of “virtual reality” (3-D worlds). Use “online” only in the context of “online support groups”. Complement or substitute product names with broader terms for the class of products (such as “mobile” or “smart phone” instead of “iphone”), especially if the application runs on different platforms.

	1	2	3	4	5	
subitem not at all important	○	○	○	○	◉	essential

Clear selection

Does your paper address subitem 1a-i? *

Copy and paste relevant sections from manuscript title (include quotes in quotation marks "like this" to indicate direct quotes from your manuscript), or elaborate on this item by providing additional information not in the ms, or briefly explain why the item is not applicable/relevant for your study

The manuscript title identifies the mode of delivery "a Digital–Human Parenting Intervention"

1a-ii) Non-web-based components or important co-interventions in title

Mention non-web-based components or important co-interventions in title, if any (e.g., “with telephone support”).

	1	2	3	4	5	
subitem not at all important	○	○	◉	○	○	essential

Clear selection

Does your paper address subitem 1a-ii?

Copy and paste relevant sections from manuscript title (include quotes in quotation marks "like this" to indicate direct quotes from your manuscript), or elaborate on this item by providing additional information not in the ms, or briefly explain why the item is not applicable/relevant for your study

Your answer

1a-iii) Primary condition or target group in the title

Mention primary condition or target group in the title, if any (e.g., “for children with Type I Diabetes”) Example: A Web-based and Mobile Intervention with Telephone Support for Children with Type I Diabetes: Randomized Controlled Trial

	1	2	3	4	5	
subitem not at all important	○	○	○	○	◉	essential

Clear selection

Does your paper address subitem 1a-iii? *

Copy and paste relevant sections from manuscript title (include quotes in quotation marks "like this" to indicate direct quotes from your manuscript), or elaborate on this item by providing additional information not in the ms, or briefly explain why the item is not applicable/relevant for your study

The title mentioned the target group is " Chinese Preschool Families"

1b) ABSTRACT: Structured summary of trial design, methods, results, and conclusions

NPT extension: Description of experimental treatment, comparator, care providers, centers, and blinding status.

1b-i) Key features/functionalities/components of the intervention and comparator in the METHODS section of the ABSTRACT

Mention key features/functionalities/components of the intervention and comparator in the abstract. If possible, also mention theories and principles used for designing the site. Keep in mind the needs of systematic reviewers and indexers by including important synonyms. (Note: Only report in the abstract what the main paper is reporting. If this information is missing from the main body of text, consider adding it)

	1	2	3	4	5	
subitem not at all important	○	○	○	○	◉	essential

Clear selection

Does your paper address subitem 1b-i? *

Copy and paste relevant sections from the manuscript abstract (include quotes in quotation marks "like this" to indicate direct quotes from your manuscript), or elaborate on this item by providing additional information not in the ms, or briefly explain why the item is not applicable/relevant for your study

The abstract Methods section describes the intervention as "a universal digital-human parenting intervention delivered through the preschool system in China (N = 541)." This identifies the intervention as universal, digital-human, and preschool-system-delivered, but the abstract does not describe specific intervention components or the comparator.

1b-ii) Level of human involvement in the METHODS section of the ABSTRACT

Clarify the level of human involvement in the abstract, e.g., use phrases like “fully automated” vs. “therapist/nurse/care provider/physician-assisted” (mention number and expertise of providers involved, if any). (Note: Only report in the abstract what the main paper is reporting. If this information is missing from the main body of text, consider adding it)

	1	2	3	4	5	
subitem not at all important	○	○	◉	○	○	essential

Clear selection

Does your paper address subitem 1b-ii?

Copy and paste relevant sections from the manuscript abstract (include quotes in quotation marks "like this" to indicate direct quotes from your manuscript), or elaborate on this item by providing additional information not in the ms, or briefly explain why the item is not applicable/relevant for your study

Your answer

1b-iii) Open vs. closed, web-based (self-assessment) vs. face-to-face assessments in the METHODS section of the ABSTRACT

Mention how participants were recruited (online vs. offline), e.g., from an open access website or from a clinic or a closed online user group (closed usergroup trial), and clarify if this was a purely web-based trial, or there were face-to-face components (as part of the intervention or for assessment). Clearly say if outcomes were self-assessed through questionnaires (as common in web-based trials). Note: In traditional offline trials, an open trial (open-label trial) is a type of clinical trial in which both the researchers and participants know which treatment is being administered. To avoid confusion, use “blinded” or “unblinded” to indicated the level of blinding instead of “open”, as “open” in web-based trials usually refers to “open access” (i.e. participants can self-enrol). (Note: Only report in the abstract what the main paper is reporting. If this information is missing from the main body of text, consider adding it)

	1	2	3	4	5	
subitem not at all important	○	○	◉	○	○	essential

Clear selection

Does your paper address subitem 1b-iii?

Copy and paste relevant sections from the manuscript abstract (include quotes in quotation marks "like this" to indicate direct quotes from your manuscript), or elaborate on this item by providing additional information not in the ms, or briefly explain why the item is not applicable/relevant for your study

Your answer

1b-iv) RESULTS section in abstract must contain use data

Report number of participants enrolled/assessed in each group, the use/uptake of the intervention (e.g., attrition/adherence metrics, use over time, number of logins etc.), in addition to primary/secondary outcomes. (Note: Only report in the abstract what the main paper is reporting. If this information is missing from the main body of text, consider adding it)

	1	2	3	4	5	
subitem not at all important	○	○	◉	○	○	essential

Clear selection

Does your paper address subitem 1b-iv?

Copy and paste relevant sections from the manuscript abstract (include quotes in quotation marks "like this" to indicate direct quotes from your manuscript), or elaborate on this item by providing additional information not in the ms, or briefly explain why the item is not applicable/relevant for your study

The abstract Results section reports outcome findings but does not include intervention use/uptake data. The abstract states: "The number of children did not significantly moderate intervention effectiveness at immediate post-intervention. However, trajectories diverged over time."

1b-v) CONCLUSIONS/DISCUSSION in abstract for negative trials

Conclusions/Discussions in abstract for negative trials: Discuss the primary outcome - if the trial is negative (primary outcome not changed), and the intervention was not used, discuss whether negative results are attributable to lack of uptake and discuss reasons. (Note: Only report in the abstract what the main paper is reporting. If this information is missing from the main body of text, consider adding it)

	1	2	3	4	5	
subitem not at all important	○	○	◉	○	○	essential

Clear selection

Does your paper address subitem 1b-v?

Copy and paste relevant sections from the manuscript abstract (include quotes in quotation marks "like this" to indicate direct quotes from your manuscript), or elaborate on this item by providing additional information not in the ms, or briefly explain why the item is not applicable/relevant for your study

The abstract conclusion states: "Family size might not always be associated with short-term intervention effectiveness but was associated with divergence in longer-term trajectories."

INTRODUCTION

2a) In INTRODUCTION: Scientific background and explanation of rationale

2a-i) Problem and the type of system/solution

Describe the problem and the type of system/solution that is object of the study: intended as stand-alone intervention vs. incorporated in broader health care program? Intended for a particular patient population? Goals of the intervention, e.g., being more cost-effective to other interventions, replace or complement other solutions? (Note: Details about the intervention are provided in "Methods" under 5)

	1	2	3	4	5	
subitem not at all important	○	○	○	○	◉	essential

Clear selection

Does your paper address subitem 2a-i? *

Copy and paste relevant sections from the manuscript (include quotes in quotation marks "like this" to indicate direct quotes from your manuscript), or elaborate on this item by providing additional information not in the ms, or briefly explain why the item is not applicable/relevant for your study

problem/gap: "However, less is known about how intervention effects vary by family size, particularly the number of children within the household"; system/solution: "a pragmatic, two-arm, single-blind cluster randomized controlled trial evaluating a universal digital-human parenting intervention delivered through the preschool system in China"; intervention: "The intervention was a universal digital-human parenting program, named 'Keyushiguang', delivered through the preschool system."; target population: "Eligible participants were primary caregivers of children aged 3 to 6 years enrolled in the preschool."

2a-ii) Scientific background, rationale: What is known about the (type of) system

Scientific background, rationale: What is known about the (type of) system that is the object of the study (be sure to discuss the use of similar systems for other conditions/diagnoses, if appropiate), motivation for the study, i.e. what are the reasons for and what is the context for this specific study, from which stakeholder viewpoint is the study performed, potential impact of findings [2]. Briefly justify the choice of the comparator.

	1	2	3	4	5	
subitem not at all important	○	○	○	○	◉	essential

Clear selection

Does your paper address subitem 2a-ii? *

Copy and paste relevant sections from the manuscript (include quotes in quotation marks "like this" to indicate direct quotes from your manuscript), or elaborate on this item by providing additional information not in the ms, or briefly explain why the item is not applicable/relevant for your study

background: "Parenting interventions are widely recognized as effective strategies for improving parenting practices and child developmental outcomes."; rationale/gap: "Family size is typically included as control variables rather than examined as sources of variation in intervention processes and outcomes"; prior evidence: "Meta-analytic findings suggest that larger families may show smaller improvements in child externalizing behaviors"; objectives: "The present study aims to examine whether family size... is associated with (1) differences in baseline parental and child outcomes, (2) variation in intervention effectiveness at immediate post-intervention, and (3) differences in impact trajectories over 6- and 12-month follow-up periods."; comparator: "the waitlist control group received no formal support during this period".

2b) In INTRODUCTION: Specific objectives or hypotheses

Does your paper address CONSORT subitem 2b? *

Copy and paste relevant sections from the manuscript (include quotes in quotation marks "like this" to indicate direct quotes from your manuscript), or elaborate on this item by providing additional information not in the ms, or briefly explain why the item is not applicable/relevant for your study

The manuscript states the specific objectives in the Introduction:
"The present study aims to examine whether family size, particularly with respect to the number of children, is associated with (1) differences in baseline parental and child outcomes, (2) variation in intervention effectiveness at immediate post-intervention, and (3) differences in impact trajectories over 6- and 12-month follow-up periods."

The manuscript further explains the contribution of these objectives:
"Findings of this study contribute to the literature by examining family size as a source of variation in caregiving contexts rather than solely as a control variable, and by integrating baseline heterogeneity with both short-term intervention effects and longer-term trajectories."

METHODS

3a) Description of trial design (such as parallel, factorial) including allocation ratio

Does your paper address CONSORT subitem 3a? *

Copy and paste relevant sections from the manuscript (include quotes in quotation marks "like this" to indicate direct quotes from your manuscript), or elaborate on this item by providing additional information not in the ms, or briefly explain why the item is not applicable/relevant for your study

The manuscript describes the trial design:
"The present study is a secondary analysis of data from a pragmatic, two-arm, single-blind cluster randomized controlled trial evaluating a universal digital-human parenting intervention delivered through the preschool system in China."

The manuscript reports the allocation ratio and trial arms:
"All 21 classes within the preschool were included as clusters and randomly assigned in a 1:1 ratio to either the intervention group (10 classes) or a waitlist control group (11 classes) using a computer-generated sequence by an independent researcher."

The manuscript reports the number of participants in each group:
"A total of 541 caregivers were enrolled, including 272 in the intervention group and 269 in the control group."

3b) Important changes to methods after trial commencement (such as eligibility criteria), with reasons

Does your paper address CONSORT subitem 3b? *

Copy and paste relevant sections from the manuscript (include quotes in quotation marks "like this" to indicate direct quotes from your manuscript), or elaborate on this item by providing additional information not in the ms, or briefly explain why the item is not applicable/relevant for your study

No relevant manuscript statement was identified reporting important changes to methods after trial commencement

3b-i) Bug fixes, Downtimes, Content Changes

Bug fixes, Downtimes, Content Changes: ehealth systems are often dynamic systems. A description of changes to methods therefore also includes important changes made on the intervention or comparator during the trial (e.g., major bug fixes or changes in the functionality or content) (5-iii) and other “unexpected events” that may have influenced study design such as staff changes, system failures/downtimes, etc. [2].

	1	2	3	4	5	
subitem not at all important	(●)	○	○	○	○	essential

Clear selection

Does your paper address subitem 3b-i?

Copy and paste relevant sections from the manuscript (include quotes in quotation marks "like this" to indicate direct quotes from your manuscript), or elaborate on this item by providing additional information not in the ms, or briefly explain why the item is not applicable/relevant for your study

Your answer

4a) Eligibility criteria for participants

Does your paper address CONSORT subitem 4a? *

Copy and paste relevant sections from the manuscript (include quotes in quotation marks "like this" to indicate direct quotes from your manuscript), or elaborate on this item by providing additional information not in the ms, or briefly explain why the item is not applicable/relevant for your study

The manuscript identifies the eligible participant group:
"Eligible participants were primary caregivers of children aged 3 to 6 years enrolled in the preschool."

The manuscript reports the inclusion criteria:
"Inclusion criteria were minimal to reflect the universal nature of the intervention: caregivers had to be aged 18 years or older, have access to a smartphone, and provide informed consent."

The manuscript reports that no additional exclusion criteria were applied:
"No additional exclusion criteria were applied."

4a-i) Computer / Internet literacy

Computer / Internet literacy is often an implicit “de facto” eligibility criterion - this should be explicitly clarified.

	1	2	3	4	5	
subitem not at all important	(selected)	○	○	○	○	essential

Clear selection

Does your paper address subitem 4a-i?

Copy and paste relevant sections from the manuscript (include quotes in quotation marks "like this" to indicate direct quotes from your manuscript), or elaborate on this item by providing additional information not in the ms, or briefly explain why the item is not applicable/relevant for your study

Your answer

4a-ii) Open vs. closed, web-based vs. face-to-face assessments:

Open vs. closed, web-based vs. face-to-face assessments: Mention how participants were recruited (online vs. offline), e.g., from an open access website or from a clinic, and clarify if this was a purely web-based trial, or there were face-to-face components (as part of the intervention or for assessment), i.e., to what degree got the study team to know the participant. In online-only trials, clarify if participants were quasi-anonymous and whether having multiple identities was possible or whether technical or logistical measures (e.g., cookies, email confirmation, phone calls) were used to detect/prevent these.

	1	2	3	4	5	
subitem not at all important	○	○	○	●	○	essential

Clear selection

Does your paper address subitem 4a-ii? *

Copy and paste relevant sections from the manuscript (include quotes in quotation marks "like this" to indicate direct quotes from your manuscript), or elaborate on this item by providing additional information not in the ms, or briefly explain why the item is not applicable/relevant for your study

recruitment channel: "Caregivers were recruited through routine school communication channels, with one caregiver participating per family."; setting: "The trial was conducted in a large public preschool in a lower-middle-income city in Jiangxi Province, central China."; cluster structure: "All 21 classes within the preschool were included as clusters"; delivery context: "The intervention was a universal digital-human parenting program, named 'Keyushiguang', delivered through the preschool system."

4a-iii) Information giving during recruitment

Information given during recruitment. Specify how participants were briefed for recruitment and in the informed consent procedures (e.g., publish the informed consent documentation as appendix, see also item X26), as this information may have an effect on user self-selection, user expectation and may also bias results.

	1	2	3	4	5	
subitem not at all important	(●)	( )	( )	( )	( )	essential

Clear selection

Does your paper address subitem 4a-iii?

Copy and paste relevant sections from the manuscript (include quotes in quotation marks "like this" to indicate direct quotes from your manuscript), or elaborate on this item by providing additional information not in the ms, or briefly explain why the item is not applicable/relevant for your study

The manuscript indicates that informed consent was obtained before data collection:
"All participants provided informed consent prior to data collection."

4b) Settings and locations where the data were collected

Does your paper address CONSORT subitem 4b? *

Copy and paste relevant sections from the manuscript (include quotes in quotation marks "like this" to indicate direct quotes from your manuscript), or elaborate on this item by providing additional information not in the ms, or briefly explain why the item is not applicable/relevant for your study

The manuscript identifies the study setting and geographic location:
"The trial was conducted in a large public preschool in a lower-middle-income city in Jiangxi Province, central China."

The manuscript describes the preschool/class-based trial setting:
"All 21 classes within the preschool were included as clusters and randomly assigned in a 1:1 ratio to either the intervention group (10 classes) or a waitlist control group (11 classes) using a computer-generated sequence by an independent researcher."

The manuscript reports the timing of data collection:
"Data were collected at four time points: baseline (T0), immediate post-intervention (T1), 6-month follow-up (T2), and 12-month follow-up (T3

4b-i) Report if outcomes were (self-)assessed through online questionnaires

Clearly report if outcomes were (self-)assessed through online questionnaires (as common in web-based trials) or otherwise.

	1	2	3	4	5	
subitem not at all important	○	○	○	◉	○	essential

Clear selection

Does your paper address subitem 4b-i? *

Copy and paste relevant sections from the manuscript (include quotes in quotation marks "like this" to indicate direct quotes from your manuscript), or elaborate on this item by providing additional information not in the ms, or briefly explain why the item is not applicable/relevant for your study

The manuscript states that outcomes were caregiver self-reported:
"All outcomes were based on caregiver self-report using validated instruments with established psychometric properties in Chinese populations."

The manuscript reports the assessment time points:
"Data were collected at four time points: baseline (T0), immediate post-intervention (T1), 6-month follow-up (T2), and 12-month follow-up (T3)."

4b-ii) Report how institutional affiliations are displayed

Report how institutional affiliations are displayed to potential participants [on ehealth media], as affiliations with prestigious hospitals or universities may affect volunteer rates, use, and reactions with regards to an intervention.(Not a required item – describe only if this may bias results)

	1	2	3	4	5	
subitem not at all important	(●)	( )	( )	( )	( )	essential

Clear selection

Does your paper address subitem 4b-ii?

Copy and paste relevant sections from the manuscript (include quotes in quotation marks "like this" to indicate direct quotes from your manuscript), or elaborate on this item by providing additional information not in the ms, or briefly explain why the item is not applicable/relevant for your study

Your answer

5) The interventions for each group with sufficient details to allow replication, including how and when they were actually administered

5-i) Mention names, credential, affiliations of the developers, sponsors, and owners

Mention names, credential, affiliations of the developers, sponsors, and owners [6] (if authors/evaluators are owners or developer of the software, this needs to be declared in a "Conflict of interest" section or mentioned elsewhere in the manuscript).

	1	2	3	4	5	
subitem not at all important	○	○	○	○	◉	essential

Clear selection

Does your paper address subitem 5-i?

Copy and paste relevant sections from the manuscript (include quotes in quotation marks "like this" to indicate direct quotes from your manuscript), or elaborate on this item by providing additional information not in the ms, or briefly explain why the item is not applicable/relevant for your study

intervention/program basis: "The intervention was a universal digital-human parenting program, named 'Keyushiguang', delivered through the preschool system."; origin: "It was based on the Parenting for Lifelong Health (PLH) programme... and adapted from the PLH ParentText intervention... for delivery via WeChat."; developer/owner relationship: "JML is the CEO of Parenting for Lifelong Health (PLH), a charitable organization based in the United Kingdom that developed the adapted program."; licensing: "PLH programs are open access and licensed under a Creative Commons 4.0 Attribution Share-Alike license."; funding: "China Postdoctoral Science Foundation (2022M720467), Peking University Start-Up Research Grant, LEGO Foundation".

5-ii) Describe the history/development process

Describe the history/development process of the application and previous formative evaluations (e.g., focus groups, usability testing), as these will have an impact on adoption/use rates and help with interpreting results.

	1	2	3	4	5	
subitem not at all important	○	○	○	○	◉	essential

Clear selection

Does your paper address subitem 5-ii?

Copy and paste relevant sections from the manuscript (include quotes in quotation marks "like this" to indicate direct quotes from your manuscript), or elaborate on this item by providing additional information not in the ms, or briefly explain why the item is not applicable/relevant for your study

The manuscript describes the intervention's program origin and adaptation history:
"The intervention was a universal digital-human parenting program, named 'Keyushiguang', delivered through the preschool system."

"It was based on the Parenting for Lifelong Health (PLH) programme (cite PLH) and adapted from the PLH ParentText intervention (cite ParentText) for delivery via WeChat."

5-iii) Revisions and updating

Revisions and updating. Clearly mention the date and/or version number of the application/intervention (and comparator, if applicable) evaluated, or describe whether the intervention underwent major changes during the evaluation process, or whether the development and/or content was "frozen" during the trial. Describe dynamic components such as news feeds or changing content which may have an impact on the replicability of the intervention (for unexpected events see item 3b).

	1	2	3	4	5	
subitem not at all important	(●)	( )	( )	( )	( )	essential

Clear selection

Does your paper address subitem 5-iii?

Copy and paste relevant sections from the manuscript (include quotes in quotation marks "like this" to indicate direct quotes from your manuscript), or elaborate on this item by providing additional information not in the ms, or briefly explain why the item is not applicable/relevant for your study

Your answer

5-iv) Quality assurance methods

Provide information on quality assurance methods to ensure accuracy and quality of information provided [1], if applicable.

	1	2	3	4	5	
subitem not at all important	(●)	( )	( )	( )	( )	essential

Clear selection

Does your paper address subitem 5-iv?

Copy and paste relevant sections from the manuscript (include quotes in quotation marks "like this" to indicate direct quotes from your manuscript), or elaborate on this item by providing additional information not in the ms, or briefly explain why the item is not applicable/relevant for your study

Your answer

5-v) Ensure replicability by publishing the source code, and/or providing screenshots/screen-capture video, and/or providing flowcharts of the algorithms used

Ensure replicability by publishing the source code, and/or providing screenshots/screen-capture video, and/or providing flowcharts of the algorithms used. Replicability (i.e., other researchers should in principle be able to replicate the study) is a hallmark of scientific reporting.

	1	2	3	4	5	
subitem not at all important	○	○	○	○	◉	essential

Clear selection

Does your paper address subitem 5-v?

Copy and paste relevant sections from the manuscript (include quotes in quotation marks "like this" to indicate direct quotes from your manuscript), or elaborate on this item by providing additional information not in the ms, or briefly explain why the item is not applicable/relevant for your study

"Further details about the intervention are provided in related publications(Zuyi et al.,2026)

5-vi) Digital preservation

Digital preservation: Provide the URL of the application, but as the intervention is likely to change or disappear over the course of the years; also make sure the intervention is archived (Internet Archive, webcitation.org, and/or publishing the source code or screenshots/videos alongside the article). As pages behind login screens cannot be archived, consider creating demo pages which are accessible without login.

	1	2	3	4	5	
subitem not at all important	(•)	( )	( )	( )	( )	essential

Clear selection

Does your paper address subitem 5-vi?

Copy and paste relevant sections from the manuscript (include quotes in quotation marks "like this" to indicate direct quotes from your manuscript), or elaborate on this item by providing additional information not in the ms, or briefly explain why the item is not applicable/relevant for your study

Your answer

5-vii) Access

Access: Describe how participants accessed the application, in what setting/context, if they had to pay (or were paid) or not, whether they had to be a member of specific group. If known, describe how participants obtained "access to the platform and Internet" [1]. To ensure access for editors/reviewers/readers, consider to provide a "backdoor" login account or demo mode for reviewers/readers to explore the application (also important for archiving purposes, see vi).

	1	2	3	4	5	
subitem not at all important	○	○	○	○	◉	essential

Clear selection

Does your paper address subitem 5-vii? *

Copy and paste relevant sections from the manuscript (include quotes in quotation marks "like this" to indicate direct quotes from your manuscript), or elaborate on this item by providing additional information not in the ms, or briefly explain why the item is not applicable/relevant for your study

access/device requirement: "caregivers had to be aged 18 years or older, have access to a smartphone, and provide informed consent."; recruitment/access context: "Caregivers were recruited through routine school communication channels"; delivery context: "The intervention was a universal digital-human parenting program, named 'Keyushiguang', delivered through the preschool system."; platform: "adapted from the PLH ParentText intervention... for delivery via WeChat."; group-based access/support: "weekly or twice-weekly message-based group discussions... within class-based WeChat groups".

5-viii) Mode of delivery, features/functionalities/components of the intervention and comparator, and the theoretical framework

Describe mode of delivery, features/functionalities/components of the intervention and comparator, and the theoretical framework [6] used to design them (instructional strategy [1], behaviour change techniques, persuasive features, etc., see e.g., [7, 8] for terminology). This includes an in-depth description of the content (including where it is coming from and who developed it) [1]," whether [and how] it is tailored to individual circumstances and allows users to track their progress and receive feedback" [6]. This also includes a description of communication delivery channels and – if computer-mediated communication is a component – whether communication was synchronous or asynchronous [6]. It also includes information on presentation strategies [1], including page design principles, average amount of text on pages, presence of hyperlinks to other resources, etc. [1].

	1	2	3	4	5	
subitem not at all important	○	○	○	○	◉	essential

Clear selection

Does your paper address subitem 5-viii? *

Copy and paste relevant sections from the manuscript (include quotes in quotation marks "like this" to indicate direct quotes from your manuscript), or elaborate on this item by providing additional information not in the ms, or briefly explain why the item is not applicable/relevant for your study

mode/platform: "The intervention was a universal digital-human parenting program, named 'Keyushiguang', delivered through the preschool system."; origin: "based on the Parenting for Lifelong Health (PLH) programme... and adapted from the PLH ParentText intervention... for delivery via WeChat."; components/content: "37-39 (depending on child age) brief interactive chatbot modules delivered over approximately 2.5 months"; tailoring: "Content was delivered daily in a self-directed format and tailored to caregiver and child characteristics."; human support: "weekly or twice-weekly message-based group discussions... within class-based WeChat groups"; engagement/comparator: "facilitator reminders and a child-centered incentive mechanism"; "waitlist control group received no formal support".

5-ix) Describe use parameters

Describe use parameters (e.g., intended "doses" and optimal timing for use). Clarify what instructions or recommendations were given to the user, e.g., regarding timing, frequency, heaviness of use, if any, or was the intervention used ad libitum.

	1	2	3	4	5	
subitem not at all important	○	○	○	◉	○	essential

Clear selection

Does your paper address subitem 5-ix?

Copy and paste relevant sections from the manuscript (include quotes in quotation marks "like this" to indicate direct quotes from your manuscript), or elaborate on this item by providing additional information not in the ms, or briefly explain why the item is not applicable/relevant for your study

intended dose/duration: "The program comprised 37-39 (depending on child age) brief interactive chatbot modules delivered over approximately 2.5 months"; timing/frequency: "Content was delivered daily in a self-directed format and tailored to caregiver and child characteristics."; human-support frequency: "weekly or twice-weekly message-based group discussions"; engagement support: "Additional strategies included facilitator reminders and a child-centered incentive mechanism linking caregiver participation to small rewards for children."

5-x) Clarify the level of human involvement

Clarify the level of human involvement (care providers or health professionals, also technical assistance) in the e-intervention or as co-intervention (detail number and expertise of professionals involved, if any, as well as “type of assistance offered, the timing and frequency of the support, how it is initiated, and the medium by which the assistance is delivered”. It may be necessary to distinguish between the level of human involvement required for the trial, and the level of human involvement required for a routine application outside of a RCT setting (discuss under item 21 – generalizability).

	1	2	3	4	5	
subitem not at all important	○	○	◉	○	○	essential

Clear selection

Does your paper address subitem 5-x?

Copy and paste relevant sections from the manuscript (include quotes in quotation marks "like this" to indicate direct quotes from your manuscript), or elaborate on this item by providing additional information not in the ms, or briefly explain why the item is not applicable/relevant for your study

The manuscript describes the human-support component:
"The digital content was complemented by human support, including weekly or twice-weekly message-based group discussions facilitated by preschool headteachers and social workers within class-based WeChat groups, to reinforce key messages, support reflection, and encourage peer interaction."

5-xi) Report any prompts/reminders used

Report any prompts/reminders used: Clarify if there were prompts (letters, emails, phone calls, SMS) to use the application, what triggered them, frequency etc. It may be necessary to distinguish between the level of prompts/reminders required for the trial, and the level of prompts/reminders for a routine application outside of a RCT setting (discuss under item 21 – generalizability).

	1	2	3	4	5	
subitem not at all important	○	○	○	●	○	essential

Clear selection

Does your paper address subitem 5-xi? *

Copy and paste relevant sections from the manuscript (include quotes in quotation marks "like this" to indicate direct quotes from your manuscript), or elaborate on this item by providing additional information not in the ms, or briefly explain why the item is not applicable/relevant for your study

The manuscript reports that facilitator reminders were used:
"Additional strategies included facilitator reminders and a child-centered incentive mechanism linking caregiver participation to small rewards for children."

The manuscript also describes the WeChat-based human-support setting in which reminders were likely delivered:
"The digital content was complemented by human support, including weekly or twice-weekly message-based group discussions facilitated by preschool headteachers and social workers within class-based WeChat groups, to reinforce key messages, support reflection, and encourage peer interaction."

5-xii) Describe any co-interventions (incl. training/support)

Describe any co-interventions (incl. training/support): Clearly state any interventions that are provided in addition to the targeted eHealth intervention, as ehealth intervention may not be designed as stand-alone intervention. This includes training sessions and support [1]. It may be necessary to distinguish between the level of training required for the trial, and the level of training for a routine application outside of a RCT setting (discuss under item 21 – generalizability.

	1	2	3	4	5	
subitem not at all important	○	○	○	◉	○	essential

Clear selection

Does your paper address subitem 5-xii? *

Copy and paste relevant sections from the manuscript (include quotes in quotation marks "like this" to indicate direct quotes from your manuscript), or elaborate on this item by providing additional information not in the ms, or briefly explain why the item is not applicable/relevant for your study

The manuscript describes additional engagement strategies provided alongside the digital intervention:
"Additional strategies included facilitator reminders and a child-centered incentive mechanism linking caregiver participation to small rewards for children."
The manuscript describes human support provided in addition to the digital content:
"The digital content was complemented by human support, including weekly or twice-weekly message-based group discussions facilitated by preschool headteachers and social workers within class-based WeChat groups, to reinforce key messages, support reflection, and encourage peer interaction."

6a) Completely defined pre-specified primary and secondary outcome measures, including how and when they were assessed

Does your paper address CONSORT subitem 6a? *

Copy and paste relevant sections from the manuscript (include quotes in quotation marks "like this" to indicate direct quotes from your manuscript), or elaborate on this item by providing additional information not in the ms, or briefly explain why the item is not applicable/relevant for your study

primary outcomes: "Primary outcomes included early learning and stimulation, assessed using items from the Multiple Indicator Cluster Surveys (MICS), and caregiver-perpetrated violence, measured using the ISPCAN Child Abuse Screening Tool (ICAST)."; secondary outcomes: "Secondary outcomes captured broader domains of parenting and child functioning, including child behavior problems... parenting practices... parental mental health... parenting stress... and family functioning"; additional outcome: "Attitudes toward corporal punishment were assessed using a single item from MICS."; assessment mode/time: "All outcomes were based on caregiver self-report"; "Data were collected at four time points: baseline (T0), immediate post-intervention (T1), 6-month follow-up (T2), and 12-month follow-up (T3)."

6a-i) Online questionnaires: describe if they were validated for online use and apply CHERRIES items to describe how the questionnaires were designed/deployed

If outcomes were obtained through online questionnaires, describe if they were validated for online use and apply CHERRIES items to describe how the questionnaires were designed/deployed [9].

	1	2	3	4	5	
subitem not at all important	○	○	○	○	◉	essential

Clear selection

Does your paper address subitem 6a-i?

Copy and paste relevant sections from manuscript text

self-report/validated instruments: "All outcomes were based on caregiver self-report using validated instruments with established psychometric properties in Chinese populations."; measure detail: "Detailed descriptions of all measures and their psychometric properties are provided in Appendix 2."; validation examples: "These items are embedded within the MICS Early Childhood Development framework (ECDI2030), which has been validated in Chinese populations"; "The instrument has been validated in mainland Chinese samples, with confirmatory factor analysis supporting its multidimensional structure".

6a-ii) Describe whether and how “use” (including intensity of use/dosage) was defined/measured/monitored

Describe whether and how “use” (including intensity of use/dosage) was defined/measured/monitored (logins, logfile analysis, etc.). Use/adoption metrics are important process outcomes that should be reported in any ehealth trial.

	1	2	3	4	5	
subitem not at all important	(•)	( )	( )	( )	( )	essential

Clear selection

Does your paper address subitem 6a-ii?

Copy and paste relevant sections from manuscript text

Your answer

6a-iii) Describe whether, how, and when qualitative feedback from participants was obtained

Describe whether, how, and when qualitative feedback from participants was obtained (e.g., through emails, feedback forms, interviews, focus groups).

	1	2	3	4	5	
subitem not at all important	(●)	( )	( )	( )	( )	essential

Clear selection

Does your paper address subitem 6a-iii?

Copy and paste relevant sections from manuscript text

Your answer

6b) Any changes to trial outcomes after the trial commenced, with reasons

Does your paper address CONSORT subitem 6b? *

Copy and paste relevant sections from the manuscript (include quotes in quotation marks "like this" to indicate direct quotes from your manuscript), or elaborate on this item by providing additional information not in the ms, or briefly explain why the item is not applicable/relevant for your study

No relevant manuscript in this paper

7a) How sample size was determined

NPT: When applicable, details of whether and how the clustering by care provides or centers was addressed

7a-i) Describe whether and how expected attrition was taken into account when calculating the sample size

Describe whether and how expected attrition was taken into account when calculating the sample size.

	1	2	3	4	5	
subitem not at all important	◉	○	○	○	○	essential

Clear selection

Does your paper address subitem 7a-i?

Copy and paste relevant sections from manuscript title (include quotes in quotation marks "like this" to indicate direct quotes from your manuscript), or elaborate on this item by providing additional information not in the ms, or briefly explain why the item is not applicable/relevant for your study

No direct manuscript quote was identified describing whether or how expected attrition was taken into account when calculating the sample size.

7b) When applicable, explanation of any interim analyses and stopping guidelines

Does your paper address CONSORT subitem 7b? *

Copy and paste relevant sections from the manuscript (include quotes in quotation marks "like this" to indicate direct quotes from your manuscript), or elaborate on this item by providing additional information not in the ms, or briefly explain why the item is not applicable/relevant for your study

not applicable in this paper

8a) Method used to generate the random allocation sequence

NPT: When applicable, how care providers were allocated to each trial group

Does your paper address CONSORT subitem 8a? *

Copy and paste relevant sections from the manuscript (include quotes in quotation marks "like this" to indicate direct quotes from your manuscript), or elaborate on this item by providing additional information not in the ms, or briefly explain why the item is not applicable/relevant for your study

The manuscript describes the method used to generate the random allocation sequence: "All 21 classes within the preschool were included as clusters and randomly assigned in a 1:1 ratio to either the intervention group (10 classes) or a waitlist control group (11 classes) using a computer-generated sequence by an independent researcher."

8b) Type of randomisation; details of any restriction (such as blocking and block size)

Does your paper address CONSORT subitem 8b? *

Copy and paste relevant sections from the manuscript (include quotes in quotation marks "like this" to indicate direct quotes from your manuscript), or elaborate on this item by providing additional information not in the ms, or briefly explain why the item is not applicable/relevant for your study

The manuscript describes cluster randomization and allocation ratio:
"All 21 classes within the preschool were included as clusters and randomly assigned in a 1:1 ratio to either the intervention group (10 classes) or a waitlist control group (11 classes) using a computer-generated sequence by an independent researcher."

The manuscript identifies the study as a cluster randomized controlled trial:
"The present study is a secondary analysis of data from a pragmatic, two-arm, single-blind cluster randomized controlled trial evaluating a universal digital-human parenting intervention delivered through the preschool system in China."

9) Mechanism used to implement the random allocation sequence (such as sequentially numbered containers), describing any steps taken to conceal the sequence until interventions were assigned

Does your paper address CONSORT subitem 9? *

Copy and paste relevant sections from the manuscript (include quotes in quotation marks "like this" to indicate direct quotes from your manuscript), or elaborate on this item by providing additional information not in the ms, or briefly explain why the item is not applicable/relevant for your study

The manuscript states that randomization used a computer-generated sequence by an independent researcher:
"All 21 classes within the preschool were included as clusters and randomly assigned in a 1:1 ratio to either the intervention group (10 classes) or a waitlist control group (11 classes) using a computer-generated sequence by an independent researcher

10) Who generated the random allocation sequence, who enrolled participants, and who assigned participants to interventions

Does your paper address CONSORT subitem 10? *

Copy and paste relevant sections from the manuscript (include quotes in quotation marks "like this" to indicate direct quotes from your manuscript), or elaborate on this item by providing additional information not in the ms, or briefly explain why the item is not applicable/relevant for your study

The manuscript identifies who generated the random allocation sequence:
"All 21 classes within the preschool were included as clusters and randomly assigned in a 1:1 ratio to either the intervention group (10 classes) or a waitlist control group (11 classes) using a computer-generated sequence by an independent researcher."

The manuscript describes the recruitment/enrollment channel:
"Caregivers were recruited through routine school communication channels, with one caregiver participating per family."

The manuscript reports group allocation:
"A total of 541 caregivers were enrolled, including 272 in the intervention group and 269 in the control group."

11a) If done, who was blinded after assignment to interventions (for example, participants, care providers, those assessing outcomes) and how

NPT: Whether or not administering co-interventions were blinded to group assignment

11a-i) Specify who was blinded, and who wasn’t

Specify who was blinded, and who wasn’t. Usually, in web-based trials it is not possible to blind the participants [1, 3] (this should be clearly acknowledged), but it may be possible to blind outcome assessors, those doing data analysis or those administering co-interventions (if any).

	1	2	3	4	5	
subitem not at all important	○	○	○	○	◉	essential

Clear selection

Does your paper address subitem 11a-i? *

Copy and paste relevant sections from the manuscript (include quotes in quotation marks "like this" to indicate direct quotes from your manuscript), or elaborate on this item by providing additional information not in the ms, or briefly explain why the item is not applicable/relevant for your study

The manuscript describes the trial as single-blind:
"The present study is a secondary analysis of data from a pragmatic, two-arm, single-blind cluster randomized controlled trial evaluating a universal digital-human parenting intervention delivered through the preschool system in China."
Parents,social workers and teachers are blinded

11a-ii) Discuss e.g., whether participants knew which intervention was the "intervention of interest" and which one was the "comparator"

Informed consent procedures (4a-ii) can create biases and certain expectations - discuss e.g., whether participants knew which intervention was the "intervention of interest" and which one was the "comparator".

	1	2	3	4	5	
subitem not at all important	◉	○	○	○	○	essential

Clear selection

Does your paper address subitem 11a-ii?

Copy and paste relevant sections from the manuscript (include quotes in quotation marks "like this" to indicate direct quotes from your manuscript), or elaborate on this item by providing additional information not in the ms, or briefly explain why the item is not applicable/relevant for your study

Your answer

11b) If relevant, description of the similarity of interventions

(this item is usually not relevant for ehealth trials as it refers to similarity of a placebo or sham intervention to a active medication/intervention)

Does your paper address CONSORT subitem 11b? *

Copy and paste relevant sections from the manuscript (include quotes in quotation marks "like this" to indicate direct quotes from your manuscript), or elaborate on this item by providing additional information not in the ms, or briefly explain why the item is not applicable/relevant for your study

The manuscript describes a waitlist control rather than a placebo/sham intervention: "The intervention group received the program following baseline assessment, while the waitlist control group received no formal support during this period and began the intervention after the post-intervention assessment."

12a) Statistical methods used to compare groups for primary and secondary outcomes

NPT: When applicable, details of whether and how the clustering by care providers or centers was addressed

Does your paper address CONSORT subitem 12a? *

Copy and paste relevant sections from the manuscript (include quotes in quotation marks "like this" to indicate direct quotes from your manuscript), or elaborate on this item by providing additional information not in the ms, or briefly explain why the item is not applicable/relevant for your study

ITT: "Moderation of intervention effectiveness at post-intervention was analyzed following the intention-to-treat principle."; model types/clustering: "We used multivariable mixed-effects models (linear, Poisson, or negative binomial, depending on outcome distribution) to account for clustering at the class level and repeated assessments within families (random intercepts)."; model selection: "Model selection for violence perpetration outcomes was informed by distribution test (Table S1)."; moderation: "Moderation by family size was assessed using a group ? time ? family size interaction term."; appendix: "Random intercepts for class (cluster) and family were included to account for clustering at the class level and repeated assessments within families."

12a-i) Imputation techniques to deal with attrition / missing values

Imputation techniques to deal with attrition / missing values: Not all participants will use the intervention/comparator as intended and attrition is typically high in ehealth trials. Specify how participants who did not use the application or dropped out from the trial were treated in the statistical analysis (a complete case analysis is strongly discouraged, and simple imputation techniques such as LOCF may also be problematic [4]).

	1	2	3	4	5	
subitem not at all important	○	○	○	○	◉	essential

Clear selection

Does your paper address subitem 12a-i? *

Copy and paste relevant sections from the manuscript (include quotes in quotation marks "like this" to indicate direct quotes from your manuscript), or elaborate on this item by providing additional information not in the ms, or briefly explain why the item is not applicable/relevant for your study

The manuscript reports how missing data were handled:
"Missing data were handled using listwise deletion with each model."

The appendix provides more detail on the complete-case approach:
"Participants with missing data on variables required for a given model were excluded from that specific analysis."

The appendix explains the rationale for this approach:
"This complete-case approach was used because missingness was limited and varied across model-specific covariates, and it allowed each model to be estimated using observed data without imputing values for baseline characteristics or outcome measures."

The appendix states that model-specific sample sizes were reported:
"Model-specific sample sizes are reported in the relevant tables."

12b) Methods for additional analyses, such as subgroup analyses and adjusted analyses

Does your paper address CONSORT subitem 12b? *

Copy and paste relevant sections from the manuscript (include quotes in quotation marks "like this" to indicate direct quotes from your manuscript), or elaborate on this item by providing additional information not in the ms, or briefly explain why the item is not applicable/relevant for your study

subgroup/sensitivity analyses: "Subgroup analyses stratified by number of children were also conducted, and sensitivity analyses treated family size as a continuous variable."; adjusted longitudinal analyses: "For the intervention group, longitudinal trajectories from T0 to T3 were examined using mixed-effects models including time, family size, and their interaction, adjusting for baseline sociodemographic covariates."; adjusted baseline analyses: "Multivariable regression models, adjusted for sociodemographic covariates, were used to examine baseline associations between number of children and outcomes."; appendix: "the moderation models were re-estimated with child number treated as a continuous variable."

X26) REB/IRB Approval and Ethical Considerations [recommended as subheading under "Methods"] (not a CONSORT item)

X26-i) Comment on ethics committee approval

	1	2	3	4	5	
subitem not at all important	○	○	○	○	◉	essential

Clear selection

Does your paper address subitem X26-i?

Copy and paste relevant sections from the manuscript (include quotes in quotation marks "like this" to indicate direct quotes from your manuscript), or elaborate on this item by providing additional information not in the ms, or briefly explain why the item is not applicable/relevant for your study

The manuscript reports ethics committee approval:
"Ethical approval for the original trial was obtained from Beijing Normal University (SSDPP-HSC-2024003) and the University of Oxford (SPI DREC 25 006)."

x26-ii) Outline informed consent procedures

Outline informed consent procedures e.g., if consent was obtained offline or online (how? Checkbox, etc.?), and what information was provided (see 4a-ii). See [6] for some items to be included in informed consent documents.

	1	2	3	4	5	
subitem not at all important	○	○	○	◉	○	essential

Clear selection

Does your paper address subitem X26-ii?

Copy and paste relevant sections from the manuscript (include quotes in quotation marks "like this" to indicate direct quotes from your manuscript), or elaborate on this item by providing additional information not in the ms, or briefly explain why the item is not applicable/relevant for your study

The manuscript states that informed consent was required for eligibility:
"Inclusion criteria were minimal to reflect the universal nature of the intervention: caregivers had to be aged 18 years or older, have access to a smartphone, and provide informed consent."

The manuscript states that informed consent was obtained before data collection:
"All participants provided informed consent prior to data collection."

X26-iii) Safety and security procedures

Safety and security procedures, incl. privacy considerations, and any steps taken to reduce the likelihood or detection of harm (e.g., education and training, availability of a hotline)

	1	2	3	4	5	
subitem not at all important	○	○	○	○	◉	essential

Clear selection

Does your paper address subitem X26-iii?

Copy and paste relevant sections from the manuscript (include quotes in quotation marks "like this" to indicate direct quotes from your manuscript), or elaborate on this item by providing additional information not in the ms, or briefly explain why the item is not applicable/relevant for your study

We strictly protect participants' information, obtain their consent during the process, and the collected data is stored on dedicated servers and strictly protected.

RESULTS

13a) For each group, the numbers of participants who were randomly assigned, received intended treatment, and were analysed for the primary outcome
NPT: The number of care providers or centers performing the intervention in each group and the number of patients treated by each care provider in each center

Does your paper address CONSORT subitem 13a? *

Copy and paste relevant sections from the manuscript (include quotes in quotation marks "like this" to indicate direct quotes from your manuscript), or elaborate on this item by providing additional information not in the ms, or briefly explain why the item is not applicable/relevant for your study

randomized/enrolled by group: "All 21 classes within the preschool were included as clusters and randomly assigned in a 1:1 ratio to either the intervention group (10 classes) or a waitlist control group (11 classes)"; "A total of 541 caregivers were enrolled, including 272 in the intervention group and 269 in the control group."; treatment received/timing: "The intervention group received the program following baseline assessment, while the waitlist control group received no formal support during this period"; analysis numbers: "Included in the Intent-to-Treat Analysis 1) immediate post-intervention: 21 clusters, n=541 2) 6-month follow-up: 10 clusters, n=213 3) 12-month follow-up: 10 clusters, n=158".

13b) For each group, losses and exclusions after randomisation, together with reasons

Does your paper address CONSORT subitem 13b? (NOTE: Preferably, this is shown in a CONSORT flow diagram) *

Copy and paste relevant sections from the manuscript (include quotes in quotation marks "like this" to indicate direct quotes from your manuscript), or elaborate on this item by providing additional information not in the ms, or briefly explain why the item is not applicable/relevant for your study

The appendix flowchart reports dropouts after randomization in the intervention group before immediate post-intervention assessment:
"Dropouts (n=26)"

The appendix flowchart reports dropouts after randomization in the waitlist control group before immediate post-intervention assessment:
"Dropouts (n=13)"

The appendix flowchart reports further dropouts in the intervention group before 6-month follow-up:
"Dropouts (n=59)"

The appendix flowchart reports further dropouts in the intervention group before 12-month follow-up:
"Dropouts (n=114)"

The appendix note clarifies how dropout numbers were counted:
"Note. At each follow-up, all participants originally allocated to the intervention group were invited to complete the assessment, regardless of prior participation. Dropout numbers were specific to each phase and not cumulative from earlier time points."

13b-i) Attrition diagram

Strongly recommended: An attrition diagram (e.g., proportion of participants still logging in or using the intervention/comparator in each group plotted over time, similar to a survival curve) or other figures or tables demonstrating usage/dose/engagement.

	1	2	3	4	5	
subitem not at all important	○	○	○	○	◉	essential

Clear selection

Does your paper address subitem 13b-i?

Copy and paste relevant sections from the manuscript or cite the figure number if applicable (include quotes in quotation marks "like this" to indicate direct quotes from your manuscript), or elaborate on this item by providing additional information not in the ms, or briefly explain why the item is not applicable/relevant for your study

participant flowchart: "Participant Flowchart"; invited/baseline: "Caregivers who were invited to participate (21 clusters, n=665)"; "Baseline Assessment (T0) (21 clusters, n=541)"; allocation: "Treatment Group (10 clusters, n=272)"; "Waitlist Control Group (11 clusters, n=269)"; dropouts: "Dropouts (n=26)"; "Dropouts (n=13)"; "Dropouts (n=59)"; "Dropouts (n=114)"; analysis: "Included in the Intent-to-Treat Analysis 1) immediate post-intervention: 21 clusters, n=541 2) 6-month follow-up: 10 clusters, n=213 3) 12-month follow-up: 10 clusters, n=158"; note: "Dropout numbers were specific to each phase and not cumulative from earlier time points."

14a) Dates defining the periods of recruitment and follow-up

Does your paper address CONSORT subitem 14a? *

Copy and paste relevant sections from the manuscript (include quotes in quotation marks "like this" to indicate direct quotes from your manuscript), or elaborate on this item by providing additional information not in the ms, or briefly explain why the item is not applicable/relevant for your study

The paper introduces the time points of the first data collection and the time intervals of subsequent data collection.

14a-i) Indicate if critical “secular events” fell into the study period

Indicate if critical “secular events” fell into the study period, e.g., significant changes in Internet resources available or “changes in computer hardware or Internet delivery resources”

	1	2	3	4	5	
subitem not at all important	(●)	( )	( )	( )	( )	essential

Clear selection

Does your paper address subitem 14a-i?

Copy and paste relevant sections from the manuscript (include quotes in quotation marks "like this" to indicate direct quotes from your manuscript), or elaborate on this item by providing additional information not in the ms, or briefly explain why the item is not applicable/relevant for your study

Your answer

14b) Why the trial ended or was stopped (early)

Does your paper address CONSORT subitem 14b? *

Copy and paste relevant sections from the manuscript (include quotes in quotation marks "like this" to indicate direct quotes from your manuscript), or elaborate on this item by providing additional information not in the ms, or briefly explain why the item is not applicable/relevant for your study

It is based on the study design,the intervention was 2.5 mounth.

15) A table showing baseline demographic and clinical characteristics for each group

NPT: When applicable, a description of care providers (case volume, qualification, expertise, etc.) and centers (volume) in each group

Does your paper address CONSORT subitem 15? *

Copy and paste relevant sections from the manuscript (include quotes in quotation marks "like this" to indicate direct quotes from your manuscript), or elaborate on this item by providing additional information not in the ms, or briefly explain why the item is not applicable/relevant for your study

baseline table/sample: "Table 1 shows baseline characteristics by number of children in the analytic full sample (N = 494)."; missing baseline data: "Of the 541 families enrolled at baseline, 47 were excluded from because baseline sociodemographic information required for the analysis was incomplete."; family-size groups: "120 (24.29%) families had one child, 340 (68.83%) had two children, and 34 (6.88%) had three or more children."; baseline demographics: "Most caregivers were married (98.38%), female (74.90%), of Han ethnicity (99.39%), and had urban hukou (67.81%)."; children: "56.07% were boys, and 24.09% had functional difficulties."; clusters: "All 21 classes within the preschool were included as clusters".

15-i) Report demographics associated with digital divide issues

In ehealth trials it is particularly important to report demographics associated with digital divide issues, such as age, education, gender, social-economic status, computer/Internet/ehealth literacy of the participants, if known.

	1	2	3	4	5	
subitem not at all important	○	○	○	○	◉	essential

Clear selection

Does your paper address subitem 15-i? *

Copy and paste relevant sections from the manuscript (include quotes in quotation marks "like this" to indicate direct quotes from your manuscript), or elaborate on this item by providing additional information not in the ms, or briefly explain why the item is not applicable/relevant for your study

age/gender/hukou: "Most caregivers were married (98.38%), female (74.90%), of Han ethnicity (99.39%), and had urban hukou (67.81%)."; age: "Caregiver age differed significantly by family size"; education: "Educational attainment was lower in larger families (3.94 vs 4.65 and 4.50, p < 0.001)."; employment: "Employment status also varied across groups (p = 0.003), with families with three or more children having the lowest proportion of full-time employment (55.88%) and the highest proportion of unemployment (23.53%)."; smartphone access: "caregivers had to be aged 18 years or older, have access to a smartphone, and provide informed consent."

16) For each group, number of participants (denominator) included in each analysis and whether the analysis was by original assigned groups

16-i) Report multiple “denominators” and provide definitions

Report multiple “denominators” and provide definitions: Report N’s (and effect sizes) “across a range of study participation [and use] thresholds” [1], e.g., N exposed, N consented, N used more than x times, N used more than y weeks, N participants “used” the intervention/comparator at specific pre-defined time points of interest (in absolute and relative numbers per group). Always clearly define “use” of the intervention.

	1	2	3	4	5	
subitem not at all important	○	○	○	◉	○	essential

Clear selection

Does your paper address subitem 16-i? *

Copy and paste relevant sections from the manuscript (include quotes in quotation marks "like this" to indicate direct quotes from your manuscript), or elaborate on this item by providing additional information not in the ms, or briefly explain why the item is not applicable/relevant for your study

enrolled denominator: "A total of 541 caregivers were enrolled, including 272 in the intervention group and 269 in the control group."; analytic baseline denominator: "Table 1 shows baseline characteristics by number of children in the analytic full sample (N = 494)."; reason for difference: "Of the 541 families enrolled at baseline, 47 were excluded from because baseline sociodemographic information required for the analysis was incomplete."; ITT/follow-up denominators: "Included in the Intent-to-Treat Analysis 1) immediate post-intervention: 21 clusters, n=541 2) 6-month follow-up: 10 clusters, n=213 3) 12-month follow-up: 10 clusters, n=158"; dropout note: "Dropout numbers were specific to each phase and not cumulative from earlier time points."

16-ii) Primary analysis should be intent-to-treat

Primary analysis should be intent-to-treat, secondary analyses could include comparing only “users”, with the appropriate caveats that this is no longer a randomized sample (see 18-i).

	1	2	3	4	5	
subitem not at all important	○	○	◉	○	○	essential

Clear selection

Does your paper address subitem 16-ii?

Copy and paste relevant sections from the manuscript (include quotes in quotation marks "like this" to indicate direct quotes from your manuscript), or elaborate on this item by providing additional information not in the ms, or briefly explain why the item is not applicable/relevant for your study

The manuscript states that the post-intervention analysis followed the intention-to-treat principle:
"Moderation of intervention effectiveness at post-intervention was analyzed following the intention-to-treat principle."

The appendix states that participants were analyzed according to original group assignment:
"The post-intervention effectiveness analysis followed the intention-to-treat design, with participants analyzed according to their original group assignment."

The appendix flowchart reports intent-to-treat analysis denominators:
"Included in the Intent-to-Treat Analysis 1) immediate post-intervention: 21 clusters, n=541 2) 6-month follow-up: 10 clusters, n=213 3) 12-month follow-up: 10 clusters, n=158"

17a) For each primary and secondary outcome, results for each group, and the estimated effect size and its precision (such as 95% confidence interval)

Does your paper address CONSORT subitem 17a? *

Copy and paste relevant sections from the manuscript (include quotes in quotation marks "like this" to indicate direct quotes from your manuscript), or elaborate on this item by providing additional information not in the ms, or briefly explain why the item is not applicable/relevant for your study

outcome tables/effects/CIs: "Table 1 shows baseline characteristics by number of children in the analytic full sample (N = 494)."; "Table 2 presents the baseline associations between number of children and parental and child outcomes in the analytic sample."; "Moderator analyses showed no evidence that immediate post-intervention effects on the primary outcomes differed by number of children (Table 3)."; "In subgroup analyses stratified by number of children, patterns of intervention effects varied across outcome domains (Table 4)."; "For the primary outcomes, significant time ? number of children interactions were observed for early learning and stimulation and caregiver-perpetrated violence (Table 5)."; example estimate: "two-child families showed greater improvement at T2 (b = 3.966, 95% CI: 1.468 to 6.463, p = .002)".

17a-i) Presentation of process outcomes such as metrics of use and intensity of use

In addition to primary/secondary (clinical) outcomes, the presentation of process outcomes such as metrics of use and intensity of use (dose, exposure) and their operational definitions is critical. This does not only refer to metrics of attrition (13-b) (often a binary variable), but also to more continuous exposure metrics such as “average session length”. These must be accompanied by a technical description how a metric like a “session” is defined (e.g., timeout after idle time) [1] (report under item 6a).

	1	2	3	4	5	
subitem not at all important	(•)	( )	( )	( )	( )	essential

Clear selection

Does your paper address subitem 17a-i?

Copy and paste relevant sections from the manuscript (include quotes in quotation marks "like this" to indicate direct quotes from your manuscript), or elaborate on this item by providing additional information not in the ms, or briefly explain why the item is not applicable/relevant for your study

Your answer

17b) For binary outcomes, presentation of both absolute and relative effect sizes is recommended

Does your paper address CONSORT subitem 17b? *

Copy and paste relevant sections from the manuscript (include quotes in quotation marks "like this" to indicate direct quotes from your manuscript), or elaborate on this item by providing additional information not in the ms, or briefly explain why the item is not applicable/relevant for your study

Not applicable in this paper

18) Results of any other analyses performed, including subgroup analyses and adjusted analyses, distinguishing pre-specified from exploratory

Does your paper address CONSORT subitem 18? *

Copy and paste relevant sections from the manuscript (include quotes in quotation marks "like this" to indicate direct quotes from your manuscript), or elaborate on this item by providing additional information not in the ms, or briefly explain why the item is not applicable/relevant for your study

subgroup analyses: "In subgroup analyses stratified by number of children, patterns of intervention effects varied across outcome domains (Table 4)."; sensitivity analyses: "As a sensitivity analysis, number of children was modeled as a continuous moderator, and models including a group ? time ? number of children interaction were re-estimated (Table S2)."; adjusted longitudinal analyses: "For the intervention group, longitudinal trajectories from T0 to T3 were examined using mixed-effects models including time, family size, and their interaction, adjusting for baseline sociodemographic covariates."; unadjusted sensitivity: "For sensitivity analyses, we re-estimated unadjusted longitudinal models including time, number of children, and their interaction (Tables S6 and S7)."

18-i) Subgroup analysis of comparing only users

A subgroup analysis of comparing only users is not uncommon in ehealth trials, but if done, it must be stressed that this is a self-selected sample and no longer an unbiased sample from a randomized trial (see 16-iii).

	1	2	3	4	5	
subitem not at all important	(●)	○	○	○	○	essential

Clear selection

Does your paper address subitem 18-i?

Copy and paste relevant sections from the manuscript (include quotes in quotation marks "like this" to indicate direct quotes from your manuscript), or elaborate on this item by providing additional information not in the ms, or briefly explain why the item is not applicable/relevant for your study

Not applicable.The manuscript reports subgroup analyses by number of children, not by user status:
"Subgroup analyses stratified by number of children were also conducted, and sensitivity analyses treated family size as a continuous variable."

19) All important harms or unintended effects in each group
(for specific guidance see CONSORT for harms)

Does your paper address CONSORT subitem 19? *

Copy and paste relevant sections from the manuscript (include quotes in quotation marks "like this" to indicate direct quotes from your manuscript), or elaborate on this item by providing additional information not in the ms, or briefly explain why the item is not applicable/relevant for your study

No harm we observed,beacause our intervention is evidenced and used in many countaries.

19-i) Include privacy breaches, technical problems

Include privacy breaches, technical problems. This does not only include physical “harm” to participants, but also incidents such as perceived or real privacy breaches [1], technical problems, and other unexpected/unintended incidents. “Unintended effects” also includes unintended positive effects [2].

	1	2	3	4	5	
subitem not at all important	●	○	○	○	○	essential

Clear selection

Does your paper address subitem 19-i?

Copy and paste relevant sections from the manuscript (include quotes in quotation marks "like this" to indicate direct quotes from your manuscript), or elaborate on this item by providing additional information not in the ms, or briefly explain why the item is not applicable/relevant for your study

Your answer

19-ii) Include qualitative feedback from participants or observations from staff/researchers

Include qualitative feedback from participants or observations from staff/researchers, if available, on strengths and shortcomings of the application, especially if they point to unintended/unexpected effects or uses. This includes (if available) reasons for why people did or did not use the application as intended by the developers.

	1	2	3	4	5	
subitem not at all important	(•)	( )	( )	( )	( )	essential

Clear selection

Does your paper address subitem 19-ii?

Copy and paste relevant sections from the manuscript (include quotes in quotation marks "like this" to indicate direct quotes from your manuscript), or elaborate on this item by providing additional information not in the ms, or briefly explain why the item is not applicable/relevant for your study

We reported the qualitative result in other paper(Institutionalizing Digital Parenting Programs in Low Resource Settings in China: Comparative Case Study of Health Care and Education Sectors Using the RE-AIM Framework,2026,JMIR)

DISCUSSION

22) Interpretation consistent with results, balancing benefits and harms, and considering other relevant evidence

NPT: In addition, take into account the choice of the comparator, lack of or partial blinding, and unequal expertise of care providers or centers in each group

22-i) Restate study questions and summarize the answers suggested by the data, starting with primary outcomes and process outcomes (use)

Restate study questions and summarize the answers suggested by the data, starting with primary outcomes and process outcomes (use).

	1	2	3	4	5	
subitem not at all important	○	○	○	○	◉	essential

Clear selection

Does your paper address subitem 22-i? *

Copy and paste relevant sections from the manuscript (include quotes in quotation marks "like this" to indicate direct quotes from your manuscript), or elaborate on this item by providing additional information not in the ms, or briefly explain why the item is not applicable/relevant for your study

study question: "This study examined whether the number of children is associated with baseline differences and heterogeneous responses to a universal digital-human parenting intervention delivered in a preschool setting."; baseline answer: "First, family size was associated with systematic baseline differences"; immediate effects: "Second, family size did not significantly moderate immediate post-intervention effects. The program showed similar short-term effectiveness across one-child, two-child, and three-or-more-child families."; trajectories: "Third, differences emerged in post-intervention trajectories."; "two-child families showed the most consistent improvements, three-or-more-child families showed larger but more variable gains in some domains, and one-child families showed more limited changes."

22-ii) Highlight unanswered new questions, suggest future research

Highlight unanswered new questions, suggest future research.

	1	2	3	4	5	
subitem not at all important	○	○	○	◉	○	essential

Clear selection

Does your paper address subitem 22-ii?

Copy and paste relevant sections from the manuscript (include quotes in quotation marks "like this" to indicate direct quotes from your manuscript), or elaborate on this item by providing additional information not in the ms, or briefly explain why the item is not applicable/relevant for your study

The manuscript suggests future research on other contextual factors:
"Fourth, number of children captures only one dimension of family size and caregiving complexity; other contextual factors, such as co-parenting dynamics, may also shape intervention processes and should be examined in future research."

The manuscript suggests more context-sensitive research approaches:
"First, this study highlights the importance of moving beyond treating family structure as a control variable toward recognizing it as a meaningful dimension shaping intervention process."

The manuscript identifies the need for research integrating heterogeneity and longitudinal dynamics:
"The observed differences in baseline conditions and post-intervention trajectories underscore the need for more context-sensitive research approaches that integrate heterogeneity with longitudinal dynamics."

20) Trial limitations, addressing sources of potential bias, imprecision, and, if relevant, multiplicity of analyses

20-i) Typical limitations in ehealth trials

Typical limitations in ehealth trials: Participants in ehealth trials are rarely blinded. Ehealth trials often look at a multiplicity of outcomes, increasing risk for a Type I error. Discuss biases due to non-use of the intervention/usability issues, biases through informed consent procedures, unexpected events.

	1	2	3	4	5	
subitem not at all important	○	○	○	○	◉	essential

Clear selection

Does your paper address subitem 20-i? *

Copy and paste relevant sections from the manuscript (include quotes in quotation marks "like this" to indicate direct quotes from your manuscript), or elaborate on this item by providing additional information not in the ms, or briefly explain why the item is not applicable/relevant for your study

limited power: "First, the relatively small number of families with three or more children may have limited the power of analysis."; self-report bias: "Second, outcomes were based on caregiver self-report and may be subject to reporting bias, especially for sensitive behaviors such as violent parenting."; waitlist/causal inference: "Third, due to the waitlist design, longer-term follow-up data were available only for the intervention group, limiting causal inference on sustained effects."; other context: "other contextual factors, such as co-parenting dynamics, may also shape intervention processes and should be examined in future research."

21) Generalisability (external validity, applicability) of the trial findings

NPT: External validity of the trial findings according to the intervention, comparators, patients, and care providers or centers involved in the trial

21-i) Generalizability to other populations

Generalizability to other populations: In particular, discuss generalizability to a general Internet population, outside of a RCT setting, and general patient population, including applicability of the study results for other organizations

	1	2	3	4	5	
subitem not at all important	○	○	○	●	○	essential

Clear selection

Does your paper address subitem 21-i?

Copy and paste relevant sections from the manuscript (include quotes in quotation marks "like this" to indicate direct quotes from your manuscript), or elaborate on this item by providing additional information not in the ms, or briefly explain why the item is not applicable/relevant for your study

broader relevance: "More broadly, these findings have implications beyond Chinese context."; other settings: "As family structures evolve in many settings, including through declining fertility, delayed childbearing, and changing caregiving arrangements, variation in family size may interact with social and institutional contexts to shape how interventions are implemented and sustained."; intervention design: "Understanding these contextual dynamics is important for designing parenting interventions that are both scalable and responsive to diverse family needs."; policy/services: "policies and services need to better align with evolving family structures."

21-ii) Discuss if there were elements in the RCT that would be different in a routine application setting

Discuss if there were elements in the RCT that would be different in a routine application setting (e.g., prompts/reminders, more human involvement, training sessions or other co-interventions) and what impact the omission of these elements could have on use, adoption, or outcomes if the intervention is applied outside of a RCT setting.

	1	2	3	4	5	
subitem not at all important	○	○	○	◉	○	essential

Clear selection

Does your paper address subitem 21-ii?

Copy and paste relevant sections from the manuscript (include quotes in quotation marks "like this" to indicate direct quotes from your manuscript), or elaborate on this item by providing additional information not in the ms, or briefly explain why the item is not applicable/relevant for your study

RCT/routine differences are not directly compared, but relevant elements are discussed. Scalability/human support: "relying more heavily on self-directed engagement with structured content and less intensive human support"; engagement burden: "Caregivers managing multiple children may face greater challenges in consistently engaging with materials"; routine support: "booster sessions, ongoing digital reminders, or structured follow-up support"; tailored support: "Differentiated pacing, targeted support for families with multiple children, or guidance on managing competing caregiving demands may help reduce implementation burden while preserving scalability."

OTHER INFORMATION

23) Registration number and name of trial registry

Does your paper address CONSORT subitem 23? *

Copy and paste relevant sections from the manuscript (include quotes in quotation marks "like this" to indicate direct quotes from your manuscript), or elaborate on this item by providing additional information not in the ms, or briefly explain why the item is not applicable/relevant for your study

The manuscript reports the trial registry and registration number:
"The trial was prospectively registered on the Chinese Clinical Trial Registry (ChiCTR2400081911)."

24) Where the full trial protocol can be accessed, if available

Does your paper address CONSORT subitem 24? *

Cite a Multimedia Appendix, other reference, or copy and paste relevant sections from the manuscript (include quotes in quotation marks "like this" to indicate direct quotes from your manuscript), or elaborate on this item by providing additional information not in the ms, or briefly explain why the item is not applicable/relevant for your study

It can be found in the supporting information of the paper named "Effectiveness of a universal digital–human parenting intervention in promoting early childhood development and protection: A pragmatic cluster randomized controlled trial"（https://doi.org/10.1371/journal.pdig.0001357.s003）

25) Sources of funding and other support (such as supply of drugs), role of funders

Does your paper address CONSORT subitem 25? *

Copy and paste relevant sections from the manuscript (include quotes in quotation marks "like this" to indicate direct quotes from your manuscript), or elaborate on this item by providing additional information not in the ms, or briefly explain why the item is not applicable/relevant for your study

The manuscript reports the funding sources:
"China Postdoctoral Science Foundation (2022M720467), Peking University Start-Up Research Grant, LEGO Foundation"

X27) Conflicts of Interest (not a CONSORT item)

X27-i) State the relation of the study team towards the system being evaluated

In addition to the usual declaration of interests (financial or otherwise), also state the relation of the study team towards the system being evaluated, i.e., state if the authors/evaluators are distinct from or identical with the developers/sponsors of the intervention.

	1	2	3	4	5	
subitem not at all important	○	○	○	○	◉	essential

Clear selection

Does your paper address subitem X27-i?

Copy and paste relevant sections from the manuscript (include quotes in quotation marks "like this" to indicate direct quotes from your manuscript), or elaborate on this item by providing additional information not in the ms, or briefly explain why the item is not applicable/relevant for your study

The manuscript discloses a relationship between JML and PLH:
"JML is the CEO of Parenting for Lifelong Health (PLH), a charitable organization based in the United Kingdom that developed the adapted program."

The manuscript reports the licensing status of PLH programs:
"PLH programs are open access and licensed under a Creative Commons 4.0 Attribution Share-Alike license."

The manuscript discloses ZF's prior consultancy relationship with PLH:
"ZF has worked as a consultant for PLH in the past."

The manuscript discloses JML's involvement in related research and institutional funding:
"JML has (and is participating) in a number of research studies involving the program, as an investigator, and the University of Oxford, and University of Cape Town receive research funding for these."

About the CONSORT EHEALTH checklist

As a result of using this checklist, did you make changes in your manuscript? *

- ( ) yes, major changes
- (•) yes, minor changes
- ( ) no

What were the most important changes you made as a result of using this checklist?

Your answer

How much time did you spend on going through the checklist INCLUDING making changes in your manuscript *

Almost six hours to be used to finish the checklist

As a result of using this checklist, do you think your manuscript has improved? *

- (●) yes
- ( ) no
- ( ) Other:

Would you like to become involved in the CONSORT EHEALTH group?

This would involve for example becoming involved in participating in a workshop and writing an "Explanation and Elaboration" document

- ( ) yes
- (●) no
- ( ) Other:

Clear selection

Any other comments or questions on CONSORT EHEALTH

Your answer

STOP - Save this form as PDF before you click submit

To generate a record that you filled in this form, we recommend to generate a PDF of this page (on a Mac, simply select "print" and then select "print as PDF") before you submit it.

When you submit your (revised) paper to JMIR, please upload the PDF as supplementary file.

Don't worry if some text in the textboxes is cut off, as we still have the complete information in our database. Thank you!

Final step: Click submit !

Click submit so we have your answers in our database!

Submit

Clear form

Never submit passwords through Google Forms.

This content is neither created nor endorsed by Google. - Contact form owner - Terms of Service - Privacy Policy

Does this form look suspicious? Report

Google Forms
